# Novel genes and sex differences in COVID-19 severity

**DOI:** 10.1093/hmg/ddac132

**Published:** 2022-06-16

**Authors:** Raquel Cruz, Silvia Diz-de Almeida, Miguel López de Heredia, Inés Quintela, Francisco C Ceballos, Guillermo Pita, José M Lorenzo-Salazar, Rafaela González-Montelongo, Manuela Gago-Domínguez, Marta Sevilla Porras, Jair Antonio Tenorio Castaño, Julian Nevado, Jose María Aguado, Carlos Aguilar, Sergio Aguilera-Albesa, Virginia Almadana, Berta Almoguera, Nuria Alvarez, Álvaro Andreu-Bernabeu, Eunate Arana-Arri, Celso Arango, María J Arranz, Maria-Jesus Artiga, Raúl C Baptista-Rosas, María Barreda-Sánchez, Moncef Belhassen-Garcia, Joao F Bezerra, Marcos A C Bezerra, Lucía Boix-Palop, María Brion, Ramón Brugada, Matilde Bustos, Enrique J Calderón, Cristina Carbonell, Luis Castano, Jose E Castelao, Rosa Conde-Vicente, M Lourdes Cordero-Lorenzana, Jose L Cortes-Sanchez, Marta Corton, M Teresa Darnaude, Alba De Martino-Rodríguez, Victor del Campo-Pérez, Aranzazu Diaz de Bustamante, Elena Domínguez-Garrido, Andre D Luchessi, Rocío Eiros, Gladys Mercedes Estigarribia Sanabria, María Carmen Fariñas, Uxía Fernández-Robelo, Amanda Fernández-Rodríguez, Tania Fernández-Villa, Belén Gil-Fournier, Javier Gómez-Arrue, Beatriz González Álvarez, Fernan Gonzalez Bernaldo de Quirós, Javier González-Peñas, Juan F Gutiérrez-Bautista, María José Herrero, Antonio Herrero-Gonzalez, María A Jimenez-Sousa, María Claudia Lattig, Anabel Liger Borja, Rosario Lopez-Rodriguez, Esther Mancebo, Caridad Martín-López, Vicente Martín, Oscar Martinez-Nieto, Iciar Martinez-Lopez, Michel F Martinez-Resendez, Angel Martinez-Perez, Juliana F Mazzeu, Eleuterio Merayo Macías, Pablo Minguez, Victor Moreno Cuerda, Vivian N Silbiger, Silviene F Oliveira, Eva Ortega-Paino, Mara Parellada, Estela Paz-Artal, Ney P C Santos, Patricia Pérez-Matute, Patricia Perez, M Elena Pérez-Tomás, Teresa Perucho, Mel Lina Pinsach-Abuin, Ericka N Pompa-Mera, Gloria L Porras-Hurtado, Aurora Pujol, Soraya Ramiro León, Salvador Resino, Marianne R Fernandes, Emilio Rodríguez-Ruiz, Fernando Rodriguez-Artalejo, José A Rodriguez-Garcia, Francisco Ruiz Cabello, Javier Ruiz-Hornillos, Pablo Ryan, José Manuel Soria, Juan Carlos Souto, Eduardo Tamayo, Alvaro Tamayo-Velasco, Juan Carlos Taracido-Fernandez, Alejandro Teper, Lilian Torres-Tobar, Miguel Urioste, Juan Valencia-Ramos, Zuleima Yáñez, Ruth Zarate, Tomoko Nakanishi, Sara Pigazzini, Frauke Degenhardt, Guillaume Butler-Laporte, Douglas Maya-Miles, Luis Bujanda, Youssef Bouysran, Adriana Palom, David Ellinghaus, Manuel Martínez-Bueno, Selina Rolker, Sara Amitrano, Luisa Roade, Francesca Fava, Christoph D Spinner, Daniele Prati, David Bernardo, Federico Garcia, Gilles Darcis, Israel Fernández-Cadenas, Jan Cato Holter, Jesus M Banales, Robert Frithiof, Stefano Duga, Rosanna Asselta, Alexandre C Pereira, Manuel Romero-Gómez, Beatriz Nafría-Jiménez, Johannes R Hov, Isabelle Migeotte, Alessandra Renieri, Anna M Planas, Kerstin U Ludwig, Maria Buti, Souad Rahmouni, Marta E Alarcón-Riquelme, Eva C Schulte, Andre Franke, Tom H Karlsen, Luca Valenti, Hugo Zeberg, Brent Richards, Andrea Ganna, Mercè Boada, Itziar de Rojas, Agustín Ruiz, Pascual Sánchez-Juan, Luis Miguel Real, SCOURGE Cohort Group, HOSTAGE Cohort Group, GRA@CE Cohort Group, Encarna Guillen-Navarro, Carmen Ayuso, Anna González-Neira, José A Riancho, Augusto Rojas-Martinez, Carlos Flores, Pablo Lapunzina, Angel Carracedo

**Affiliations:** Centro Nacional de Genotipado (CEGEN), Universidade de Santiago de Compostela, 15706 Santiago de Compostela, Spain; Centre for Biomedical Network Research on Rare Diseases (CIBERER), Instituto de Salud Carlos III, 28029 Madrid, Spain; Instituto de Investigación Sanitaria de Santiago (IDIS), 15706 Santiago de Compostela, Spain; Centro Singular de Investigación en Medicina Molecular y Enfermedades Crónicas (CIMUS), Universidade de Santiago de Compostela, 15782 Santiago de Compostela, Spain; Centro Singular de Investigación en Medicina Molecular y Enfermedades Crónicas (CIMUS), Universidade de Santiago de Compostela, 15782 Santiago de Compostela, Spain; Centre for Biomedical Network Research on Rare Diseases (CIBERER), Instituto de Salud Carlos III, 28029 Madrid, Spain; Centro Nacional de Genotipado (CEGEN), Universidade de Santiago de Compostela, 15706 Santiago de Compostela, Spain; Unidad de Infección Viral e Inmunidad, Centro Nacional de Microbiología (CNM), Instituto de Salud Carlos III (ISCIII), 28220 Madrid, Spain; Spanish National Cancer Research Centre, Human Genotyping-CEGEN Unit, 28029 Madrid, Spain; Genomics Division, Instituto Tecnológico y de Energías Renovables, 38600 Santa Cruz de Tenerife, Spain; Genomics Division, Instituto Tecnológico y de Energías Renovables, 38600 Santa Cruz de Tenerife, Spain; Fundación Pública Galega de Medicina Xenómica, Sistema Galego de Saúde (SERGAS), 15706 Santiago de Compostela, Spain; Instituto de Investigación Sanitaria de Santiago (IDIS), 15706 Santiago de Compostela, Spain; Centre for Biomedical Network Research on Rare Diseases (CIBERER), Instituto de Salud Carlos III, 28029 Madrid, Spain; Instituto de Genética Médica y Molecular (INGEMM), Hospital Universitario La Paz-IDIPAZ, 28046 Madrid, Spain; Centre for Biomedical Network Research on Rare Diseases (CIBERER), Instituto de Salud Carlos III, 28029 Madrid, Spain; Instituto de Genética Médica y Molecular (INGEMM), Hospital Universitario La Paz-IDIPAZ, 28046 Madrid, Spain; ERN-ITHACA-European Reference Network; Centre for Biomedical Network Research on Rare Diseases (CIBERER), Instituto de Salud Carlos III, 28029 Madrid, Spain; Instituto de Genética Médica y Molecular (INGEMM), Hospital Universitario La Paz-IDIPAZ, 28046 Madrid, Spain; ERN-ITHACA-European Reference Network; Unit of Infectious Diseases, Hospital Universitario 12 de Octubre, Instituto de Investigación Sanitaria Hospital 12 de Octubre (imas12), 28041 Madrid, Spain; Spanish Network for Research in Infectious Diseases (REIPI RD16/0016/0002), Instituto de Salud Carlos III, 28029 Madrid, Spain; School of Medicine, Universidad Complutense, 28040 Madrid, Spain; Centro de Investigación Biomédica en Red de Enfermedades Infecciosas (CIBERINFEC), Instituto de Salud Carlos III, 28029 Madrid, Spain; Hospital General Santa Bárbara de Soria, 42005 Soria, Spain; Pediatric Neurology Unit, Department of Pediatrics, Navarra Health Service Hospital, 31008 Pamplona, Spain; Navarra Health Service, NavarraBioMed Research Group, 31008 Pamplona, Spain; Hospital Universitario Virgen Macarena, Neumología, 41009 Seville, Spain; Department of Genetics & Genomics, Instituto de Investigación Sanitaria-Fundación Jiménez Díaz University Hospital - Universidad Autónoma de Madrid (IIS-FJD, UAM), 28040 Madrid, Spain; Centre for Biomedical Network Research on Rare Diseases (CIBERER), Instituto de Salud Carlos III, 28029 Madrid, Spain; Spanish National Cancer Research Centre, Human Genotyping-CEGEN Unit, 28029 Madrid, Spain; Department of Child and Adolescent Psychiatry, Institute of Psychiatry and Mental Health, Hospital General Universitario Gregorio Marañón (IiSGM), 28007 Madrid, Spain; School of Medicine, Universidad Complutense, 28040 Madrid, Spain; Biocruces Bizkai HRI, 48903 Barakaldo, Bizkaia, Spain; Cruces University Hospital, Osakidetza, 48903 Barakaldo, Bizkaia, Spain; Department of Child and Adolescent Psychiatry, Institute of Psychiatry and Mental Health, Hospital General Universitario Gregorio Marañón (IiSGM), 28007 Madrid, Spain; Centre for Biomedical Network Research on Mental Health (CIBERSAM), Instituto de Salud Carlos III, 28029 Madrid, Spain; School of Medicine, Universidad Complutense, 28040 Madrid, Spain; Fundació Docència I Recerca Mutua Terrassa, 08221 Terrassa, Spain; Spanish National Cancer Research Center, CNIO Biobank, 28029 Madrid, Spain; Hospital General de Occidente, 45170 Zapopan, Jalisco, Mexico; Centro Universitario de Tonalá, Universidad de Guadalajara, 45425 Tonalá, Jalisco, Mexico; Centro de Investigación Multidisciplinario en Salud, Universidad de Guadalajara, 45425 Tonalá, Jalisco, Mexico; Instituto Murciano de Investigación Biosanitaria (IMIB-Arrixaca), 30120 Murcia, Spain; Universidad Católica San Antonio de Murcia (UCAM), 30120 Murcia, Spain; Hospital Universitario de Salamanca-IBSAL, Servicio de Medicina Interna-Unidad de Enfermedades Infecciosas, 37007 Salamanca, Spain; Universidad de Salamanca, 37007 Salamanca, Spain; Escola Tecnica de Saúde, Laboratorio de Vigilancia Molecular Aplicada, 68515-000 Pará, Brazil; Federal University of Pernambuco, Genetics Postgraduate Program, Recife 50670-907, PE, Brazil; Hospital Universitario Mutua Terrassa, 08221 Terrassa, Spain; Instituto de Investigación Sanitaria de Santiago (IDIS), Xenética Cardiovascular, 15706 Santiago de Compostela, Spain; Centre for Biomedical Network Research on Cardiovascular Diseases (CIBERCV), Instituto de Salud Carlos III, 28029 Madrid, Spain; Cardiovascular Genetics Center, Institut d’Investigació Biomèdica Girona (IDIBGI), 17190 Girona, Spain; Medical Science Department, School of Medicine, University of Girona, 17190 Girona, Spain; Centre for Biomedical Network Research on Cardiovascular Diseases (CIBERCV), Instituto de Salud Carlos III, 28029 Madrid, Spain; Hospital Josep Trueta, Cardiology Service, 17190 Girona, Spain; Institute of Biomedicine of Seville (IBiS), Consejo Superior de Investigaciones Científicas (CSIC)- University of Seville- Virgen del Rocio University Hospital, 41013 Seville, Spain; Departemento de Medicina, Hospital Universitario Virgen del Rocío, Universidad de Sevilla, 41013 Seville, Spain; Centre for Biomedical Network Research on Epidemiology and Public Health (CIBERESP), Instituto de Salud Carlos III, 28029 Madrid, Spain; Instituto de Biomedicina de Sevilla, 41013 Seville, Spain; Hospital Universitario de Salamanca-IBSAL, Servicio de Medicina Interna, 37007 Salamanca, Spain; Universidad de Salamanca, 37007 Salamanca, Spain; Biocruces Bizkai HRI, 48903 Barakaldo, Bizkaia, Spain; Osakidetza, Cruces University Hospital, 48903 Barakaldo, Bizkaia, Spain; Centre for Biomedical Network Research on Rare Diseases (CIBERER), Instituto de Salud Carlos III, 28029 Madrid, Spain; Centre for Biomedical Network Research on Diabetes and Metabolic Associated Diseases (CIBERDEM), Instituto de Salud Carlos III, 28029 Madrid, Spain; University of Pais Vasco, UPV/EHU, 48903 Bizkaia, Spain; Oncology and Genetics Unit, Instituto de Investigacion Sanitaria Galicia Sur, Xerencia de Xestion Integrada de Vigo-Servizo Galego de Saúde, 36312 Vigo, Spain; Hospital Universitario Río Hortega, 47012 Valladolid, Spain; Servicio de Medicina intensiva, Complejo Hospitalario Universitario de A Coruña (CHUAC), Sistema Galego de Saúde (SERGAS), 15009 A Coruña, Spain; Tecnológico de Monterrey, 64718 Monterrey, Mexico; Otto von Guericke University, Departament of Microgravity and Translational Regenerative Medicine, 39106 Magdeburg, Germany; Department of Genetics & Genomics, Instituto de Investigación Sanitaria-Fundación Jiménez Díaz University Hospital - Universidad Autónoma de Madrid (IIS-FJD, UAM), 28040 Madrid, Spain; Centre for Biomedical Network Research on Rare Diseases (CIBERER), Instituto de Salud Carlos III, 28029 Madrid, Spain; Hospital Universitario Mostoles, Unidad de Genética, 28935 Madrid, Spain; Instituto Aragonés de Ciencias de la Salud (IACS), 50009 Zaragoza, Spain; Instituto Investigación Sanitaria Aragón (IIS-Aragon), 50009 Zaragoza, Spain; Preventive Medicine Department, Instituto de Investigacion Sanitaria Galicia Sur, Xerencia de Xestion Integrada de Vigo-Servizo Galego de Saúde, 36312 Vigo, Spain; Hospital Universitario Mostoles, Unidad de Genética, 28935 Madrid, Spain; Unidad Diagnóstico Molecular. Fundación Rioja Salud, 26006 La Rioja, Spain; Universidade Federal do Rio Grande do Norte, Departamento de Analises Clinicas e Toxicologicas, 59012-570 Natal, Brazil; Hospital Universitario de Salamanca-IBSAL, Servicio de Cardiología, 37007 Salamanca, Spain; Instituto Regional de Investigación en Salud-Universidad Nacional de Caaguazú, HH36+J3Q Caaguazú, Paraguay; IDIVAL, 39008 Cantabria, Spain; Universidad de Cantabria, 39008 Cantabria, Spain; Hospital U M Valdecilla, 39008 Cantabria, Spain; Urgencias Hospitalarias, Complejo Hospitalario Universitario de A Coruña (CHUAC), Sistema Galego de Saúde (SERGAS), 15009 A Coruña, Spain; Unidad de Infección Viral e Inmunidad, Centro Nacional de Microbiología (CNM), Instituto de Salud Carlos III (ISCIII), 28220 Madrid, Spain; Centro de Investigación Biomédica en Red de Enfermedades Infecciosas (CIBERINFEC), Instituto de Salud Carlos III, 28029 Madrid, Spain; Grupo de Investigación en Interacciones Gen-Ambiente y Salud (GIIGAS) - Instituto de Biomedicina (IBIOMED), Universidad de León, 24071 León, Spain; Hospital Universitario de Getafe, Servicio de Genética, 28905 Madrid, Spain; Instituto Aragonés de Ciencias de la Salud (IACS), 50009 Zaragoza, Spain; Instituto Investigación Sanitaria Aragón (IIS-Aragon), 50009 Zaragoza, Spain; Instituto Aragonés de Ciencias de la Salud (IACS), 50009 Zaragoza, Spain; Instituto Investigación Sanitaria Aragón (IIS-Aragon), 50009 Zaragoza, Spain; Ministerio de Salud Ciudad de Buenos Aires, Buenos Aires C1425EFD CABA, Argentina; Department of Child and Adolescent Psychiatry, Institute of Psychiatry and Mental Health, Hospital General Universitario Gregorio Marañón (IiSGM), 28007 Madrid, Spain; School of Medicine, Universidad Complutense, 28040 Madrid, Spain; Centre for Biomedical Network Research on Mental Health (CIBERSAM), Instituto de Salud Carlos III, 28029 Madrid, Spain; Hospital Universitario Virgen de las Nieves, Servicio de Análisis Clínicos e Inmunología, 18014 Granada, Spain; IIS La Fe, Plataforma de Farmacogenética, 46026 Valencia, Spain; Universidad de Valencia, Departamento de Farmacología, 46010 Valencia, Spain; Data Analysis Department, Instituto de Investigación Sanitaria-Fundación Jiménez Díaz University Hospital - Universidad Autónoma de Madrid (IIS-FJD, UAM), 28040 Madrid, Spain; Unidad de Infección Viral e Inmunidad, Centro Nacional de Microbiología (CNM), Instituto de Salud Carlos III (ISCIII), 28220 Madrid, Spain; Centro de Investigación Biomédica en Red de Enfermedades Infecciosas (CIBERINFEC), Instituto de Salud Carlos III, 28029 Madrid, Spain; Universidad de los Andes, Facultad de Ciencias, Bogotá 111711, Colombia; SIGEN Alianza Universidad de los Andes - Fundación Santa Fe de Bogotá, Bogotá 111711, Clombia; Hospital General de Segovia, Medicina Intensiva, 40002 Segovia, Spain; Department of Genetics & Genomics, Instituto de Investigación Sanitaria-Fundación Jiménez Díaz University Hospital - Universidad Autónoma de Madrid (IIS-FJD, UAM), 28040 Madrid, Spain; Centre for Biomedical Network Research on Rare Diseases (CIBERER), Instituto de Salud Carlos III, 28029 Madrid, Spain; Hospital Universitario 12 de Octubre, Department of Immunology, 28041 Madrid, Spain; Instituto de Investigación Sanitaria Hospital 12 de Octubre (imas12), Transplant Immunology and Immunodeficiencies Group, 28041 Madrid, Spain; Hospital General de Segovia, Medicina Intensiva, 40002 Segovia, Spain; Instituto de Biomedicina (IBIOMED), Universidad de León, 24071 León, Spain; Centre for Biomedical Network Research on Epidemiology and Public Health (CIBERESP), Instituto de Salud Carlos III, 28029 Madrid, Spain; Fundación Santa Fe de Bogota, Departamento Patologia y Laboratorios, Bogotá 111711, Colombia; SIGEN Alianza Universidad de los Andes - Fundación Santa Fe de Bogotá, Bogotá 111711, Clombia; Unidad de Genética y Genómica Islas Baleares, 07120 Islas Baleares, Spain; Hospital Universitario Son Espases, Unidad de Diagnóstico Molecular y Genética Clínica, 07120 Islas Baleares, Spain; Tecnológico de Monterrey, 64718 Monterrey, Mexico; Genomics of Complex Diseases Unit, Research Institute of Hospital de la Santa Creu i Sant Pau, IIB Sant Pau, 08041 Barcelona, Spain; Faculdade de Medicina, Universidade de Brasília, Brasilia 70910-900, Brazil; Programa de Pós-Graduação em Ciências Médicas, Universidade de Brasília, Brasilia 70910-900, Brazil; Programa de Pós-Graduação em Ciências da Saúde, Universidade de Brasília, Brasilia 70910-900, Brazil; Hospital El Bierzo, Unidad Cuidados Intensivos, 24404 León, Spain; Department of Genetics & Genomics, Instituto de Investigación Sanitaria-Fundación Jiménez Díaz University Hospital - Universidad Autónoma de Madrid (IIS-FJD, UAM), 28040 Madrid, Spain; Centre for Biomedical Network Research on Rare Diseases (CIBERER), Instituto de Salud Carlos III, 28029 Madrid, Spain; Hospital Universitario Mostoles, Medicina Interna, 28935 Madrid, Spain; Universidad Francisco de Vitoria, 28223 Madrid, Spain; Universidade Federal do Rio Grande do Norte, Departamento de Analises Clinicas e Toxicologicas, 59012-570 Natal, Brazil; Faculdade de Medicina, Universidade de Brasília, Brasilia 70910-900, Brazil; Programa de Pós-Graduação em Biologia Animal, Universidade de Brasília, Brasília 70910-900, Brazil; Programa de Pós-Graduação Profissional em Ensino de Biologia, Universidade de Brasília, Brasília 70910-900, Brazil; Programa de Pós-Graduação Profissional em Ensino de Biologia (UnB), Universidade de Brasília, Brasília 70910-900, Brazil; Programa de Pós-Graduação em Ciências Médicas, Universidade de Brasília, Brasília 70910-900, Brazil; Spanish National Cancer Research Center, CNIO Biobank, 28029 Madrid, Spain; Department of Child and Adolescent Psychiatry, Institute of Psychiatry and Mental Health, Hospital General Universitario Gregorio Marañón (IiSGM), 28007 Madrid, Spain; Centre for Biomedical Network Research on Mental Health (CIBERSAM), Instituto de Salud Carlos III, 28029 Madrid, Spain; School of Medicine, Universidad Complutense, 28040 Madrid, Spain; Hospital Universitario 12 de Octubre, Department of Immunology, 28041 Madrid, Spain; Instituto de Investigación Sanitaria Hospital 12 de Octubre (imas12), Transplant Immunology and Immunodeficiencies Group, 28041 Madrid, Spain; Universidad Complutense de Madrid, Department of Immunology, Ophthalmology and ENT, 28040 Madrid, Spain; Universidade Federal do Pará, Núcleo de Pesquisas em Oncologia, Belém, Pará 66075-110, Brazil; Infectious Diseases, Microbiota and Metabolism Unit, Center for Biomedical Research of La Rioja (CIBIR), 26006 Logroño, Spain; Inditex, 15141 A Coruña, Spain; Instituto Murciano de Investigación Biosanitaria (IMIB-Arrixaca), 30120 Murcia, Spain; GENYCA, 28220 Madrid, Spain; Cardiovascular Genetics Center, Institut d’Investigació Biomèdica Girona (IDIBGI), 17190 Girona, Spain; Centre for Biomedical Network Research on Cardiovascular Diseases (CIBERCV), Instituto de Salud Carlos III, 28029 Madrid, Spain; Instituto Mexicano del Seguro Social (IMSS), Centro Médico Nacional Siglo XXI, Unidad de Investigación Médica en Enfermedades Infecciosas y Parasitarias, Mexico City 02990, Mexico; Clinica Comfamiliar Risaralda, 660003 Pereira, Colombia; Bellvitge Biomedical Research Institute (IDIBELL), Neurometabolic Diseases Laboratory, 08908 L’Hospitalet de Llobregat, Spain; Centre for Biomedical Network Research on Rare Diseases (CIBERER), Instituto de Salud Carlos III, 28029 Madrid, Spain; Catalan Institution of Research and Advanced Studies (ICREA), 08010 Barcelona, Spain; Hospital Universitario de Getafe, Servicio de Genética, 28905 Madrid, Spain; Unidad de Infección Viral e Inmunidad, Centro Nacional de Microbiología (CNM), Instituto de Salud Carlos III (ISCIII), 28220 Madrid, Spain; Centro de Investigación Biomédica en Red de Enfermedades Infecciosas (CIBERINFEC), Instituto de Salud Carlos III, 28029 Madrid, Spain; Universidade Federal do Pará, Núcleo de Pesquisas em Oncologia, Belém, Pará 66075-110, Brazil; Hospital Ophir Loyola, Departamento de Ensino e Pesquisa, Belém, Pará 66063-240, Brazil; Unidad de Cuidados Intensivos, Hospital Clínico Universitario de Santiago (CHUS), Sistema Galego de Saúde (SERGAS), 15706 Santiago de Compostela, Spain; Instituto de Investigación Sanitaria de Santiago (IDIS), 15706 Santiago de Compostela, Spain; Department of Preventive Medicine and Public Health, School of Medicine, Universidad Autónoma de Madrid, 28049 Madrid, Spain; IdiPaz (Instituto de Investigación Sanitaria Hospital Universitario La Paz), 28046 Madrid, Spain; Centre for Biomedical Network Research on Epidemiology and Public Health (CIBERESP), Instituto de Salud Carlos III, 28029 Madrid, Spain; IMDEA-Food Institute, CEI UAM+CSIC, 28049 Madrid, Spain; Complejo Asistencial Universitario de León, 24071 León, Spain; Hospital Universitario Virgen de las Nieves, Servicio de Análisis Clínicos e Inmunología, 18014 Granada, Spain; Instituto de Investigación Biosanitaria de Granada (ibs GRANADA), 18012 Granada, Spain; Universidad de Granada, Departamento Bioquímica, Biología Molecular e Inmunología III, 18071 Granada, Spain; Hospital Infanta Elena, Allergy Unit, Valdemoro, 28342 Madrid, Spain; Instituto de Investigación Sanitaria-Fundación Jiménez Díaz University Hospital - Universidad Autónoma de Madrid (IIS-FJD, UAM), 28040 Madrid, Spain; Faculty of Medicine, Universidad Francisco de Vitoria, 28223 Madrid, Spain; Hospital Universitario Infanta Leonor, 28031 Madrid, Spain; Complutense University of Madrid, 28040 Madrid, Spain; Gregorio Marañón Health Research Institute (IiSGM), 28007 Madrid, Spain; Genomics of Complex Diseases Unit, Research Institute of Hospital de la Santa Creu i Sant Pau, IIB Sant Pau, 08041 Barcelona, Spain; Haemostasis and Thrombosis Unit, Hospital de la Santa Creu i Sant Pau, IIB Sant Pau, 08041 Barcelona, Spain; Hospital Clinico Universitario de Valladolid, Servicio de Anestesiologia y Reanimación, 47003 Valladolid, Spain; Universidad de Valladolid, Departamento de Cirugía, 47005 Valladolid, Spain; Hospital Clinico Universitario de Valladolid, Servicio de Hematologia y Hemoterapia, 47003 Valladolid, Spain; Data Analysis Department, Instituto de Investigación Sanitaria-Fundación Jiménez Díaz University Hospital - Universidad Autónoma de Madrid (IIS-FJD, UAM), 28040 Madrid, Spain; Hospital de Niños Ricardo Gutierrez, Buenos Aires C1425EFD CABA, Argentina; Fundación Universitaria de Ciencias de la Salud, 113827 Bogotá, Colombia; Spanish National Cancer Research Centre, Familial Cancer Clinical Unit, 28029 Madrid, Spain; University Hospital of Burgos, 09006 Burgos, Spain; Universidad Simón Bolívar, Facultad de Ciencias de la Salud, 080002 Barranquilla, Colombia; Centro para el Desarrollo de la Investigación Científica, 1255 Asunción, Paraguay; Institute for Molecular Medicine Finland (FIMM), 00014 Univerisity of Helsinki, Finland; McGill University, Department of Human Genetics, H3A 0G4 Montréal, Québec, Canada; Lady Davis Institute, Jewish General Hospital, McGill University, H3T 1E2 Montréal, Québec, Canada; Kyoto-McGill International Collaborative School in Genomic Medicine, Graduate School of Medicine, Kyoto University, 606-8501 Kyoto, Japan; Research Fellow, Japan Society for the Promotion of Science, 102-0083 Tokyo, Japan; University of Milano-Bicocca, 20126 Milano, Italy; Institute for Molecular Medicine Finland, Univerisity of Helsinki, 00014 Helsinki, Finland; Institute of Clinical Molecular Biology, Christian-Albrechts-University, 24118 Kiel, Germany; University Hospital Schleswig-Holstein, Campus Kiel, 24118 Kiel, Germany; Department of Epidemiology, Biostatistics and Occupational Health, McGill University, H3A 0G4 Montréal, Québec, Canada; Lady Davis Institute, Jewish General Hospital, McGill University, H3T 1E2 Montréal, Québec, Canada; Digestive Diseases Unit, Virgen del Rocio University Hospital, Institute of Biomedicine of Seville, University of Seville, 41103 Seville, Spain; Centre for Biomedical Network Research on Hepatic and Digestive Diseases (CIBEREHD), Instituto de Salud Carlos III, 28029 Madrid, Spain; Department of Liver and Gastrointestinal Diseases, Biodonostia Health Research Institute - Donostia University Hospital, University of the Basque Country (UPV/EHU), 20014 San Sebastian, Spain; Centre for Biomedical Network Research on Hepatic and Digestive Diseases (CIBEREHD), Instituto de Salud Carlos III, 28029 Madrid, Spain; Centre de Génétique Humaine, Hôpital Erasme, Université Libre de Bruxelles, 1070 Brussels, Belgium; Liver Unit, Department of Internal Medicine, Hospital Universitari Vall d’Hebron, Vall d’Hebron Barcelona Hospital Campus, 08035 Barcelona, Spain; Universitat Autònoma de Barcelona, Departament de Medicina, Bellatera, 08193 Barcelona, Spain; Vall d’Hebron Institut de Recerca (VHIR), Liver Diseases, 08035 Barcelona, Spain; Novo Nordisk Foundation Center for Protein Research, Disease Systems Biology, Faculty of Health and Medical Sciences, University of Copenhagen, DK-2200 Copenhagen, Denmark; Institute of Clinical Molecular Biology, Christian-Albrechts-University, 24118 Kiel, Germany; GENYO, Centre for Genomics and Oncological Research: Pfizer, University of Granada, Andalusian Regional Government, 18016 Granada, Spain; Institute of Human Genetics, University Hospital Bonn, Medical Faculty University of Bonn, 53127 Bonn, Germany; Genetica Medica, Azienda Ospedaliero-Universitaria Senese, 53100 Siena, Italy; Centre for Biomedical Network Research on Hepatic and Digestive Diseases (CIBEREHD), Instituto de Salud Carlos III, 28029 Madrid, Spain; Liver Unit, Department of Internal Medicine, Hospital Universitari Vall d’Hebron, Vall d’Hebron Barcelona Hospital Campus, 08035 Barcelona, Spain; Universitat Autònoma de Barcelona, Departament de Medicina, Bellatera, 08193 Barcelona, Spain; University of Siena, Medical Genetics, 53100 Siena, Italy; Azienda Ospedaliero-Universitaria Senese, Genetica Medica, 53100 Siena, Italy; Med Biotech Hub and Competence Center, Department of Medical Biotechnologies, University of Siena, 53100 Siena, Italy; Technical University of Munich, School of Medicine, University Hospital rechts der Isar, Department of Internal Medicine II, 80333 Munich, Germany; Department of Transfusion Medicine and Hematology, Fondazione IRCCS Ca’ Granda Ospedale Maggiore Policlinico, Università degli Studi di Milano, 20126 Milano, Italy; Mucosal Immunology Lab, Unidad de Excelencia del Instituto de Biomedicina y Genética Molecular (IBGM, Universidad de Valladolid-CSIC), 47005 Valladolid, Spain; Centre for Biomedical Network Research on Hepatic and Digestive Diseases (CIBEREHD), Instituto de Salud Carlos III, 28029 Madrid, Spain; Hospital Universitario Clinico San Cecilio, 18016 Granada, Spain; Instituto de Investigación Ibs, Granada, 18012 Granada, Spain; University of Liege. GIGA-Insitute, B- 4000 Liege, Belgium; Liege University Hospital (CHU of Liege), B- 4000 Liege, Belgium; Biomedical Research Institute Sant Pau (IIB Sant Pau), Stroke Pharmacogenomics and Genetics Group, 08041 Barcelona, Spain; Oslo University Hospital, Department of Microbiology, 0424 Oslo, Norway; Institute of Clinical Medicine, University of Oslo, 0424 Oslo, Norway; Department of Liver and Gastrointestinal Diseases, Biodonostia Health Research Institute - Donostia University Hospital, University of the Basque Country (UPV/EHU), Ikerbasque, 20014 San Sebastian, Spain; Centre for Biomedical Network Research on Hepatic and Digestive Diseases (CIBEREHD), Instituto de Salud Carlos III, 28029 Madrid, Spain; Department of Surgical Sciences, Anaesthesiology and Intensive Care Medicine, Uppsala University, 751 05 Uppsala, Sweden; Humanitas University, Department of Biomedical Sciences, 20089 Milan, Italy; IRCCS Humanitas Research Hospital, Rozzano, 20089 Milan, Italy; Humanitas University, Department of Biomedical Sciences, 20089 Milan, Italy; IRCCS Humanitas Research Hospital, Rozzano, 20089 Milan, Italy; Heart Institute (InCor)/University of Sao Paulo Medical School, 05508-070 Sao Paulo, Brazil; Digestive Diseases Unit, Virgen del Rocio University Hospital, Institute of Biomedicine of Seville, University of Seville, 41103 Seville, Spain; Centre for Biomedical Network Research on Hepatic and Digestive Diseases (CIBEREHD), Instituto de Salud Carlos III, 28029 Madrid, Spain; Osakidetza Basque Health Service, Donostialdea Integrated Health Organisation, Clinical Biochemistry Department, 20006 San Sebastian, Spain; Norwegian PSC Research Center and Section of Gastroenterology, Dept Transplantation Medicine, Oslo University Hospital, 0424 Oslo, Norway; Institute of Clinical Medicine, University of Oslo, 0424 Oslo, Norway; Research Institute of Internal Medicine, Oslo University Hospital, 0424 Oslo, Norway; Fonds de la Recherche Scientifique (FNRS), B – 1000 Brussels; Centre de Génétique Humaine, Hôpital Erasme, Université Libre de Bruxelles, 1070 Brussels, Belgium; University of Siena, Medical Genetics, 53100 Siena, Italy; Azienda Ospedaliero-Universitaria Senese, Genetica Medica, 53100 Siena, Italy; Med Biotech Hub and Competence Center, Department of Medical Biotechnologies, University of Siena, 53100 Siena, Italy; Institute for Biomedical Research of Barcelona (IIBB), National Spanish Research Council (CSIC), 08028 Barcelona, Spain; Institut d’Investigacions Biomediques August Pi i Sunyer (IDIBAPS), 08036 Barcelona, Spain; Institute of Human Genetics, University Hospital Bonn, Medical Faculty University of Bonn, 53127 Bonn, Germany; Centre for Biomedical Network Research on Hepatic and Digestive Diseases (CIBEREHD), Instituto de Salud Carlos III, 28029 Madrid, Spain; Liver Unit, Department of Internal Medicine, Hospital Universitari Vall d’Hebron, Vall d’Hebron Barcelona Hospital Campus, 08035 Barcelona, Spain; Universitat Autònoma de Barcelona, Departament de Medicina, Bellatera, 08193 Barcelona, Spain; University of Liege. GIGA-Insitute, B- 4000 Liege, Belgium; GENYO, Centre for Genomics and Oncological Research: Pfizer, University of Granada, Andalusian Regional Government, 18016 Granada, Spain; Institute for Environmental Medicine, Karolinska Institutet, 171 65 Solna, Sweden; Institute of Virology, Technical University Munich/Helmholtz Zentrum München, D-85764 Munich, Germany; Institute of Psychiatric Phenomics and Genomics, University Hospital, LMU Munich University, 80539 Munich, Germany; Department of Psychiatry, University Hospital, LMU Munich University, 80539 Munich, Germany; Institute of Clinical Molecular Biology, Christian-Albrechts-University, 24118 Kiel, Germany; University Hospital Schleswig-Holstein, Campus Kiel, 24118 Kiel, Germany; Norwegian PSC Research Center and Section of Gastroenterology, Dept Transplantation Medicine, Oslo University Hospital, 0424 Oslo, Norway; Institute of Clinical Medicine, University of Oslo, 0424 Oslo, Norway; Research Institute of Internal Medicine, Oslo University Hospital, 0318 Oslo, Norway; Università degli Studi di Milano, Department of Pathopgysiology and Transplantation, 20126 Milano, Italy; Department of Transfusion Medicine and Hematology, Precision Medicine, Fondazione IRCCS Ca’ Granda Ospedale Maggiore Policlinico, 20122 Milano, Italy; Karolinska Institutet, Department of Neuroscience, 171 77 Stockholm, Sweden; Max-Planck Institute for Evolutionary Anthropology, 04103 Leipzig, Germany; Department of Human Genetics, Epidemiology, Biostatistics and Occupational Health, McGill University, H3A 0G4 Montréal, Québec, Canada; Lady Davis Institute, Jewish General Hospital, McGill University, H3T 1E2 Montréal, Québec, Canada; King’s College London, Department of Twin Research, London, WC2R 2LS, United Kingdom; Institute for Molecular Medicine Finland, Univerisity of Helsinki, 00014 Helsinki, Finland; Massachusetts General Hospital, Harvard Medical School, Boston, MA 02114, USA; Research Center and Memory clinic, ACE Alzheimer Center Barcelona, Universitat Internacional de Catalunya, 08028 Barcelona, Spain; Centre for Biomedical Network Research on Neurodegenerative Diseases (CIBERNED), Instituto de Salud Carlos III, 28029 Madrid, Spain; Research Center and Memory clinic, ACE Alzheimer Center Barcelona, Universitat Internacional de Catalunya, 08028 Barcelona, Spain; Centre for Biomedical Network Research on Neurodegenerative Diseases (CIBERNED), Instituto de Salud Carlos III, 28029 Madrid, Spain; Research Center and Memory clinic, ACE Alzheimer Center Barcelona, Universitat Internacional de Catalunya, 08028 Barcelona, Spain; Centre for Biomedical Network Research on Neurodegenerative Diseases (CIBERNED), Instituto de Salud Carlos III, 28029 Madrid, Spain; CIEN Foundation/Queen Sofia Foundation Alzheimer Center, 28031 Madrid, Spain; Hospital Universitario de Valme, Unidad Clínica de Enfermedades Infecciosas y Microbiología, 41014 Sevilla, Spain; Instituto Murciano de Investigación Biosanitaria (IMIB-Arrixaca), 30120 Murcia, Spain; Sección Genética Médica - Servicio de Pediatría, Hospital Clínico Universitario Virgen de la Arrixaca, Servicio Murciano de Salud, 30120 Murcia, Spain; Departamento Cirugía, Pediatría, Obstetricia y Ginecología, Facultad de Medicina, Universidad de Murcia (UMU), 30100 Murcia, Spain; Grupo Clínico Vinculado, Centre for Biomedical Network Research on Rare Diseases (CIBERER), Instituto de Salud Carlos III, 28029 Madrid, Spain; Department of Genetics & Genomics, Instituto de Investigación Sanitaria-Fundación Jiménez Díaz University Hospital - Universidad Autónoma de Madrid (IIS-FJD, UAM), 28040 Madrid, Spain; Centre for Biomedical Network Research on Rare Diseases (CIBERER), Instituto de Salud Carlos III, 28029 Madrid, Spain; Spanish National Cancer Research Centre, Human Genotyping-CEGEN Unit, 28029 Madrid, Spain; IDIVAL, 39008 Cantabria, Spain; Universidad de Cantabria, 39008 Cantabria, Spain; Hospital U M Valdecilla, 39008 Cantabria, Spain; Tecnologico de Monterrey, Escuela de Medicina y Ciencias de la Salud, 64718 Monterrey, Mexico; Genomics Division, Instituto Tecnológico y de Energías Renovables, 38600 Santa Cruz de Tenerife, Spain; Research Unit, Hospital Universitario N.S. de Candelaria, 38010 Santa Cruz de Tenerife, Spain; Centre for Biomedical Network Research on Respiratory Diseases (CIBERES), Instituto de Salud Carlos III, 28029 Madrid, Spain; Facultad de Ciencias de la Salud, Universidad Fernando Pessoa Canarias, 35450 Las Palmas de Gran Canaria, Spain; Centre for Biomedical Network Research on Rare Diseases (CIBERER), Instituto de Salud Carlos III, 28029 Madrid, Spain; Instituto de Genética Médica y Molecular (INGEMM), Hospital Universitario La Paz-IDIPAZ, 28046 Madrid, Spain; ERN-ITHACA-European Reference Network; Centro Nacional de Genotipado (CEGEN), Universidade de Santiago de Compostela, 15706 Santiago de Compostela, Spain; Centre for Biomedical Network Research on Rare Diseases (CIBERER), Instituto de Salud Carlos III, 28029 Madrid, Spain; Instituto de Investigación Sanitaria de Santiago (IDIS), 15706 Santiago de Compostela, Spain; Centro Singular de Investigación en Medicina Molecular y Enfermedades Crónicas (CIMUS), Universidade de Santiago de Compostela, 15782 Santiago de Compostela, Spain; Fundación Pública Galega de Medicina Xenómica, Sistema Galego de Saúde (SERGAS), 15706 Santiago de Compostela, Spain

## Abstract

Here, we describe the results of a genome-wide study conducted in 11 939 coronavirus disease 2019 (COVID-19) positive cases with an extensive clinical information that were recruited from 34 hospitals across Spain (SCOURGE consortium). In sex-disaggregated genome-wide association studies for COVID-19 hospitalization, genome-wide significance (*P* < 5 × 10^−8^) was crossed for variants in 3p21.31 and 21q22.11 loci only among males (*P =* 1.3 × 10^−22^ and *P =* 8.1 × 10^−12^, respectively), and for variants in 9q21.32 near *TLE1* only among females (*P =* 4.4 × 10^−8^). In a second phase, results were combined with an independent Spanish cohort (1598 COVID-19 cases and 1068 population controls), revealing in the overall analysis two novel risk loci in 9p13.3 and 19q13.12, with fine-mapping prioritized variants functionally associated with *AQP3* (*P =* 2.7 × 10^−8^) and *ARHGAP33* (*P =* 1.3 × 10^−8^), respectively. The meta-analysis of both phases with four European studies stratified by sex from the Host Genetics Initiative (HGI) confirmed the association of the 3p21.31 and 21q22.11 loci predominantly in males and replicated a recently reported variant in 11p13 (*ELF5, P* = 4.1 × 10^−8^). Six of the COVID-19 HGI discovered loci were replicated and an HGI-based genetic risk score predicted the severity strata in SCOURGE. We also found more SNP-heritability and larger heritability differences by age (<60 or ≥60 years) among males than among females. Parallel genome-wide screening of inbreeding depression in SCOURGE also showed an effect of homozygosity in COVID-19 hospitalization and severity and this effect was stronger among older males. In summary, new candidate genes for COVID-19 severity and evidence supporting genetic disparities among sexes are provided.

## Introduction

Coronavirus disease 2019 (COVID-19)—caused by the severe acute respiratory syndrome coronavirus 2 (SARS-CoV-2)—develops with wide clinical variability, ranging from asymptomatic infection to a life-threatening condition (1). Advanced age and the presence of comorbidities are well-known major risk factors of COVID-19 severity (2,3). In addition, male sex is another risk factor associated with COVID-19 severity, regardless of comorbidities (4).

International genetic studies based on genome-wide association studies (GWAS) and/or comparative genome sequencing analyses have been conducted to identify genetic variants associated with COVID-19 severity (5,6). These studies have revealed the role of genes of the type-I interferon (IFN) signaling pathway as key players underlying disease severity (7–9). Besides, they have also identified other potential loci previously linked to lung function, respiratory diseases and autoimmunity (9). Regarding COVID-19 severity in males, sex-disaggregated genetic analyses have received limited attention despite the importance of sex disparities in clinical severity (10). Early studies suggested immune deficits presumably because of pre-existing neutralizing autoantibodies against type-I IFN in older male patients (11).

The effects of autozygosity, measured as the change of the mean value of a complex trait because of inbreeding, have been useful to identify alternative genetic risk explanations and effects that traditionally are not captured by GWAS (12). By analyzing the contribution of the inbreeding depression (ID) through the lens of the runs of homozygosity (ROH: genomic tracts where homozygous markers occur in an uninterrupted sequence), it is possible to assess the importance of directional dominance or overdominance in the genetic architecture of complex traits (13). Even though this is a relatively modern approach, different studies have shown the importance of homozygosity in a large range of complex phenotypes, including anthropometric, cardiometabolic and mental traits (14–16).

Through diverse nested sub-studies, the Spanish Coalition to Unlock Research on Host Genetics on COVID-19 (SCOURGE) consortium was launched in May 2020 aiming to find biomarkers of evolution and prognosis that can have an immediate impact on clinical management and therapeutic decisions in SARS-CoV-2 infections. This consortium has recruited patients from hospitals across Spain and Latin America in close collaboration with the STOP-Coronavirus initiative (https://www.scourge-covid.org). Here, we describe the results of the first SCOURGE genome-wide studies of COVID-19 conducted in patients recruited in Spain. This dataset has not been used in any previous GWAS of COVID-19 that has been published to date. To the best of our knowledge, this is the first time that the impact of homozygosity is considered in COVID-19 studies, serving as a complement to the traditional GWAS to assess both the additive and dominant components of the genetic architecture of COVID-19 severity. Likewise, the ID analysis could also help to explain the strong effect of age in COVID-19 severity.

## Results

### Discovery phase

In the SCOURGE study, 11 939 COVID-19 positive cases were recruited from 34 centers (Supplementary Material, Table S1) between March and December 2020. All diagnosed cases were classified in a five-level severity scale (Table 1). Two untested Spanish sample collections were used as general population controls in some analyses: 3437 samples from the Spanish DNA biobank (https://www.bancoadn.org) and 2506 samples from the GR@CE consortium (17). The discovery phase samples were genotyped with the Axiom Spain Biobank Array (Thermo Fisher Scientific). Details of quality control (QC), ancestry inference and imputation are shown in the Materials and Methods section. Individuals with inferred European ancestry were used for association testing. After post-imputation filtering, 15 045 individuals (9371 COVID-19 positive cases and 5674 population controls) and 8 933 154 genetic markers remained in the SCOURGE European study (discovery). Clinical and demographic characteristics of European patients from SCOURGE included in the analysis are shown in Table 2. Population controls were 46.3% females with a mean age of 55.5 years (standard deviation, SD = 16.2) and 53.7% males, with a mean age of 51 years (SD = 13.04).

**Table 1:** Five-level severity scale used to classify SCOURGE patients

Level	Clinical findings
Severity 0 (asymptomatic)	Asymptomatic
Severity 1 (mild)	With symptoms, but without pulmonary infiltrates or need of oxygen therapy
Severity 2 (moderate)	With pulmonary infiltrates affecting <50% of the lungs or need of supplemental oxygen therapy
Severity 3 (severe)	Hospitalized with any of the following criteria: PaO_2_ < 65 mmHg or SaO_2_ < 90% PaO_2_/FiO_2_ < 300 SaO_2_/FiO_2_ < 440 Dyspnea Respiratory frequency ≥ 22 bpm Infiltrates affecting > 50% of the lungs
Severity 4 (critical)	Admission to the ICU or need of mechanical ventilation (invasive or non-invasive)

*Note:* PaO_2_, partial pressure of oxygen in arterial blood; SaO_2_, saturation of oxygen in arterial blood; FiO_2_, fraction of inspired oxygen.

**Table 2:** Baseline characteristics of European patients from SCOURGE included in the analysis

Variable	Global *N* = 9371	Males *N* = 4343	Females *N* = 5028
Age—mean years (SD)	62.6 (17.9)	64.3 (16.3)	61.1 (19.1)
Severity—*N* (%)			
0—asymptomatic	582 (6.6)	161 (3.9)	421 (8.9)
1—mild	2689 (30.3)	748 (18.2)	1941 (40.8)
2—intermediate	2099 (23.6)	1093 (26.5)	1006 (21.1)
3—severe	2379 (26.8)	1300 (31.6)	1079 (22.7)
4—critical illness	1128 (12.7)	817 (19.8)	311 (6.5)
Hospitalization—*N* (%)	5966 (63.8)	3436 (79.3)	2530 (50.5)
Severe COVID-19—*N* (%)	3507 (39.2)	2117 (51.2)	1390 (28.9)
Critical illness—*N* (%)	1128 (12.6)	817 (19.8)	311 (6.5)
Comorbidities—*N* (%)			
Vascular/endocrinological	4099 (43.7)	2207 (50.8)	1892 (37.6)
Cardiac	1057 (11.3)	634 (14.6)	423 (8.4)
Nervous	773 (8.3)	341 (7.9)	432 (8.6)
Digestive	264 (2.8)	153 (3.5)	111 (2.2)
Onco-hematological	647 (6.9)	411 (9.5)	236 (4.7)
Respiratory	905 (9.7)	565 (13.0)	340 (6.8)

The discovery phase of the GWAS was carried out with infection susceptibility and three severity outcomes (hospitalization, severe illness and critical illness), which were tested using three different control definitions (see Supplementary Material, Table S2).

- A1 analysis: COVID-19 positive not satisfying the case condition and control samples from the general population (COVID-19 untested).
- A2 analysis: control samples from the general population.
- C analysis: COVID-19 positive not satisfying the case condition.

The GWAS was carried on by fitting logistic mixed regression models adjusting for age, sex and the first 10 principal components (PCs; see Materials and Methods). Summary statistics can be accessed from https://github.com/CIBERER/Scourge-COVID19. The SCOURGE Board of Directors has agreed to aggregate the GWAS summaries with those from the COVID-19 Host Genetics Initiative (HGI) in the data freeze 7 that has not been used for any published article to date. Supplementary Material, Table S3 shows the independent significant associated loci for hospitalization, severity, critical illness and infection susceptibility risk, for global and sex-stratified analysis in the SCOURGE dataset. However, considering the overlap between the findings for these analyses, only the main results for the A1 analysis are presented.

**Figure 1:**
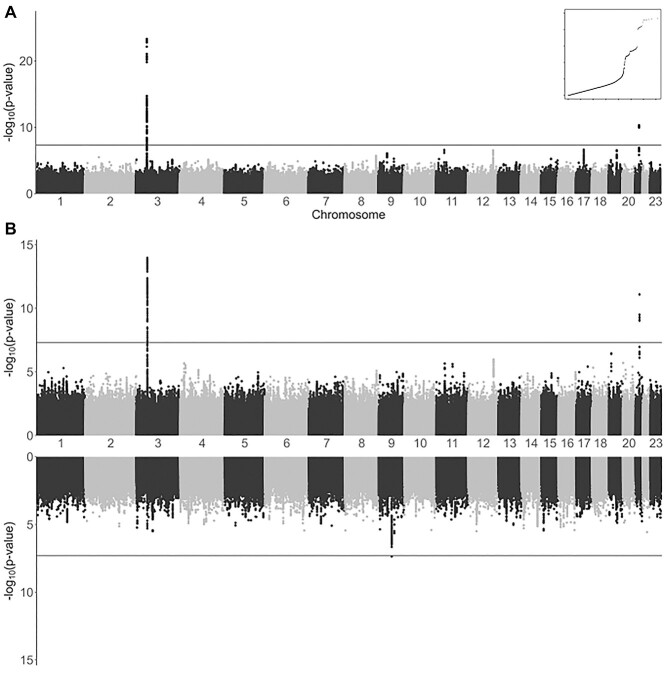
Association results of SCOURGE for global and sex-disaggregated A1 hospitalization analysis. (**A**) Manhattan plot of results from global analysis. A quantile–quantile plot of the global analysis is also shown as an inset. (**B**) Miami plot of results from sex-disaggregated analyses (top: males and bottom: females).

**Figure 2:**
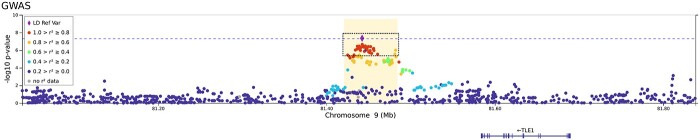
Regional plot of a novel association at 9q21.32 found among females from the SCOURGE study. The *x* axis reflects the chromosomal position, and the *y* axis the −log(*P*-value). The sentinel variant is indicated by a diamond and all other variants are colour coded by their degree of LD with the sentinel variant in Europeans. Credible set for this signal is shown within a dashed square. The horizontal dotted blue line corresponds to the threshold for genome-wide significance (*P* = 5 × 10^−8^).

All analyses support the association of two known loci, i.e. 3p21.31 and 21q22.11. However, other suggestive associations were also found (Fig. 1 and Supplementary Material, Fig. S1). Strikingly, the leading signals found in the global (sex-aggregated) analysis were genome-wide significant in the analyses among males but not among females. Association in 3p21.31 was also found in the C analyses (rs10490770, *P* = 3.8 × 10^−12^) and once again, association was genome-wide significant only among males (males: *P* = 3.9 × 10^−9^, females: *P* = 4.6 × 10^−5^). However, the leading variant of 9q21.32 (near *TLE1* gene) reached genome-wide significance among females only (similarly, in the C analysis for females, rs140152223, *P* = 2.11 × 10^−6^). Several variants (rs17763742 near *LZTFL1*, rs2834164 in *IFNAR2* and rs1826292621 near *TLE1*) showed a significant difference in effect sizes (SNP*sex interaction *P* < 0.0031, adjusted probability for 16 comparisons) linked not only to hospitalization, but also to critical illness and infection risk. The A2 and C analyses did not reveal any additional significant loci (Supplementary Material, Fig. S2). Although fine-mapping studies in 3p21.31 and 21q22.11 have led to gene and variant prioritization within these loci (Supplementary Material, Fig. S3), a Bayesian fine-mapping on the 9q21.32 did not allow to prioritize variants by their role as expression quantitative trait loci (eQTLs) or anticipate the function (Fig. 2). To assess if a higher prevalence of comorbidities in males may underlie these differential findings, we performed an additional C analysis in which the presence of comorbidities was tested for association within hospitalized patients. No significant association was found in either males or females (Supplementary Material, Fig. S4). Further explorations of the genetic associations with comorbidities are presented in the Supplementary Note.

This GWAS phase was also performed disaggregated by age (<60/≥60 years old), and by age and sex simultaneously. Differences in effect sizes between both age groups were tested for the SNPs shown in the Supplementary Material, Table S3, in global and sex-specific analysis (Supplementary Material, Table S4). Significant findings were only found in the subgroup of males with <60 years old. This was also found in the C analysis for hospitalization where association in 3p21.31 was significant only in males <60 years old (*P* = 3.32 × 10^−9^). Differences in effect size (significant age-interaction) were significant at 3p21.31 for severity and critical illness, and suggestive in hospitalization.

### Lookup of previously found COVID-19 host risk factors in the SCOURGE study

**Figure 3:**
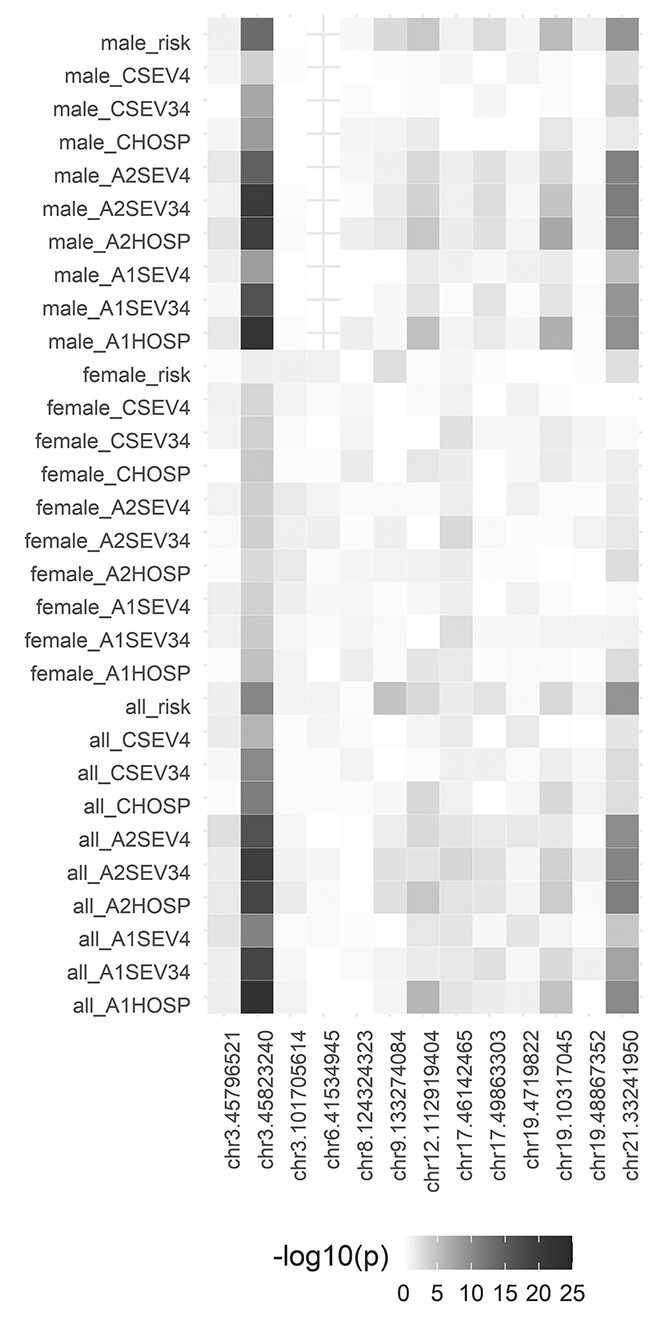
Lookup of previously found COVID-19 host risk factors in the SCOURGE study. Heatmap illustrating the results in all analyses performed in this study (rows) for the 13 leading variants in the COVID-19 HGI study (columns). Each box illustrates the top associated variant within the focal region. The color (gray to dark red) indicates the strength (significance level) of the association in SCOURGE. Note: In three cases (chr12: 112919388, chr19: 4719431 and chr21: 33242905), the imputed variants did not pass QC filters in SCOURGE and they were replaced by the nearest QC-ed imputed variant (chr12:112919404, chr19:4719822 and chr21:33241950, respectively).

Known significant loci for COVID-19 severity in 3p21.31 (near *SLC6A20* and *LZTFL1*) and 21q22.11 (in *IFNAR2*) were clearly replicated at genome-wide significance in this study, specifically for risk of infection, hospitalization and severity. Three other loci, in 9q34.2 (in *ABO*), 12q24.13 (in *OAS1*) and 19p13.2 (near *RAVER1* and *TYK2*), did not reach the genome-wide significance threshold but they were significant after correcting for the 390 tests performed in a lookup (13 SNPs and 30 analyses, significance threshold *P* < 1.3 × 10^−4^). In agreement with previous results, *ABO* was mainly associated with the risk of infection. However, other loci as those in 3q12.3 (near *RPL24*) and 19p13.3 (near *DPP9*), previously found associated with COVID-19 severity, were not replicated in the SCOURGE Europeans. The complete list of results for the list of COVID-19 HGI significant loci (9) is shown in Figure 3 and in the Supplementary Material, Table S5. Figure 3 also reinforces the clear sex differences found in this study.

### Genetic risk score and the COVID-19 severity scale

We developed a genetic risk score (GRS) combining the 13 leading variants associated with infection risk, hospitalization or severity in the meta-analysis performed by the COVID-19 HGI (9). This GRS predicted the severity scale in SCOURGE but supporting the differentiation in three classes: (i) controls/asymptomatic/mild cases; (ii) moderate and severe cases and (iii) critical cases. (Supplementary Material, Fig. S5). Simultaneously disaggregating by age (<60/≥60 years old) and sex, we identify the three severity classes in the subgroup of males with <60 years old, supporting the importance of this group in the overall findings (Supplementary Material, Fig. S5). Details of this analysis can be found in Supplementary Note.

### Second study phase and meta-analysis with the discovery

Results for hospitalization risk were meta-analysed with a second Spanish cohort, the CNIO study (see Materials and Methods). This study was filtered following the same QC and imputation procedures. The final dataset of the CNIO study included 2446 European individuals (1378 COVID-19 positive cases and 1068 population controls) and 8 895 721 markers.

**Figure 4:**
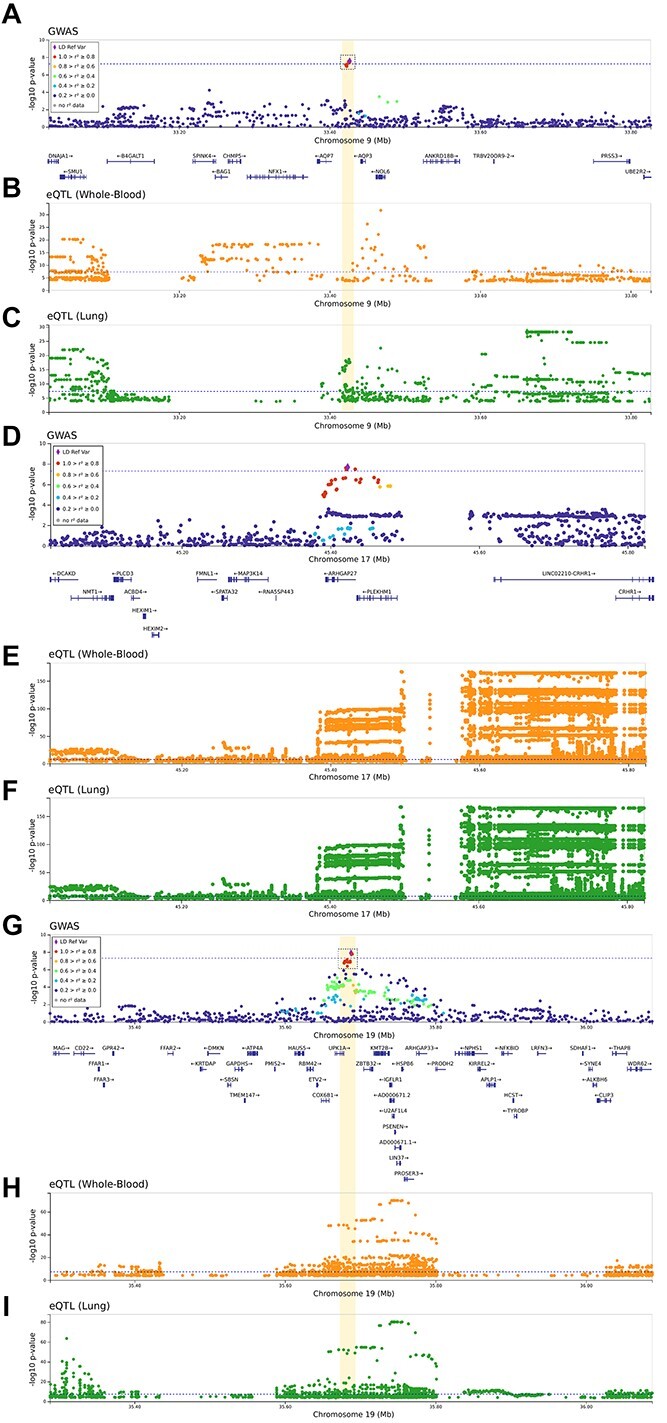
Regional plots of novel association signals found from the meta-analysis between the SCOURGE and CNIO studies. Regional plots of novel association signals found in 9p13.3 (**A**–**C**), 17q21.31 (**D**–**F**) and 19q13.12 (**G**–**I**). The *x* axes reflect the chromosomal position, and the *y* axes the −log(*P*-value) of the SCOURGE-CNIO meta-analysis. On A, D and G the sentinel variant is indicated by a diamond and all other variants are color coded by their degree of LD with the sentinel variant in Europeans. Whenever a concise set of variants was prioritized, a credible set is shown within a dashed square. The horizontal dotted blue line corresponds to the threshold for genome-wide significance (*P* = 5 × 10^−8^). In the rest of panels, the *x* axes reflect the chromosomal position, and the *y* axes the −log(*P*-value) resulting from the eQTL analyses in whole blood (B, E and H) and in the lung (C, F and I) whenever a significant finding is available from GTEx v8.

Table 3 shows the results that were genome-wide significant either in global or sex-stratified meta-analysis with SCOURGE. Besides the widely replicated loci at 3p21.31 and 21q22.11, three additional signals were found: chr9:33426577:A:G (intergenic to *AQP7* and *AQP3*), chr17:45422978:G:C (intronic to *ARHGAP27*) and chr19:35687796:G:A (intergenic to *UPK1A* and *ZBTB32*). Bayesian fine-mapping around chr17:45422978:G:C failed to prioritize a credible set of variants, hindering functional links of the locus. Functional assessments of the prioritized variants by the Bayesian fine-mapping analysis in the other two regions supported that they were eQTLs of the *AQP3* and *ARGAP33* genes, including whole blood and lung tissues (Fig. 4).

**Table 3:** Genome-wide significant variants in global or sex-stratified meta-analysis between the SCOURGE and CNIO studies

SNP	chr:position			Meta-ALL			Meta-males			Meta-females			Nearest gene
		EA	NEA	beta	SE	*P*-value	beta	SE	*P*-value	beta	SE	*P*-value	
rs115679256	3:45587795	G	A	0.43	0.08	1.1E−08	0.48	0.10	2.3E−06	0.40	0.11	2.9E−04	*LIMD1*
rs17763742	3:45805277	A	G	0.60	0.05	4.1E−29	0.74	0.07	3.3E−25	0.43	0.08	4.5E−08	*LZTFL1*
rs35477280	3:45932600	G	A	0.39	0.05	1.4E−17	0.48	0.06	6.3E−15	0.28	0.07	1.6E−05	*FYCO1*
rs4443214	3:46136372	T	C	0.25	0.04	9.0E−09	0.26	0.06	1.4E−05	0.26	0.06	4.4E−05	*XCR1*
rs115102354	3:46180545	A	G	0.41	0.07	1.6E−08	0.52	0.10	2.1E−07	0.32	0.10	2.0E−03	*CCR3*
rs10813976	9:33426577	A	G	0.18	0.03	2.7E−08	0.16	0.04	2.5E−04	0.19	0.05	3.5E−05	*AQP3*
rs1230082	17:45422978	C	G	0.16	0.03	2.1E−08	0.17	0.04	2.8E−05	−0.15	0.04	2.5E−04	*ARHGAP27*
rs77127536	19:35687796	G	A	−0.22	0.04	1.3E−08	−0.25	0.05	2.1E−06	−0.19	0.05	4.3E−04	*UPK1A/ZTBT32*
rs17860169	21:33240996	A	G	0.19	0.03	2.3E−11	0.27	0.04	1.4E−11	0.12	0.04	3.7E−03	*IFNAR2*

*Note:* Representative SNPs were selected using the clump function of PLINK 1.9 (clumping parameters *r*^2^ = 0.5, *P*val = 5 × 10^−8^ and *P*val_2_ = 0.05). EA, effect allele; NEA, non-effect allele; beta, effect coefficient; SE, standard error.

These variants were also associated with the three severity groups previously outlined in SCOURGE by the GRS under a multinomial model (Supplementary Material, Table S6).

### Meta-analysis in independent European studies

Hospitalization risk was meta-analysed with other European studies combining both Spanish cohorts (SCOURGE and CNIO) with other four sex-disaggregated studies from the COVID-19 HGI consortium, namely: BelCOVID, GenCOVID, Hostage-Spain and Hostage-Italy (Table 4). Once again, the most outstanding significant loci were found at 3p21.31 and 21q22.11 (in global and male-specific analyses), and three additional loci reached genome-wide significance in the meta-analysis of males: chr12:11292383:A:G (in *OAS1*), chr19:35687796:G:A (intergenic to *UPK1A* and *ZBTB32*) and chr11:34482745:G:A (in *ELF5*). The 3p21.31 variants reached genome-wide significance in females, although at significantly lower level than in males despite the similar sample sizes (*Z* = 3.33, *P =* 5 × 10^−4^).

**Table 4:** Results of European meta-analysis for hospitalization risk

				Meta-all			Meta-males			Meta-females			
SNP	chr:position	EA	NEA	beta	SE	*P*-value	beta	SE	*P*-value	beta	SE	*P*-value	Nearest gene
rs115679256	3:45587795	G	A	0.37	0.06	1.3E−08	0.41	0.08	5.6E−07	0.36	0.09	1.6E−04	*LIMD1*
rs13078854	3:45820440	G	A	0.53	0.04	6.7E−34	0.64	0.05	2.7E−33	0.38	0.06	1.0E−09	*LZTFL1*
rs41289622	3:45973053	T	G	0.36	0.04	3.6E−21	0.44	0.05	3.4E−20	0.27	0.05	7.2E−07	*FYCO1*
rs115102354	3:46180545	A	G	0.40	0.06	8.9E−12	0.48	0.07	6.8E−11	0.26	0.08	1.8E−03	*XCR1*
rs61882275	11:34482745	G	A	−0.12	0.02	1.0E−06	−0.17	0.03	4.1E−08	−0.08	0.03	1.3E−02	*ELF5*
rs4767028	12:112921383	A	G	−0.16	0.02	1.6E−10	−0.19	0.03	2.5E−09	−0.11	0.04	8.7E−04	*OAS1*
rs12609134	19:35687796	G	A	−0.19	0.03	2.3E−08	−0.22	0.04	9.5E−08	−0.13	0.05	6.0E−03	*UPK1A/ZBTB32*
rs17860169	21:33240996	A	G	0.18	0.03	3.9E−12	0.21	0.03	1.6E−10	0.15	0.04	2.9E−05	*IFNAR2*

*Note:* Summary statistics of both phases (SCOURGE and CNIO) were meta-analysed with four additional sex-disaggregated European studies from the COVID-19 HGI consortium. EA, effect allele; NEA, non-effect allele; beta, effect coefficient; SE, standard error.

Significance of two interesting loci revealed in the Spanish studies was reduced in the meta-analysis with other European studies, although still showed suggestive associations: that of 9q21.32 near *TLE1* previously found only in females (female meta-analysis *P =* 5.4 × 10^−7^), and that of 9p13.3 near *AQP3* (global meta-analysis, *P =* 1.23 × 10^−7^).

### Heritability of COVID-19 hospitalization

**Figure 5:**
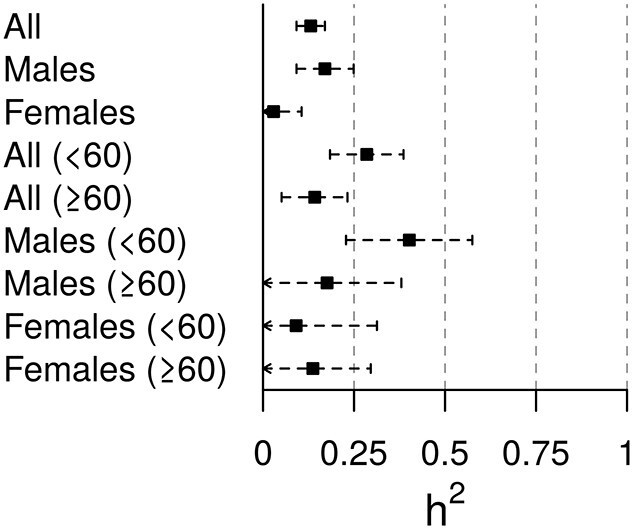
Forest plot of the SNP-heritability estimates for the COVID-19 hospitalization risk analysis on the liability scale.

In the hospitalization risk analysis, we found that common variants (minor allele frequency, MAF > 1%) explain 27.1% (95% confidence interval, CI: 19.0–35.3%) of heritability on the observed scale (corresponding to 13.1% [95%CI: 9.2–17.0%] on the liability scale, assuming a prevalence of 0.5%; Fig. 5). We observed less heritability among females than males (2.9% [95%CI: 0.00–10.6%] in females and 17.0% [95%CI: 9.2–24.9%] in males on the liability scale). In agreement with observations suggesting an accumulation of non-genetic risk factors with age, especially among males (11,18), we observed larger heritability differences by age groups among males (40.2% [95%CI: 22.8–57.5%] in <60 years versus 17.6% [95%CI: 0.00–38.0%] in ≥60 years on the liability scale) than among females (9.1% [0.00–31.3%] in <60 years versus 13.7% [0.00–29.6%] in ≥60 years on the liability scale).

### ID and COVID-19 outcomes

ROH calling was performed in the European QC-ed genotyped dataset. Inbreeding depression analyses are described in Materials and Methods section and Supplemental Note.

**Figure 6:**
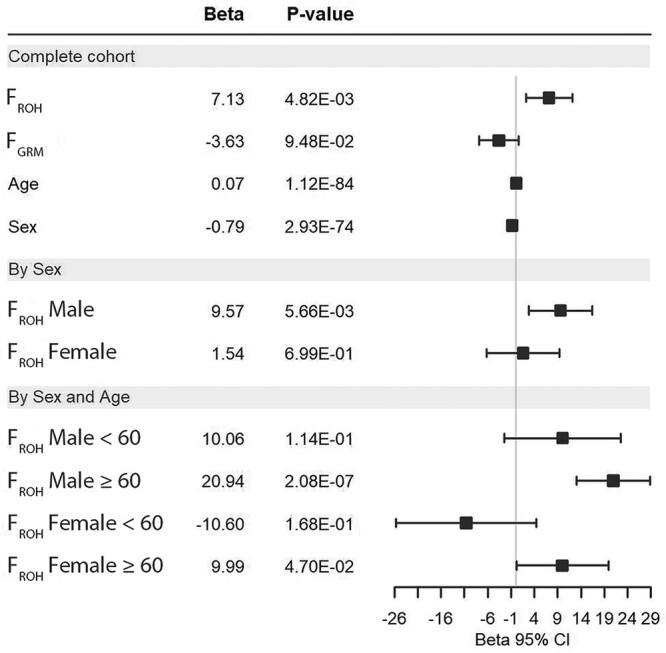
Effect of the ID on COVID-19 hospitalization in the SCOURGE cohort. Forest plots are shown for global analyses as well as for sex and age-disaggregated analyses.

The median genomic inbreeding coefficient, *F*_ROH_, for the entire SCOURGE study was 0.0048 (IQR = 0.004). No differences were detected between males (*F*_ROH_ = 0.004, IQR = 0.0035) and females (*F*_ROH_ = 0.0056, IQR = 0.0038), or between younger and older individuals (*F*_ROH individuals < 60_  _years old_ = 0.004, IQR = 0.0035; *F*_ROH individuals ≥ 60 years old_ =  0.0052, IQR = 0.0047, respectively; Supplementary Material, Fig. S6). Regarding the ID in COVID-19 outcomes, we detected a positive association of the F_ROH_ in COVID-19 hospitalization risk (Fig. 6), severity risk and risk for critical illness (Supplementary Material, Table S7). Our results showed that the hospitalization odds for COVID-19 patients with an *F*_ROH_ = 0.0039 were 380% higher than individuals with *F*_ROH_ = 0. No effect of the genomic relationship matrix (*F*_GRM_) was found.

To assess whether ID in COVID-19 hospitalization in SCOURGE was different between sexes, we first tested the interaction between F_ROH_ and biological sex. F_ROH_, sex and the interaction of both (*F*_ROH_:Sex) were significant (*F*_ROH_ = 4.7 × 10^−3^, Sex = 1.0 × 10^−112^, *F*_ROH_:Sex = 1.2 × 10^−3^). This interaction was significant when comparing the hospitalized COVID-19 patients with different controls (A2 and C analyses, see Supplementary Material, Table S8). This interaction was also found significant with severity, but not with critical illness (Supplementary Material, Table S8). In sex-disaggregated analyses, we observed a sex-specific effect of inbreeding. F_ROH_ was significant in hospitalized males but not in females (Fig. 6 and Supplementary Material, Table S8). This sex-specific effect was also observed with severity and for critical illness (Supplementary Material, Table S8). We then assessed whether ID in COVID-19 hospitalization was different by age. We detected a significant interaction between age and *F*_ROH_ for the three outcomes considered (hospitalization, severity and critical illness) (Supplementary Material, Table S9). After disaggregating SCOURGE by sex and age (<60, ≥60), we found that the ID for hospitalization and severity were significant mainly in older males (Fig. 6 and Supplementary Material, Table S9). We detected significant ID for hospitalization and severity in males ≥ 60 years old, but it was marginally significant in females (Fig. 6 and Supplementary Material, Table S9). Age and sex-specific effects in hospitalization and severity were robust across different experimental designs using different control groups (Supplementary Material, Fig. S7).

Finally, we then aimed to replicate the ID results with hospitalization, assessing sex and age-specific effects, in a 4418 case-enriched European cohort made of 16 studies from nine countries. Median *F*_ROH_ in this other European cohort was slightly higher than that of SCOURGE, 0.05 (0.009–0.0577). A positive and significant ID in COVID-19 hospitalization was detected in this other European cohort when the entire cohort was considered (*F*_ROH_ Beta = 18.22, *P* = 3.33 × 10^−3^). F_GRM_ was not significant (*F*_GRM_ Beta = −7.34, *P* = 0.240). ID was also detected in hospitalized COVID-19 males but not in females (Male *F*_ROH_ Beta = 16.22, *P* = 3.31 × 10^−3^; Female *F*_ROH_ Beta = 15.65, *P* = 0.269). *F*_GRM_ was not significant in males or in female analyses. When disaggregating by age, it was possible to detect significant ID in hospitalization only in males ≥60 years old (*F*_ROH_ Beta = 36.16, *P* = 3.34 × 10^−3^) (Supplementary Material, Table S10).

No evidence was found of major loci that may be exerting large effects. Rather, polygenicity seems to underlie the ID association. Different islands of ROH (ROHi) and regions of heterozygosity (RHZ) were found to be unique for hospitalized COVID-19 individuals (males and females) and non-hospitalized males, respectively (Supplementary Note, Supplementary Material, Table S11). Enrichment analysis of pathways based on the protein coding genes present in ROH islands were also different between sexes (Supplementary Note, Supplementary Material, Table S12), revealing links with coagulation and complement pathways in males.

## Discussion

Here we report the replication of six COVID-19 loci across analyses and provide evidence supporting three additional loci, one of them specifically detected among females. Besides, our analyses provide new insights into disease severity disparities by sex and age and support the necessity of similarly stratified studies to increase the possibility of detecting additional risk variants. Our GWAS constitutes the largest study on COVID-19 genetic risk factors conducted in Spain, with an intrinsic design benefit that SCOURGE includes detailed clinical and genetic information gathered homogeneously across the country. Besides, the study included patients from the whole spectrum of COVID-19 severity covering from asymptomatic to life-threatening disease. To date, most research on COVID-19 disease has focused on respiratory failure. However, the inclusion of a severity scale provided a unique opportunity to assess whether previously reported loci combined into a GRS model were associated with differential risk by strata. We warn, however, that the GRS model findings should be interpreted with caution as sex and age-differential results in some of the severity strata needs appropriate replication. Association was tested for four main variables: infection, hospitalization, severe illness and critical illness, and using different definitions of controls to align with the COVID-19 HGI. Irrespective of the tested outcomes or the definition of controls, the results were very similar, supporting the use of population controls to increase power in these studies and the utility of using hospitalization as a proxy of severity. However, our results from the GRS analysis reported a need to maintain separated categories for medium–severe and critical illness.

We observed larger heritability differences by age groups among males than among females for COVID-19 hospitalization, which have diverse support from the literature. On the one hand, there is robust evidence supporting that the presence of X-linked deleterious variants in the *TLR7* gene are causal for life-threatening COVID-19 only affecting males (19–21). Of note, most of these severe COVID-19 male patients were younger than 60 years (21). On the other hand, autoantibodies impairing type-I interferon signaling, which are supported to be strong determinants of critical COVID-19 pneumonia, are preferentially found among males older than 65 years (11,18). Taken together, this reconciles with the idea that non-genetic factors involved in severe COVID-19 are expected among older males.

We clearly replicated previously reported associations at 3p21.31 (near *SLC6A20* and *LZTFL1*-*FYCO1*) (7,9,22,23) and 21q22.11 (in *IFNAR2*) (7,9), and other findings in *ABO*, *OAS1*, *TYK2* and *ARHGAP27*. We also found a differential effect between males and females for SNPs in 3p21.31 and 21q22.11. Although in the meta-analysis with other European studies the leading variants of 3p21.31 reached genome-wide significance in females, there was still a difference in effect size that, considering its magnitude, should not be disregarded. It is important to remark that these association signals found in males were not associated with the presence of comorbidities (see Supplementary Material, Fig. S4). In fact, genetic effects were only found for younger males (<60 years old), consistent with other studies (24) and strongly supporting those comorbidities outweigh genetic effects in disease outcomes in the older patients.

Some novel genome-wide significant signals were found in our study, one in chromosome 19q13.12 (intergenic to *UPK1A* and *ZBTB32*, and also linked to the transcriptional regulation of *ARHGAP33*), and another in chromosome 9p13.3 (intergenic to *AQP7* and *AQP3*). Interestingly, we also found two sex-specific signals: *ELF5* in males and *TLE1* in females. *ELF5* has been recently reported as a new locus associated with critical illness in Europeans (25). Variants of this locus reached genome-wide significance in our male meta-analysis of European cohorts (*P =* 4.1 × 10^−8^). As regards of *TLE1*, this locus should be taken as speculative as the signal did not reach the standard genome-wide significance in the study. However, given that the meta-analysis involved a low number of studies (and the top marker was not imputed in one of them), this result should be taken with caution as further sex-specific studies will be needed to validate this finding.

*TLE1* encodes for the transducin-like enhancer of split 1, a co-repressor of other transcription factors and signaling pathways. Besides repressing the transcriptional activity of FOXA2 and of the Wnt signaling, TLE1 has been shown to negatively regulate NF-κB, which is fundamental in controlling inflammation and the immune response. The deficiency of TLE1 activity in mice results in enhancement of the NF-κB-mediated inflammatory response in diverse tissues including the lung (26). Interestingly, TLE1 is one of the 332 high-confidence SARS-CoV-2 protein–human protein interactions identified so far (27). Taken together, SARS-CoV-2 would be directly targeting the innate immunity and inflammation signaling pathways by interfering with the NF-κB activity. Thus, it is not surprising that TLE1 is a top-ranking regulator of inflammation that allows to transcriptionally distinguish mild from severe COVID-19 (28).

In the 19q13.12 locus, the most biologically plausible genes are *ARHGAP33* (also showing the best functional support based on the fine-mapping variants) and *ZBTB32*. *ARHGAP33* is transcriptionally regulated by IRF1—a prominent antiviral effector and IFN-stimulated gene (29). It also harbors NF-κB binding site that modifies its expression in human lymphoblastoid cell lines by the presence of genetic variants in the binding site linked to many inflammatory and immune-related diseases including sepsis, and bacterial and viral infection (30). Its expression is also altered in human induced pluripotent stem cells-derived pancreatic cultures in response to SARS-CoV-2 infection (31). *ARHGAP33* was identified in an unbiased genome-wide CRISPR-based knockout screen in human Huh7.5.1 hepatoma cells infected by coronaviruses including SARS-CoV-2 and further interactome studies (32). With respect to the transcription factor ZBTB32, it has been shown to impair antiviral immune responses by affecting cytokine production and the proliferation of natural killer and T cells, and the generation of memory cells (33). In single cell studies, transcripts of *ZBTB32* were enriched in T follicular helper cells and were also expressed at significantly higher levels in hospitalized COVID-19 patients (34).

AQP3 is expressed most strongly in the kidney collecting duct, the gastrointestinal tract, large airways (in basal epithelial cells and the nasopharynx), skin and the urinary bladder; whereas AQP7 is expressed primarily in the testis, fat cells and, to a lesser extent in a subsegment of the kidney proximal tubule (35). In addition, AQP3 is upregulated in the lung tissues during viral or bacterial-induced diffuse alveolar damage (36). Based on this, in the fact that SARS-CoV-2 interacts with host proteins with the highest expression in lung tissues (27), and the functional evidence linking the fine-mapped variants with eQTLs in lung tissues, our data support *AQP3* as the most likely 9p13.3 gene driving the association with COVID-19 hospitalization. Many patients develop acute respiratory distress syndrome (ARDS) during severe COVID-19 (37), and one of the hallmarks of ARDS is the increase of fluid volume (edema) in the airspaces of the lung because of an increase in the alveolo-capillary membrane permeability. Some of the aquaporins, including AQP3, essentially function as water transport pores between the airways and the pulmonary capillaries (38), are key in lung fluid clearance and the formation of this lung edema as a consequence of the lung injury (35). In fact, the use of aquaporin modulators in lung inflammation and edema has been proposed for potential use for the treatment of COVID-19 respiratory comorbidity (39).

We have also shown for the first time that COVID-19 severity risk suffers from ID, where individuals with higher levels of homozygosity associate with higher risk of being hospitalized and of developing severe COVID-19. Our results also suggested that autozygous rare recessive variants found in ROH across the genome, rather than homozygous common variants in strong LD, are underlying the ID. Furthermore, the ID is stronger in males than in females suffering from COVID-19 hospitalizations, especially in males ≥ 60 years old. Although these results may be found counterintuitive with the rest of findings, they are supported by the mutation accumulation senescence theory. Under this theory, alleles with detrimental effects that act in late life are expected to accumulate and cause senescence, thus increasing the ID (40). We detected further sex-specific effects of homozygosity through ROHi. In hospitalized males, coagulation and complement pathways, which have been previously associated with severe COVID-19 (41), were enriched among the protein coding genes located in ROHi, highlighting the role of homozygosity whereas the Lectin pathway of complement activation is reported to be involved in the response to SARS-CoV-2 infection (42–44). In hospitalized females, PI3K-Akt signaling genes were found to be enriched in ROH islands, whose networks are affected by a great variety of viruses (45).

Given that the effect of the genetic variants in SARS-CoV-2 severity is clearer among males and previous evidence on this regard, we elucubrate on the role of androgens in COVID-19 severity. Androgenic hormones have been suggested to be responsible of the excess male mortality observed in COVID-19 patients (46), and several lines of evidence suggest that the androgen receptor (AR) pathway is involved in the severity of SARS-CoV-2 infection: (i) A higher mortality rate among men has been established (47); (ii) A substantial proportion of individuals, both males and females, hospitalized for severe COVID-19 have androgenetic alopecia [AGA; (47)] and (iii) Most of the genes on COVID-19 severity in this study have been identified in male-only analyses, and these genes have been shown to interact with the AR. The following lines of evidence suggest the AR pathway is a mechanism responsible for some identified genes-COVID-19 severity relationship: (i) FYCO1 is regulated by the AR (48), and binding sites between the sex hormone receptor AR and FYCO1 have been demonstrated (48,49); (ii) There is a cross-talk between the IFN pathways and the androgen signaling pathways (50), and androgens are regulated by IFNs in human prostate cells (51); (iii) *TMPRSS2*, another gene associated with COVID-19 severity in other studies, is induced by androgens through a distal AR binding enhancer (52); (iv) AR induces the expression of chemokine receptors such as CCR1; (v) Variants of *LZTFL1* gene are likely pathogenic for male reproductive system diseases (53) and (vi) Genetic polymorphisms in the AR (long polyQ alleles ≥23) and higher testosterone levels in subjects with AR long-polyQ appear to predispose some men to develop more severe disease (54). Thus, it is not unexpected to find that antiandrogen treatments are under the focus as treatment options and prophylaxis of severe COVID-19 (47) and that randomized controlled clinical trials with bicalutamide (NCT04374279), degarelix (NCT04397718) and spironolactone (NCT04345887) are currently underway.

## Materials and Methods

### Recruitment of cases and phenotype definitions for the discovery phase

In Spain, 11 939 COVID-19 positive cases were recruited as part of SCOURGE study from 34 centers in 25 cities between March and December 2020. The complete list of hospitals or research centers and the number of samples that each contributed to the study is shown in Supplementary Material, Table S1. Study samples and data were collected by the participating centers, through their respective biobanks after informed consent, with the approval of the respective Ethic and Scientific Committees. The whole project was approved by the Galician Ethical Committee Ref 2020/197. All samples and data were processed following normalized procedures. Study data were collected and managed using REDCap electronic data capture tools hosted at Centro de Investigación Biomédica en Red [CIBER; (55,56); Supplementary Material, Supplemental Note]. Individuals were diagnosed as COVID-19 positive through a PCR-based test (81.7% of cases) or according to local clinical (3.4%) and laboratory procedures (antibody test: 14.2%; other microbiological tests: 0.7%). All cases were classified in a five-level severity scale (Table 1).

Two Spanish sample collections with unknown COVID-19 status were included as general population controls in some analyses: 3437 samples from the Spanish DNA biobank (https://www.bancoadn.org) and 2506 samples from the GR@CE consortium (17). General population controls were collected from branches of the National Blood Service from adult unrelated individuals self-reporting Spanish origin and absence of personal and familial history of diseases including infectious, cancerous, blood and circulatory, endocrine, mental or behavioral, nervous, vision, hearing, respiratory, immunological, bone, congenital, skin and digestive.

### Second phase: the CNIO study

A total of 1598 COVID-19 cases from six different Spanish Biobanks (Biobanco CNIO, Biobanco Vasco, Biobanco Hospital Ramón y Cajal, Biobanco Hospital Puerta de Hierro, Biobanco Hospital San Carlos, and Banco Nacional de ADN) were obtained according to the ethical committee approval CEI PI 34_2020-v2. In addition, 1068 individuals from Spanish DNA biobank with unknown COVID-19 status were included as healthy controls in the analysis whenever necessary. Classification as healthy was based on self-reported absence of cardiovascular, renal, pulmonary, hepatic, hematological illnesses or any other chronic conditions, which require continuous treatment, hepatitis B, C infections or acquired immunodeficiency syndrome (AIDS). No clinical characterization was performed on any subject, no information from medical record was incorporated and no medical testing was performed on these individuals. We will refer to these cases and controls as the Centro Nacional de Investigaciones Oncológicas (CNIO) study.

### Genotyping

The discovery phase samples were genotyped with the Axiom Spain Biobank Array (Thermo Fisher Scientific) following the manufacturer’s instructions in the Santiago de Compostela Node of the National Genotyping Center (CeGen-ISCIII; http://www.usc.es/cegen). This array contains 757 836 markers, including rare variants selected in the Spanish population. Genomic DNA was obtained from peripheral blood and isolated using the Chemagic DNA Blood100 kit (PerkinElmer Chemagen Technologies GmbH), following the manufacturer’s recommendations.

For the second phase study samples, a total of 250 ng of DNA was processed according to the Infinium HTS assay Protocol (Part # 15045738 Rev. A, Illumina), including amplification, fragmentation and hybridization using the Global Screening Array Multi-disease v3.0. This array contains a total of 730 059 markers and was scanned on an iScan platform (Illumina, Inc.). Clustering and genotype calling were performed using Genome Studio v2.0.4 (Illumina, Inc.).

### Quality control

A QC procedure was carried out for the SCOURGE study samples and control datasets. First, a list of probe sets was removed based on poor cluster separation or excessive MAF difference from The 1000 Genomes Project data (1KGP) (57). Then, the following QC steps were applied using PLINK 1.9 (58) and a custom R script. We excluded variants with MAF < 1%, call rate < 98%, a difference in missing rate between cases and controls >0.02, or deviating from Hardy–Weinberg equilibrium (HWE) expectations [*P* < 1 × 10^−6^ in controls, *P* < 1 × 10^−10^ in cases, with a mid-*P* adjustment (59)]. Samples with a call rate < 98% and those in which heterozygosity rate deviated >5 SD from the mean heterozygosity of the study were also removed.

To assess kinship and assign ancestries, autosomal SNPs (MAF > 5%) were pruned with PLINK using a window of 1000 markers, a step size of 80 and a *r*^2^ of 0.1. In addition, high-linkage disequilibrium (LD) regions described in Price *et al*. (60) were also excluded. A subset of 131 937 independent SNPs was used to evaluate kinship (IBD estimation) in PLINK. Given the possible confusion between relatedness and ancestry, we removed only one individual from each pair of individuals with PI_HAT > 0.25 (second-degree relatives) that showed a Z0, Z1 and Z2 coherent pattern (according to theoretical expected values for each relatedness level). The unrelated SCOURGE individuals were merged with samples from 1KGP and the common SNPs were LD-pruned as previously indicated. Ancestry was then inferred with Admixture (61) using the defined 1KGP superpopulations. Those individuals with an estimated probability >80% of pertaining to European ancestry were defined as European (*N* = 15 571).

Genomic PCs were also computed using a LD-pruned (*r*^2^ < 0.1 with a window size of 1000 markers) subset of genotyped SNPs passing quality check for controlling the population structure in the GWAS.

The CNIO study data was filtered following the same QC procedures, where 220 individuals were identified as non-European and, therefore, were excluded from further analysis.

### Variant imputation

Imputation was conducted based on the TOPMed version r2 reference panel [GRCh38; (62)] in the TOPMed Imputation Server. After post-imputation filtering (Rsq > 0.3, HWE *P* > 1 × 10^−6^, MAF > 1%), 15 045 individuals (9371 COVID-19 positive cases and 5674 population controls) and 8 933 154 genetic markers remained in the SCOURGE European study (discovery). The final dataset of the CNIO study (replication) included 2446 individuals (1378 COVID-19 positive cases and 1068 population controls) and 8 895 721 markers. For directly genotyped variants, the original genotype was maintained in place of the imputed data.

### Statistical analysis

Association testing was computed by fitting logistic mixed regression models adjusting for age, sex and the first 10 ancestry-specific PCs. SNPRelate (63) was used for prior LD-pruning and data management. Association analyses were performed in SAIGEgds (64), which implements the SAIGE (65) two-step mixed model methodology and the SPA test while using more efficient objects for genotype storage. A null model was fitted in the first step using the LD-pruned genotyped variants (MAF > 0.5%, missing rate < 98%) to estimate variance components and the genetic relationship matrix. Then, in a second step, association analyses were performed for both genotyped and imputed SNPs. Significance was established at *P* < 5 × 10^−8^ after meta-analysis of the discovery and the second study phases.

To align the results with those from the COVID-19 HGI, three outcomes were evaluated in relation to severity: hospitalization, severe COVID-19 (severity ≥ 3) and very severe COVID-19 (severity = 4, critical illness). For each comparison, three control definitions (A1, A2 and C) were used (Supplementary Material, Table S2).

In addition, the risk to COVID-19 infection was also analysed by comparing all COVID-19 positive cases with control samples from the general population.

All analyses were conducted for each complete dataset and stratified by sex and age (<60 years, ≥60 years). The SNP*sex and SNP*age-interaction terms were tested for each SNP in the subset of clumped signals, adjusting the models for the same covariates.

Then, the main results of both Spanish cohorts (SCOURGE and CNIO) for the overall and sex-stratified analyses were meta-analysed assuming a fixed-effects model using METAL (66).

Because of the similarity of both the SCOURGE and CNIO studies in the clinical variables recorded and, more importantly, in the definition of the severity scale, the leading variants from the significant and validated loci in the hospitalization analysis were also analysed under a multinomial model (Supplementary Material, Supplemental Note).

### Meta-analysis in independent European studies

In order to validate the findings in independent study samples of European ancestry, a meta-analysis of hospitalization risk was performed for the overall and sex-stratified summary statistics of both Spanish cohorts (SCOURGE and CNIO) and other four sex-stratified Europeans studies from the COVID-19 HGI consortium (BelCOVID, GenCOVID, Hostage-Spain and Hostage-Italy).

### Bayesian fine-mapping of GWAS findings

Credible sets were calculated for the GWAS loci to identify a subset of variants most likely containing the causal variant at 95% confidence level, assuming that there is a single causal variant and that it has been tested. We used *corrcoverage* for R (67) to calculate the posterior probabilities of the variant being causal for all variants with *r*^2^ > 0.1 with the leading SNP and within 1 Mb. Variants were added to the credible set until the sum of the posterior probabilities was ≥0.95. VEP (https://www.ensembl.org/info/docs/tools/vep/index.html) and the V2G aggregate scoring from Open Targets Genetics (https://genetics.opentargets.org) were used to annotate the most prominent biological effects of the variants in the credible sets.

### Genetic risk score

A GRS was created for the SCOURGE cohort individuals and population controls using the list of SNPs associated with hospitalization, severity or risk in the meta-analysis performed by the COVID-19 HGI [see Supplementary Material, Table S2 in (9)] to appraise its prediction power of the severity scale in SCOURGE. Details of this analysis can be found in Supplementary Note.

### SNP-heritability of COVID-19 severity

We relied on GCTA-GREML 1.93.2beta (68) to assess the heritability of severe COVID-19 symptoms among SCOURGE patients, excluding those with cryptic relatedness or with missing genotypes above 0.5% and assuming a prevalence of COVID-19 hospitalization of 0.5%. This analysis considered all patients (modelling for age, sex, sex*age and the 10 first PCs), and males and females separately (modelling for age and the 10 first PCs). We also partitioned the variance to assess the heritability changes among the patients <60 or ≥60 years old. We focused on the 547 206 autosomal variants with MAF > 1% and missingness <0.5%. Assuming 0.5% of prevalence of severe COVID-19, and at least 1500 cases and 1500 controls per stratum, we estimate >97.9% power to detect a heritability >0.2.

### ROH calling

The ROH segments longer than 300 Kb were called in SCOURGE using PLINK 1.9 in the European QC-ed genotyped dataset (before imputation) with the following parameters: *homozyg-snp 30*, *homozyg-kb 300*, *homozyg-density 30*, *homozyg-window-sn 30, homozyg-gap 1000, homozyg-window-het 1, homozyg-window-missing 5* and *homozyg-window-threshold 0.05.* No LD pruning was performed.

### Calculating genomic inbreeding coefficients

Different genomic inbreeding coefficients were calculated (69):

*F*_ROH_ measures the actual proportion of the autosomal genome that is autozygous above a specific threshold of minimum ROH length.

$$ {F}_{\mathrm{ROH}}=\frac{\sum_{i=1}^n\mathrm{ROH}>1.5\ \mathrm{Mb}}{3\ \mathrm{Gb}} $$

*F*_GRM_ is an alternative genomic inbreeding coefficient that was obtained using PLINK’s parameter -ibc (Fhat3). This coefficient is described by Yang *et al*. (68) where *N* is the number of SNPs, *p*_i_ is the reference allele frequency of the *i*th SNP, and *x*_i_ is the number of copies of the reference allele. The reference allele frequencies were site-specific and included only variants with MAF > 0.05.

$$ {F}_{\mathrm{GRM}}=\frac{1}{N}\sum_i^n\frac{\left({x}_i^2-\left(1+2{p}_i\right){x}_i+2{p}_i^2\right)}{2{p}_i\left(1-{p}_i\right)} $$

### Testing and replicating the ID

Inbreeding depression is defined as the change in the mean phenotypic value in a population because of inbreeding (12,13). The ID was modelled in SCOURGE by a multiple logistic regression. The covariables used in this study were sex, age and the first 10 PCs.

The results were replicated in a cohort gathered by Nakanishi *et al*. (24). This consists of clinical and genomic data from 4418 individuals of European ancestry (3946 hospitalized COVID-19 cases and 422 controls): 2597 males (1072 males < 60 years old, 1525 males ≥ 60 years old) and 1821 females (808 females < 60 years old, 1013 females ≥ 60 years old). The cohort was built by harmonizing individual-level data from 16 different studies (24). The *F*_ROH_ and *F*_GRM_ coefficients were obtained following the procedure explained previously. The model described previously with the same covariables (age, sex and the first then PCs) was applied in this cohort.

Genome-specific effects on COVID-19 severity and hospitalization were tested through ID in genomic windows, ROH islands (ROHi) and regions of heterozygosity (RHZ) (Supplementary Material, Supplemental Note).

## Supplementary Material

HMG-2022-CE-00087_Cruz_Supplementary_Material_ddac132

HMG-2022-CE-00087_Cruz_Supplementary_tables_ddac132
