# Supplementary material for: Novel genes and sex differences in COVID-19 severity
Source: Hum Mol Genet. 2022 Jun 16;31(22):3789–806. doi: 10.1093/hmg/ddac132 (PMC9652109; doi:10.1093/hmg/ddac132)
Supplement: HMG-2022-CE-00087_Cruz_Supplementary_Material_ddac132 [file hmg-2022-ce-00087_cruz_supplementary_material_ddac132.docx]

**Supplementary Material for**

**Novel genes and sex differences in COVID-19 severity**

**Table of Content**

1. **Supplemental Figures**
2. **Supplemental Note**
   1. **Research electronic data capture (REDCap)**
   2. **Genetic risk score and multinomial regression on severity scale**
   3. **Evaluating the associations of leading SNPs in relation with comorbidities**
   4. **Measuring genome-specific effects on COVID-19 severity and hospitalization**
3. **Supplemental references**
4. **A full list of cohort members and affiliations**
   1. **SCOURGE cohort group**
   2. **HOSTAGE cohort group**
   3. **GR@ACE cohort group**

**Supplemental Figures**

**
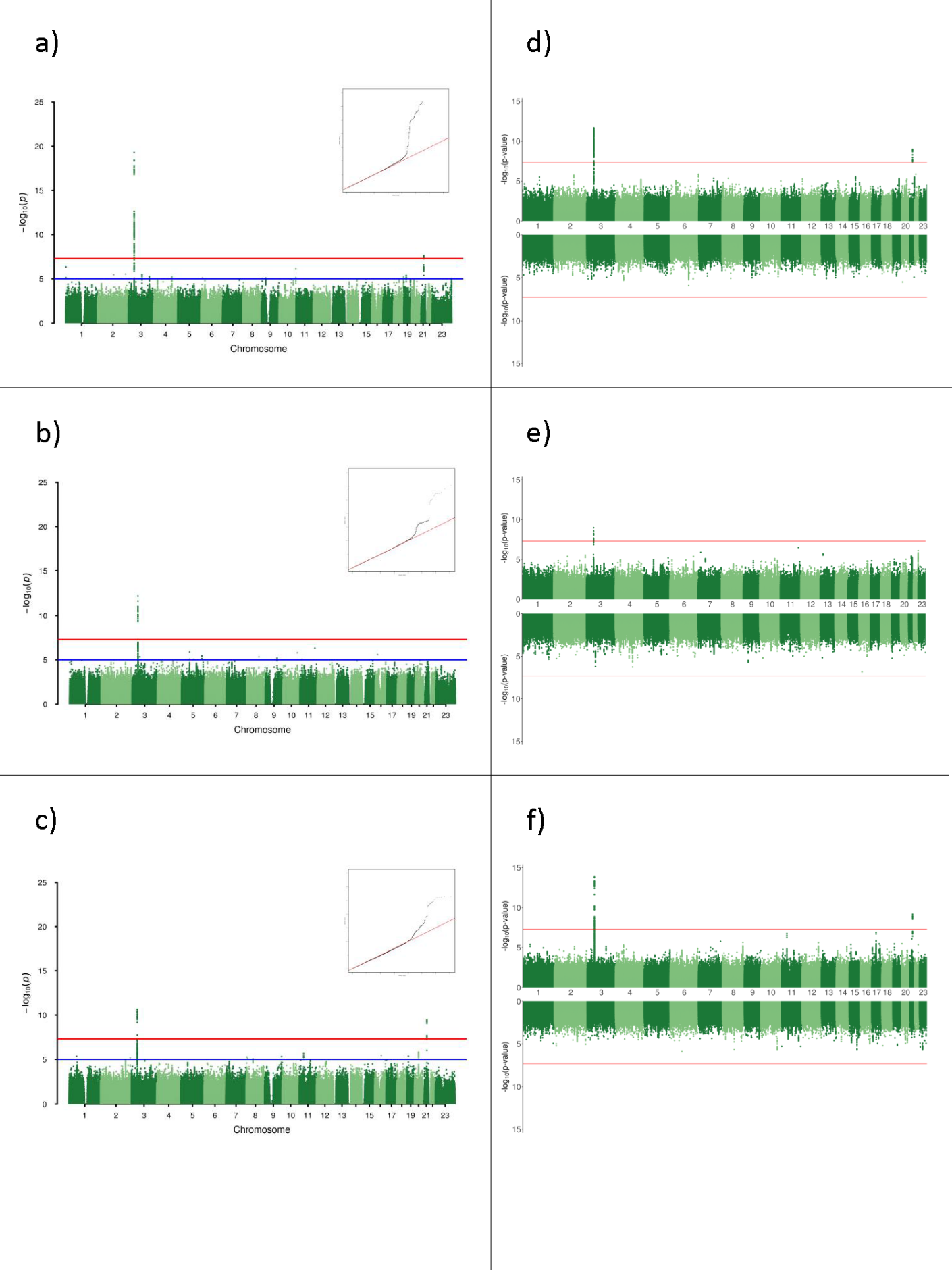
**

**Fig. S1.** Manhattan plots and quantile-quantile plots of the GWAS results of the A1 analysis from the overall SCOURGE study and Miami plots for the sex-stratified analysis (top: males, bottom: females). a, b, c: Manhattan plots for severe illness, critical illness, and risk of infection, respectively. d, e, f: Miami plots for sex-disaggregated analyses in severe illness, critical illness, and risk of infection, respectively.

**
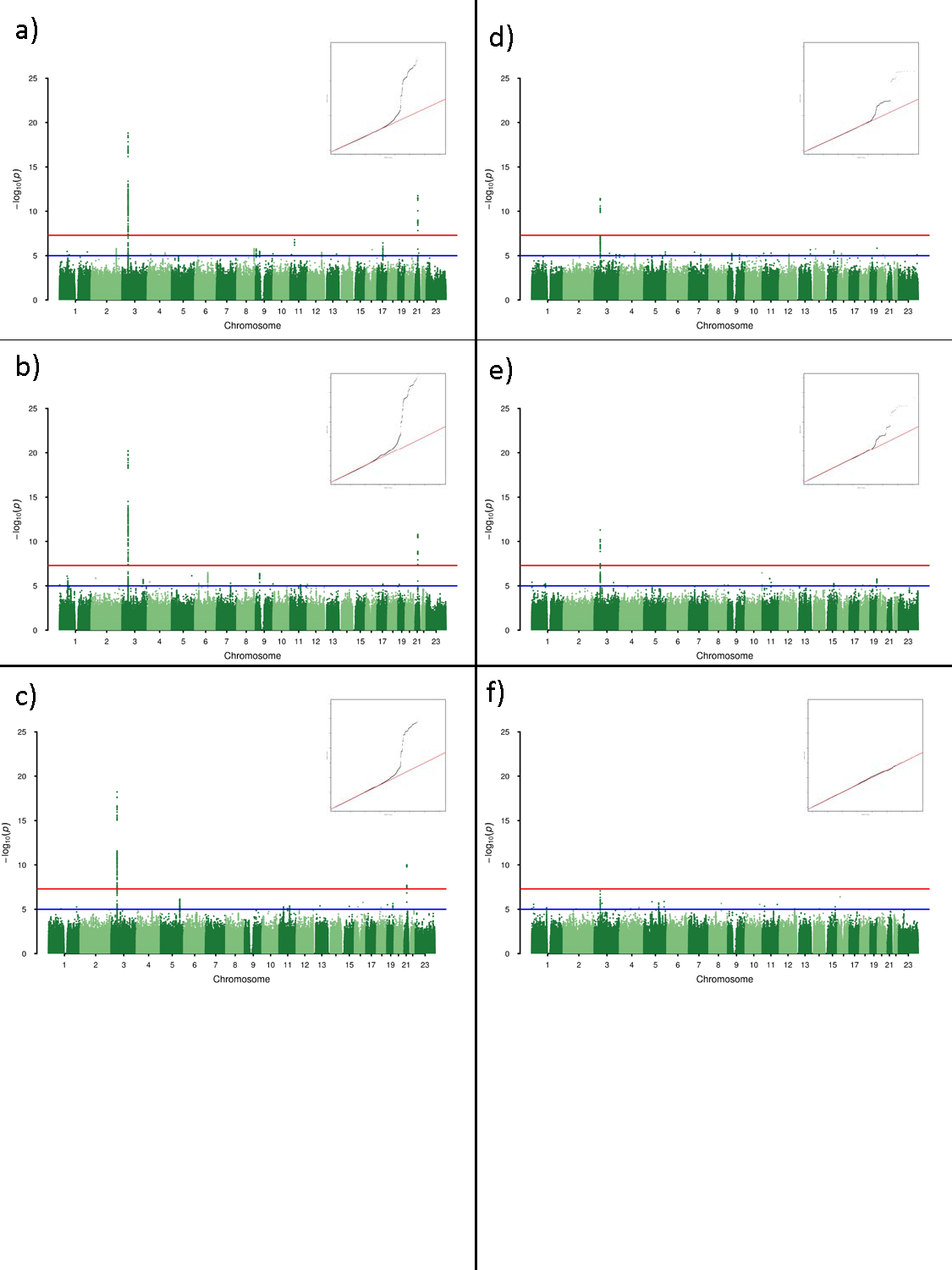
**

**Fig. S2.** Manhattan plots and quantile-quantile plots of the GWAS results for the overall SCOURGE study corresponding to A2 (left) and C (right) analyses for hospitalization (a, d), severe illness (b, e), and critical illness (c, f).


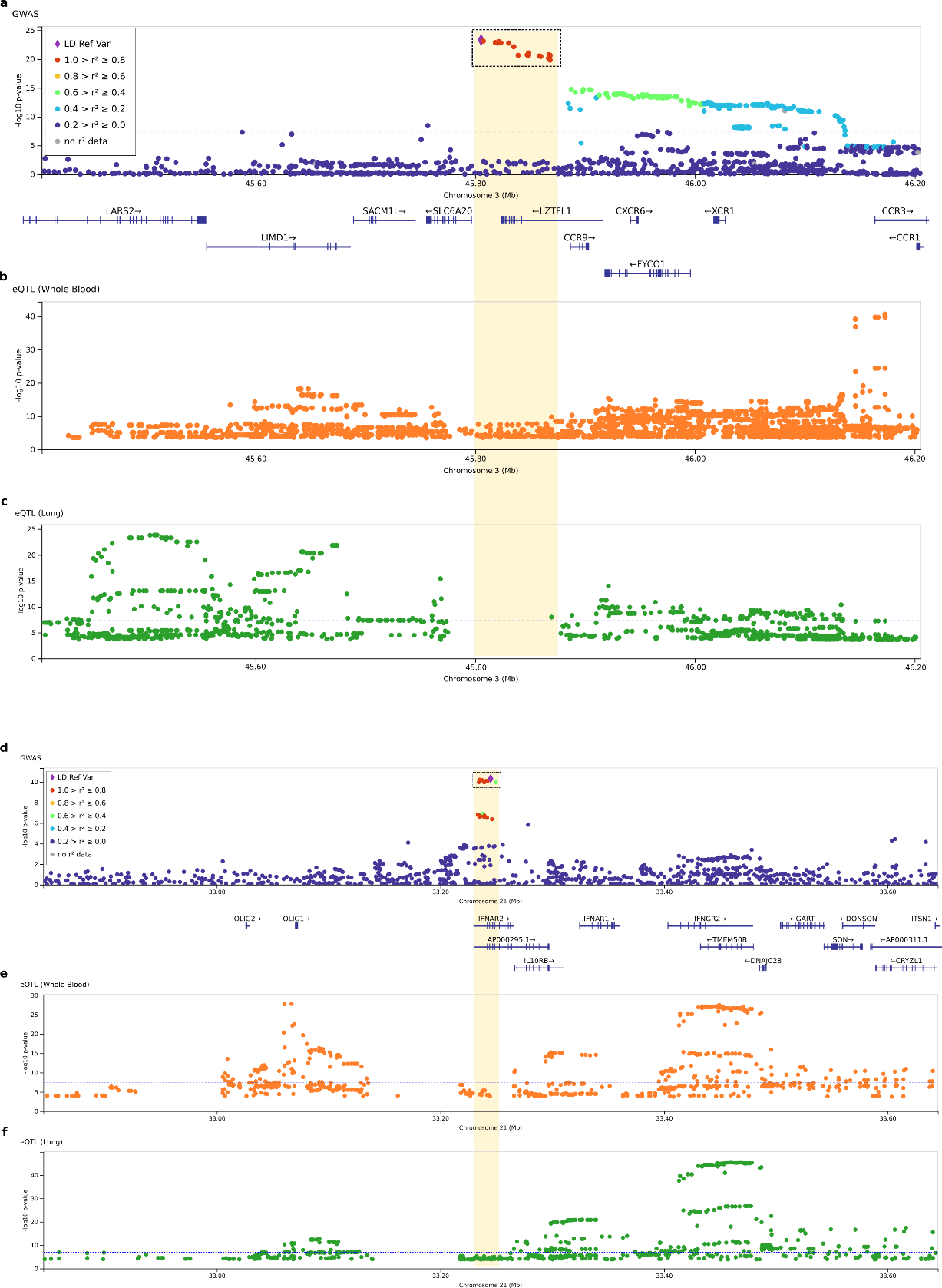


**Fig. S3.** Regional plots of two previously reported association signals in 3p21.31 (a-c) and 21q22.11 (d-f). The x axes reflect the chromosomal position, and the y axes the -log(p-value) in the SCOURGE study. On panels a) and d), the sentinel variant is indicated by a diamond and all other variants are colour coded by their degree of linkage disequilibrium with the sentinel variant in Europeans. Credible sets for each signal are shown by squares. The horizontal dotted blue line corresponds to the threshold for genome-wide significance (*p*=5x10^-8^). In the rest of panels, the x axes reflect the chromosomal position, and the y axes the -log(p-value) resulting from the eQTL analyses in whole blood (b and e) and in the lung (c and f) whenever a significant finding is available from GTEx v8.

**
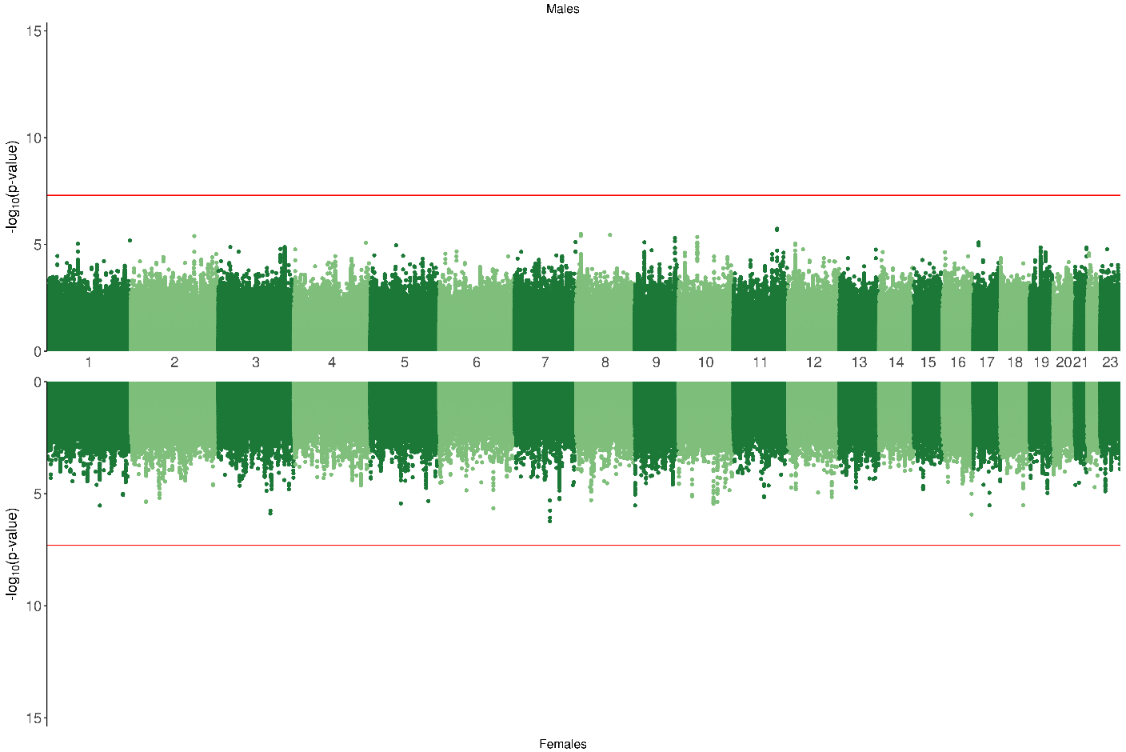
**

**Fig. S4.** Miami plot of the GWAS results of SCOURGE for sex-disaggregated analyses of the presence of comorbidities. Top: males; bottom: females


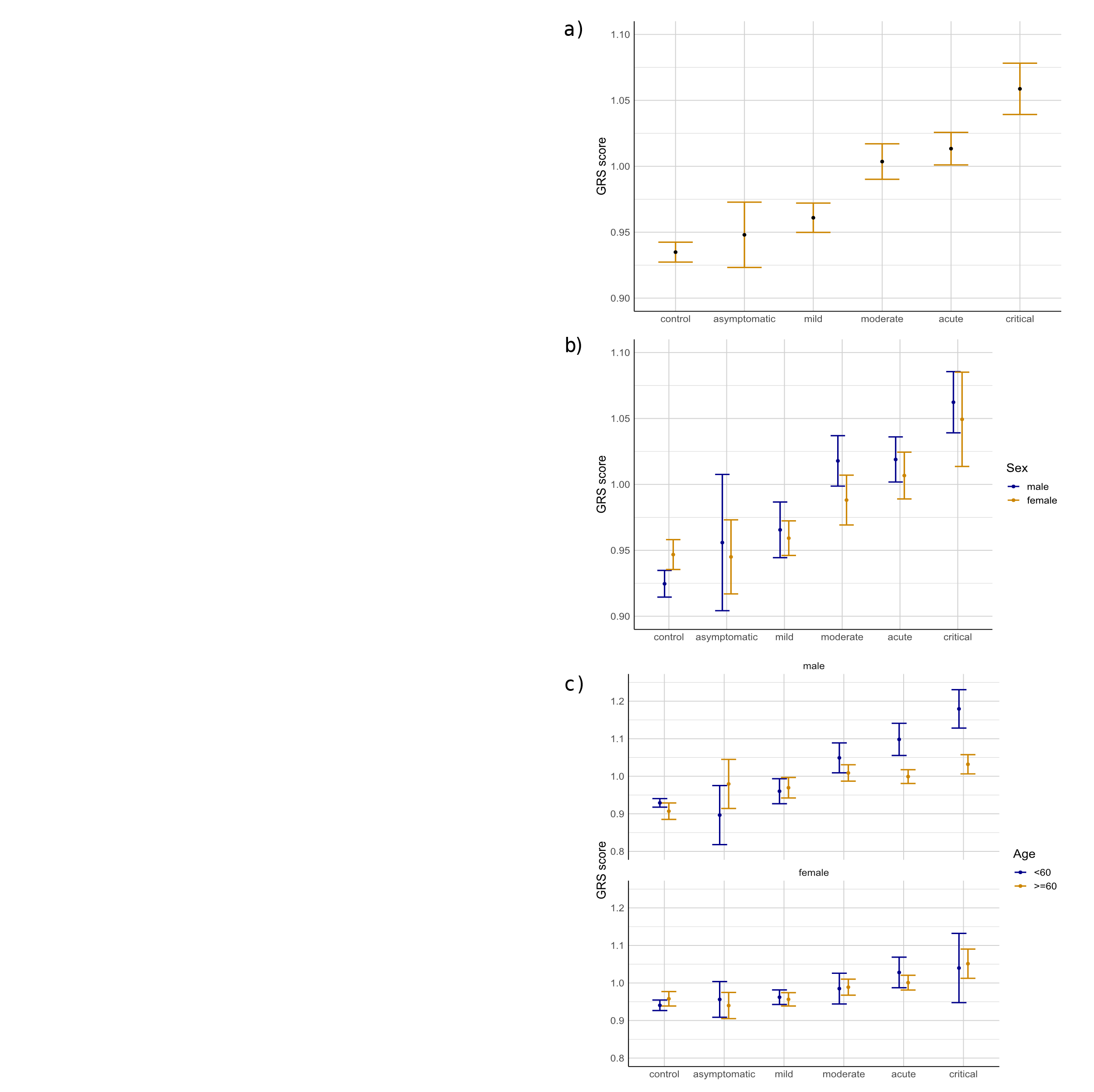


**Fig. S5.** Estimates of the GRS mean (and 95% confidence interval) built from the 13 leading variants found by the COVID-19 HGI GWAS for each category of the severity scale recorded in SCOURGE in global (a), sex-disaggregated (b) and sex-age disaggregated analysis (c).


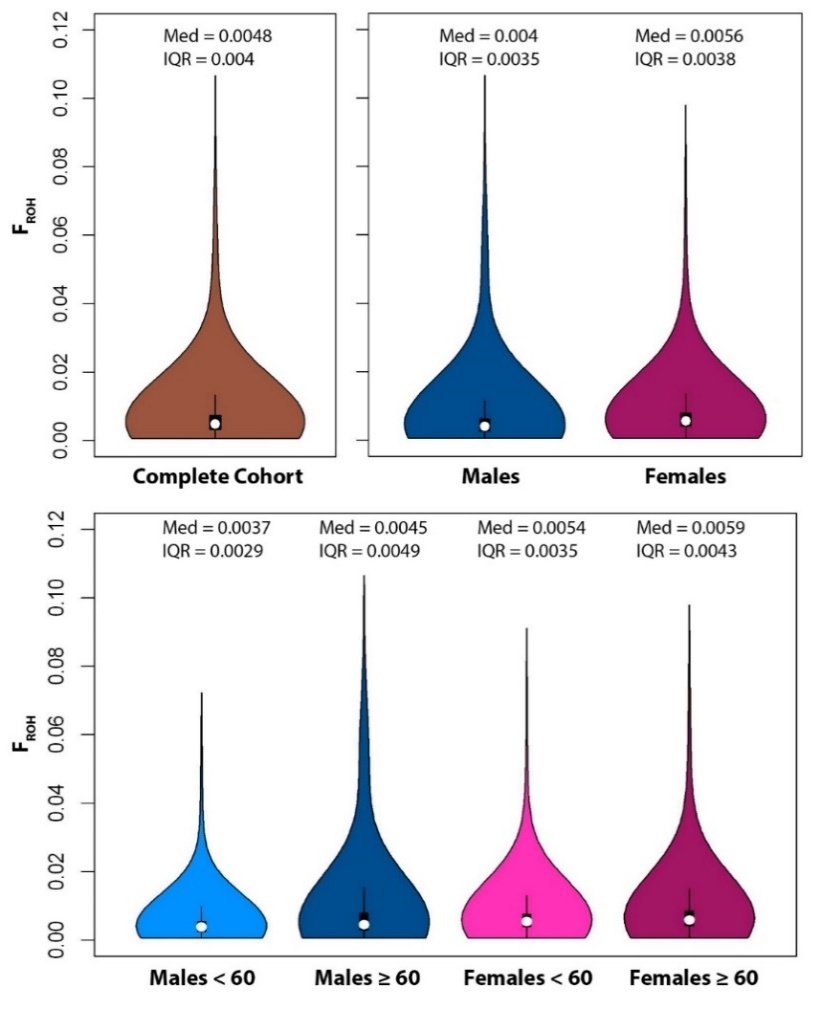


**Fig. S6.** Violin plots showing the distribution of ROH longer than 1.5 Mb for different population groups in the SCOURGE study. Median and interquartile range are shown for each group.


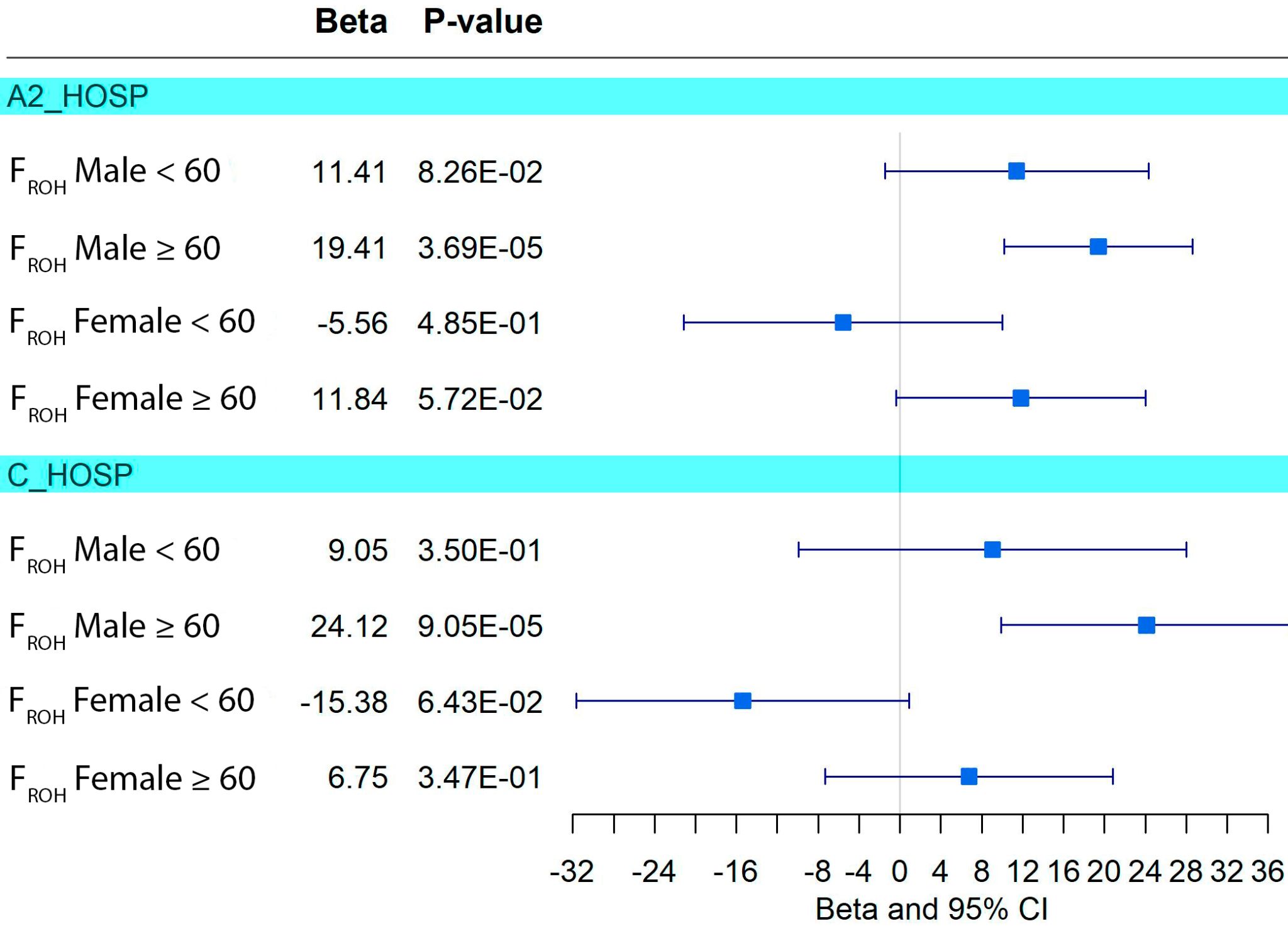


**Fig. S7.** Effect of the inbreeding depression on COVID 19 hospitalization using different control groups. Two different population groups were used as control group: 1) Healthy COVID-19 negative individuals, and 2) Non-hospitalized COVID-19 positive individuals. Forest plots are shown for individuals disaggregated by sex and age.

**
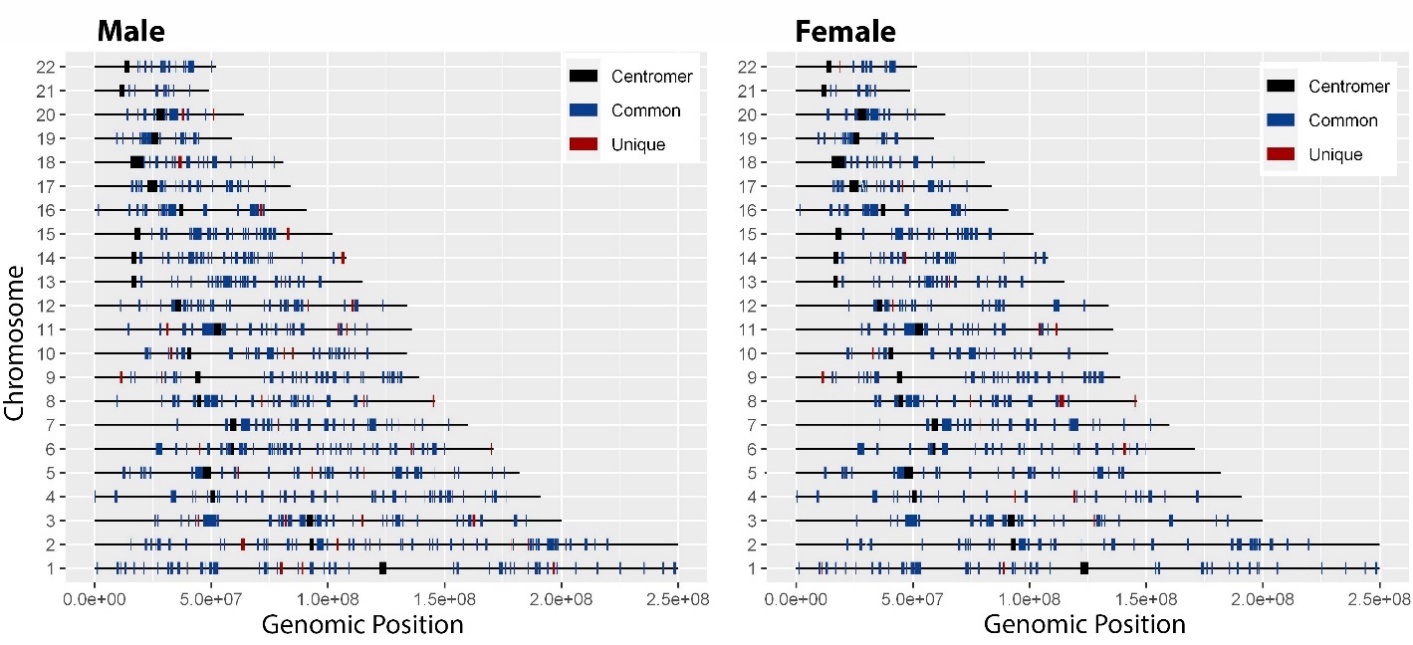
**

**Fig. S8.** Genomic representation of the chromosomal location and size of the runs of homozygosity islands (ROHi) for hospitalized males and females in the SCOURGE study. Unique ROHi of hospitalized males and females are shown in red. Common ROHi between hospitalized and non-hospitalized individuals are shown in blue.


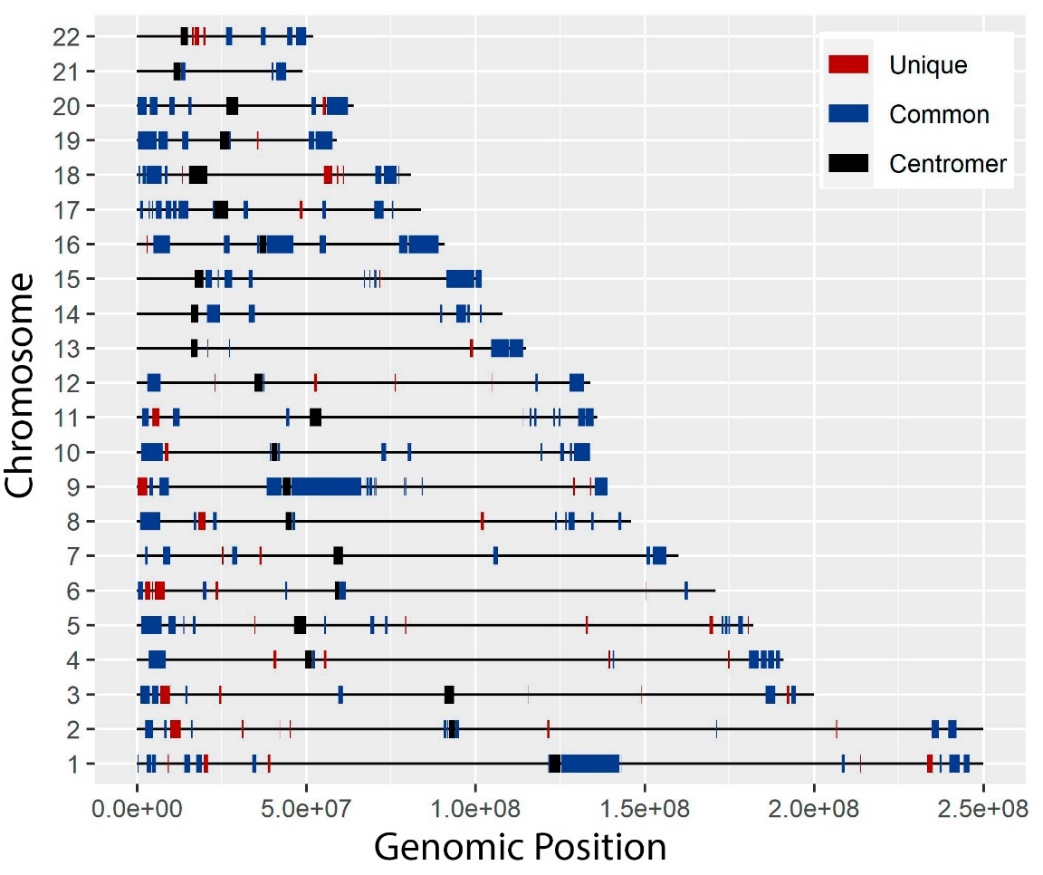


**Fig. S9.** Genomic distribution of regions of heterozygosity (RHZ). Genomic representation of the chromosomal location and size of regions of heterozygosity for non-hospitalized males. Unique RHZ of non-hospitalized males are shown in red. Common ROHi between hospitalized and non-hospitalized males are shown in blue.

**Supplemental Note**

**Research electronic data capture (REDCap)**

REDCap tools (https://www.project-redcap.org), hosted at Centro de Investigación Biomédica en Red (CIBER), was used to collect and manage the demographic, epidemiological, and clinical variables, together with the results of laboratory tests and imaging studies.

REDCap is a secure, web-based software platform designed to support data capture for research studies, providing 1) an intuitive interface for validated data capture; 2) audit trails for tracking data manipulation and export procedures; 3) automated export procedures for seamless data downloads to common statistical packages; and 4) procedures for data integration and interoperability with external sources.

**Genetic risk score and multinomial regression on severity scale**

A genetic risk score (GRS) was created for the SCOURGE cohort individuals and population controls using the list of SNPs associated with hospitalization, severity, or risk in the meta-analysis performed by the COVID-19 HGI (see Supp Table 2 in [9]). Both for males and females, we used the reported effects as weights and prioritized the hospitalization weight for variants significantly associated in the three analyses. To evaluate the existence of genetic risk differences along the disease stages, we fitted an ANOVA using the six-level severity scale (controls from the general population and the five severity levels defined in **Table 1**) as the independent variable. A *post hoc* Duncan test was performed to statistically assess the pairwise differentiation between the levels.

The average values of the GRS for each of the severity scale levels of SCOURGE were statistically different between the six levels in global (*F*_5,14547_=50.77, *p*<2x10^-16^) and the sex-stratified analyses (females: *F*_5,7382_=12.7, *p=*2.58x10^-12^; males: *F*_5,7157_=40.01, *p=*<2x10^-16^). (**Fig. S5a, S5b**). Results were similar when controls from general population were excluded (global ANOVA for five levels: *F*_4,8874_=25.97, *p*<2x10^-16^; females: *F*_4,4753_=10.30, *p=*2.62x10^-8^; males: *F*_4,4114_=10.47, *p=*1.94x10^-8^). Duncan's *post hoc* test did not support differentiation between some of the severity levels, roughly defining three classes: one comprising the controls, the asymptomatic and mild cases; another with moderate and severe cases; and one with the critical cases. The GRS mean among cases was not equal for both sexes (*t*_8994.5_=-5.21, *p=*1.98x10^-7^). However, we did not find any statistically significant differences between sexes within each category (**Fig. S5b**). When the GRS was performed disaggregating by age (<60/≥60 years old) and sex simultaneously, we found the same three severity classes in the subgroup of males <60 years old, supporting the importance of this group in the overall findings **(Fig. S5c**). In fact, significant differences were found between both age groups within males with severe (*t*_359.2_ = 4.18 , *p*= 3.6x10^-5^) and critical illness (*t*_815_ = 5.12, *p*= 3.9x10^-7^).

As the GRS analysis outlined the existence of three severity categories in the SCOURGE study, as opposed to the clinically-based six-level scale, we used the multinomial model to test the association of this three-level severity scale (“mild”: control+asymptomatic+mild severity level; “intermediate”: intermediate+severe cases; “severe”: very severe cases) with the clumped loci that reached genome-wide significance in the meta-analysis of SCOURGE and CNIO studies (**Table 3**). Multinomial regressions were performed with the *mlogit* R library [1]. The null hypothesis for the leading variants was tested with the likelihood-ratio test. **Table S6** shows the results of multinomial regression for both the SCOURGE and CNIO studies. The SNPs showing a p-value < 0.0056 (Bonferroni adjusted threshold of 0.05/9) were considered significant. All variants remained significantly associated with the phenotype in the SCOURGE study, yet only four variants (three in 3p21.31 and the one in 9p13.3) were significantly associated in the CNIO cohort**.**

**Evaluating the associations of leading SNPs in relation with comorbidities**

Further analyses were carried on hospitalized patients from the SCOURGE study to exclude a confounder effect of comorbidities in the genetic associations reported in this study. Firstly, we performed sex-disaggregated GWAS analyses on the presence/absence of comorbidities. No genome-wide significant associations were found, concluding that there is no evidence of direct association of comorbidities with the reported sex-specific signals (see **Fig. S4**).

Additionally, we adjusted the logistic models by the comorbidities of **Table 2** (vascular, cardiac, nervous, digestive, onco-haematological, or respiratory) for each of the leading variants depicted in **Table 3** and **Table 4**, adjusting also for age, sex, and 10 PCs. This confirmed that none of the leading variants was individually associated with any of the comorbidities recorded. Besides this, we also confirmed that there was a lack of confounding with the most frequent specific comorbidities (arterial hypertension, hypercholesterolemia, diabetes, COPD or other chronic respiratory diseases, and obesity).

**Measuring genome-specific effects on COVID-19 severity and hospitalization**

Different approaches were used to learn more about the genetic architecture of COVID-19 severity, namely the assessment of inbreeding depression (ID) in genomic windows, of the islands of runs of homozygosity (ROHi), and of the regions of heterozygosity (RHZ).

First, region-dependent ID was tested in the SCOURGE study by assessing the association of hospitalization and severity with ROH in nearly a thousand 3 Mb-wide windows along the genome (significance established at *p*<5x10^-5^ after Bonferroni correction). We found no evidence of major loci that may be exerting large effects, rather the ID was polygenetic in origin.

ROHi are defined as regions in the genome where the proportion of individuals of a population deviates from the expected under a binomial distribution. These regions have been found to be enriched with protein coding genes under selection [2, 3]. To search for ROHi in the SCOURGE study, a sliding window of 100 kb was used. In every 100 kb genomic window, the number of subjects with ROH was obtained and a binomial test was applied (threshold for significance established at *p*<2x10^-5^, corresponding to an adjustment for 2,500 windows). To prevent sampling bias, a resampling approach was followed. ROH from 100 men and women separately in both hospitalized and non-hospitalized groups were resampled (with replacement) 500 times and each replicate followed the above indicated methodology. Lastly, consecutive windows found to be statistically significant in at least 400 resampling events were considered as a part of the same ROHi. It was considered that both groups had the same specific ROHi if they shared ≥75% of their genomic positions. Protein coding genes present in the ROHi were obtained using the *biomaRt* R package and Ensembl database and an enrichment pathway analysis was done on the gene lists using g:Profiler (<https://biit.cs.ut.ee/gprofiler/gost>, last access: August 23 2021). We found 592 ROHi in hospitalized males, 38 of them (6.4%) were unique to this group and were not found in non-hospitalized males (**Fig. S8**, **Table S11**). A total of 152 protein coding genes were present in those 38 unique ROHi. In **Table S12** we show an enrichment analysis of pathways based on those 152 protein-coding genes, strikingly revealing links with coagulation and complement pathways. Different ROHi were found to be unique for hospitalized COVID-19 females (**Fig. S8**, **Table S11**). From a total of 433, 19 unique ROHi with 44 protein-coding genes were found in hospitalized females. Instead of coagulation or the complement, other pathways were enriched among females (**Table S12**).

Finally, we searched for RHZ, where ROH are scarce or absent. To search for RHZ, an extra step of QC consisting of removing the SNPs in LD using PLINK was performed before calling for ROH. ROH longer than 100 Kb were called for this analysis and a 100 Kb sliding window was used. Two different cut-offs were considered to call RHZ in each window: a) No individual is homozygous, or b) 2% or fewer of the individuals are homozygous. Consecutive windows that fulfilled this requirement were considered part of the same RHZ. Among males, we found a total of 239 RHZ in non-hospitalized control group (non-hospitalized COVID-19 and population controls) and 214 RHZ in hospitalized COVID-19 patients. A total of 61 of the RHZ present in non-hospitalized individuals were found to be unique of this group (**Fig. S9**, **Table S11**). Unique RHZ in non-hospitalized COVID-19 patients involved a total of 707 protein-coding genes. A total of 33 pathways were significantly enriched from this gene list, being olfactory receptor activity and sensory perception of smell the most significant ones (**Table S12**). Surprisingly, we found 36 RHZ, where no individual has ROH, in both hospitalized and non-hospitalized COVID-19 individuals. These 36 RHZ involved 67 genes related to olfactory receptors, spermatogenesis, and survival of motor neurons.

**Supplemental references**

1. Croissant, Y. mLogit: Random Utility Models in R. Journal of statistical software, 95(11) doi: 10.18637/jss.v095.i11 (2020)
2. Ceballos, F.C., Hazelhurst, S. & Ramsay, M. Runs of homozygosity in sub-Saharan African populations provide insights into complex demographic histories. *Hum Genet* **138,** 1123–1142 (2019). <https://doi.org/10.1007/s00439-019-02045-1>
3. Curtis D, Vine AE, Knight J. Study of regions of extended homozygosity provides a powerful method to explore haplotype structure of human populations. *Ann Hum Genet*. 2008;72(Pt 2):261-278. doi:10.1111/j.1469-1809.2007.00411.x

**A full list of cohort members and affiliations**

**SCOURGE Cohort Group**

Javier Abellan^87,88^; René Acosta-Isaac^115^; Jose María Aguado^11,12,13,14^; Carlos Aguilar^15^; Sergio Aguilera-Albesa^16,17^; Abdolah Ahmadi Sabbagh^106^; Jorge Alba^195^; Sergiu Albu^196,197,198^; Karla A.M. Alcalá-Gallardo^199^; Julia Alcoba-Florez^200^; Sergio Alcolea Batres^201^; Holmes Rafael Algarin-Lara^202,123^; Virginia Almadana^18^; Kelliane A. Medeiros^203,204^; Julia Almeida^205,206^; Berta Almoguera^19,2^; María R. Alonso^6^; Nuria Alvarez^6^; Rodolfo Alvarez-Sala Walther^201^; Yady Álvarez-Benítez^202,123^; Felipe Álvarez-Navia^45,32^; Katiusse A. dos Santos^207^; Álvaro Andreu-Bernabeu^20,13^; Maria Rosa Antonijoan^208^; Eleno Martínez-Aquino^209^; Eunate Arana-Arri^21,22^; Carlos Aranda^210,110^; Celso Arango^20,23,13^; Carolina Araque^211,212^; Nathalia K. Araujo^213^; Ana C. Arcanjo^89,214,215^; Ana Arnaiz^62,63^; Francisco Arnalich Fernández^216^; María J. Arranz^24^; José Ramon Arribas Lopez^216^; Maria-Jesus Artiga^25^; Yubelly Avello-Malaver^79^; Carmen Ayuso^19,2^; Belén Ballina Martín^106^; Raúl C. Baptista-Rosas^26,27,28^; Ana María Baldion^79^; Andrea Barranco-Díaz^123^; María Barreda- Sánchez^29,30^; Viviana Barrera-Penagos^79^; Moncef Belhassen-Garcia^31,32^; David Bernal-Bello^217^; Enrique Bernal^29^; Joao F. Bezerra^33^; Marcos A.C. Bezerra^34^; Natalia Blanca-López^218^; Rafael Blancas^219^; Lucía Boix-Palop^35^; Alberto Borobia^220^; Elsa Bravo^221^; María Brion^36,37^; Óscar Brochado-Kith^5^; Ramón Brugada^38,39,37,40^; Matilde Bustos^41^; Alfonso Cabello^222^; Alejandro Cáceres^223,224,43^; Juan J. Caceres-Agra^225^; Esther Calbo^35^; Enrique J. Calderón^42,43,44^; Shirley Camacho^73^; Francisco C. Ceballos^5^; Yolanda Cañadas^110^; Cristina Carbonell^45,32^; Servando Cardona-Huerta^52^; Maria Sanchez Carpintero^210,110^; Carlos Carpio Segura^201^; José Antonio Carrillo-Avila^226^; Marcela C. Campos^89^; Carlos Casasnovas^227,99,2^; Luis Castano^21,46,2,47,48^; Carlos F. Castaño^210,110^; Jose E. Castelao^49^; Aranzazu Castellano Candalija^228^; María A. Castillo^73^; Walter G. Chaves- Santiago^229,212^; Sylena Chiquillo-Gómez^202,123^; Marco A. Cid-Lopez^199^; Oscar Cienfuegos-Jimenez^52^; Rosa Conde-Vicente^50^; Gabriela C.R. Cunha^230^; M. Lourdes Cordero-Lorenzana^51^; Dolores Corella^231,232^; Almudena Corrales^191,192^; Jose L. Cortes-Sanchez^52,53^; Marta Corton^19,2^; Karla S.C. Souza^233^; Fabiola T.C. Silva^89^; Raquel Cruz^1,2,3,4^; Luisa Cuesta^234^; Nathali A.C. Tavares^235^; Maria C.C. Carvalho^236^; David Dalmau^24,35^; Raquel C.S. Dantas-Komatsu^237^; M. Teresa Darnaude^54^; Raimundo de Andrés^238^; Carmen de Juan^239^; Juan De la Cruz Troca^103,104,43^; Carmen de la Horra^44^; Ana B. de la Hoz^21^; Alba De Martino-Rodríguez^55,56^; Marina S. Cruz^240^; Julianna Lys de Sousa Alves Neri^241^; Victor del Campo-Pérez^57^; Juan Delgado-Cuesta^242^; Aranzazu Diaz de Bustamante^54^; Anderson Díaz-Pérez^123^; Beatriz Dietl^35^; Silvia Diz-de Almeida^2,4^; Manoella do Monte Alves^243,244^; Elena Domínguez-Garrido^58^; Lidia S. Rosa^245^; Andre D. Luchessi^59^; Jose Echave-Sustaeta^246^; Rocío Eirós^60^; César O. Enciso-Olivera^211,212^; Gabriela Escudero^247^; Pedro Pablo España^248^; Gladys Mercedes Estigarribia Sanabria^61^; María Carmen Fariñas^62,63,64^; Ramón Fernández^62,249^; Lidia Fernandez-Caballero^19,2^; Ana Fernández-Cruz^250^; Silvia Fernández Ferrero^106^; Yolanda Fernández Martínez^106^; María J. Fernandez-Nestosa^251^; Uxía Fernández-Robelo^65^; Amanda Fernández-Rodríguez^5^; Marta Fernández-Sampedro^62,64,63^; Ruth Fernández^19,2^; Tania Fernández-Villa^66^; Carmen Fernéndez Capitán^228^; Antonio Augusto F. Carioca^252^; Patricia Flores-Pérez^253^; Lácides Fuenmayor-Hernández^123^; Marta Fuertes Núñez^106^; Victoria Fumadó^254^; Ignacio Gadea^255^; Lidia Gagliardi^210,110^; Manuela Gago-Domínguez^8,3^; Natalia Gallego^9^; Cristina Galoppo^119^; Ana García-Soidán^256^; Carlos Garcia Cerrada^87,88^; Aitor García-de-Vicuña^21,46^; Josefina Garcia-García^29^; Irene García-García^220^; Carmen García-Ibarbia^62,64,63^; Andrés C. García-Montero^257^; Leticia García^210,110^; Mercedes García^210,110^; María Carmen García Torrejón^258,88^; Inés García^19,2^; Elisa García-Vázquez^29^; Emiliano Garza-Frias^52^; Angela Gentile^119^; Belén Gil-Fournier^67^; Jéssica N.G. de Araújo^259^; Mario Gómez-Duque^229,212^; Javier Gómez-Arrue^55,56^; Luis Gómez Carrera^201^; María Gómez García^260^; Ángela Gómez Sacristán^261^; Anna González-Neira^6^; Beatriz González Álvarez^55,56^; Fernan Gonzalez Bernaldo de Quirós^68^; Rafaela González-Montelongo^7^; Javier González-Peñas^20,13,23^; Manuel Gonzalez-Sagrado^50^; Hugo Gonzalo Benito^262^; Oscar Gorgojo Galindo^117^; Miguel Górgolas^222^; Florencia Guaragna^119^; Jessica G. Chaux^212^; Encarna Guillen-Navarro^29,187,188,189^; Beatriz Guillen-Guio^191^; Pablo Guisado-Vasco^246^; Luz D. Gutierrez-Castañeda^263,212^; Juan F. Gutiérrez-Bautista^69^; Sara Heili^264^; Rafael H. Jacomo^265^; Estefania Hernandez^98^; Cristina Hernández Moro^106^; Luis D. Hernandez-Ortega^266,267^; Guillermo Hernández-Pérez^45^; Rebeca Hernández-Vaquero^268^; Belen Herraez^6^; M. Teresa Herranz^29^; María Herrera^210,110^; María José Herrero^70,71^; Antonio Herrero-Gonzalez^72^; Juan P. Horcajada^269,270,197,271^; Natale Imaz-Ayo^21^; Maider Intxausti-Urrutibeaskoa^272^; Antonio Íñigo-Campos^7^; María Íñiguez^94^; Rubén Jara^29^; Ángel Jiménez^210,110^; Ignacio Jiménez-Alfaro^273^; Pilar Jiménez^69^; María A. Jimenez-Sousa^5^; Iolanda Jordan^274,275,43^; Rocío Laguna-Goya^76,77^; Daniel Laorden^201^; María Lasa-Lazaro^76,77^; María Claudia Lattig^73,74^; Ailen Lauriente^119^; Anabel Liger Borja^75^; Lucía Llanos^276^; Amparo López-Bernús^45,32^; Miguel López de Heredia^2^; Esther Lopez-Garcia^103,104,43,105^; Eduardo López Granados^277,278,2^; Rosario Lopez-Rodriguez^19,2^; Miguel A. López-Ruz^279,107,280^; Leonardo Lorente^281^; José M. Lorenzo-Salazar^7^; José E. Lozano^282^; María Lozano-Espinosa^75^; Ignacio Mahillo^283,284,192^; Esther Mancebo^76,77^; Carmen Mar^248^; Cristina Marcelo Calvo^228^; Alba Marcos-Delgado^78^; Miguel Marcos^45,32^; Alicia Marín Candon^220^; Pablo Mariscal Aguilar^201^; Laura Martin-Pedraza^218^; Marta Martin-Fernandez^285^; Caridad Martín-López^75^; José-Ángel Martín-Oterino^45,32^; María Dolores Martín^286^; Vicente Martín^78,43^; María M. Martín^287^; María Martín-Vicente^5^; Amalia Martinez^288^; Óscar Martínez-González^219^; Ricardo Martínez^98^; Pedro Martinez-Paz^262^; Covadonga M. Diaz-Caneja^20,23,13^; Oscar Martinez-Nieto^79,74^; Iciar Martinez-Lopez^80,81^; Michel F. Martinez-Resendez^52^; Silvia Martínez^62,64^; Juan José Martinez^99,2^; Ángel Martinez-Perez^82^; Andrea Martínez-Ramas^19,2^; Violeta Martínez Robles^106^; Laura Marzal^19,2^; Juliana A. Mazzeu^83,84,85^; Francisco J. Medrano^42,43,44^; Xose M. Meijome^289,290^; Natalia Mejuto-Montero^291^; Ingrid Mendes^2^; Alice L. Duarte^233^; Ana Méndez-Echevarria^292^; Humberto Mendoza Charris^221,123^; Eleuterio Merayo Macías^86^; Fátima Mercadillo^121^; Arieh R. Mercado-Sesma^266,267^; Pablo Minguez^19,2^; Elena Molina-Roldán^293^; Antonio J J. Molina^78^; Juan José Montoya^98^; Susana M.T. Pinho^203,294,295^; Patricia Moreira-Escriche^239^; Xenia Morelos-Arnedo^221,123^; Rocío Moreno^2^; Victor Moreno Cuerda^87,88^; Antonio Moreno-Docón^29^; Junior Moreno-Escalante^123^; Alberto Moreno Fernández^228^; Patricia Muñoz García^296,192,13^; Pablo Neira^119^; Julian Nevado^2,9,10^; Israel Nieto-Gañán^256^; Vivian N. Silbiger^59^; Rocio Nuñez- Torres^6^; Antònia Obrador-Hevia^80,81^; J. Gonzalo Ocejo-Vinyals^62,64^; Virginia Olivar^119^; Silviene F. Oliveira^89,90,85,91^; Lorena Ondo^19,2^; Alberto Orfao^205,206^; Eva Ortega-Paino^25^; Luis Ortega^297^; Rocio Ortiz-Lopez^52^; Fernando Ortiz-Flores^62,64^; José A. Oteo^195,94^; Manuel Pacheco^98^; Fredy Javier Pacheco-Miranda^123^; Irene Padilla Conejo^106^; Sonia Panadero-Fajardo^226^; Mara Parellada^20,23,13^; Roberto Pariente-Rodríguez^256^; Vicente Friaza^43,44^; Estela Paz-Artal^76,77,92^; Germán Peces-Barba^264,192^; Miguel S. Pedromingo Kus^298^; Celia Perales^255^; Ney P.C. Santos^93^; Genilson P. Guegel^299^; Perez Maria Jazmin^119^; Alexandra Perez^38,37^; Patricia Pérez-Matute^94^; César Pérez^300^; Gustavo Perez-de-Nanclares^21,46^; Felipe Pérez-García^301,302^; Patricia Perez^95^; Luis A. Pérez-Jurado^303,304,2^; M. Elena Pérez-Tomás^29^; Teresa Perucho^96^; Lisbeth A. Pichardo^106^; Adriana P. Ribeiro^203,204,295^; Mel·lina Pinsach-Abuin^38,37^; Luz Adriana Pinzón^229,212^; Jeane F.P. Medeiros^305^; Guillermo Pita^6^; Francesc Pla-Junca^306,2^; Laura Planas-Serra^99,2^; Ericka N. Pompa-Mera^97^; Gloria L. Porras-Hurtado^98^; Aurora Pujol^99,2,100^; María Eugenia Quevedo Chávez^202,123^; Maria Angeles Quijada^208,307^; Inés Quintela^1^; Soraya Ramiro León^67^; Pedro Rascado Sedes^102^; Joana F.R. Nunes^89^; Delia Recalde^55,56^; Emma Recio-Fernández^94^; Salvador Resino^5^; Renata R. Sousa^295,308^; Carlos S. Rivadeneira-Chamorro^212^; Diana Roa-Agudelo^79^; Montserrat Robelo Pardo^102^; Marianne R. Fernandes^93,101^; María A. Rodriguez-Hernandez^41^; Agustí Rodriguez-Palmero^309,99^; Emilio Rodríguez-Ruiz^102,3^; Marilyn Johanna Rodriguez^212^; Fernando Rodriguez-Artalejo^103,104,43,105^; Marena Rodríguez-Ferrer^123^; Carlos Rodriguez-Gallego^310,311^; José A. Rodriguez-Garcia^106^; Belén Rodríguez Maya^87^; Antonio Rodriguez-Nicolas^69^; Ezequiel Rodriguez Novoa^119^; Paula A. Rodriguez-Urrego^79^; Federico Rojo^312^; Andrea Romero-Coronado^123^; Rubén Morilla^44,313^; Filomeno Rondón García^106^; Antonio Rosales-Castillo^314^; Cladelis Rubio^315^; María Rubio^210,110^; Francisco Ruiz-Cabello^69,107,108^; Eva Ruiz-Casares^96^; Juan J. Ruiz-Cubillan^62,64^; Javier Ruiz-Hornillos^109,110,111^; Montserrat Ruiz^99,2^; Pablo Ryan^112,113,114^; Hector D. Salamanca^211,212^; Lorena Salazar-García^73^; Giorgina Gabriela Salgueiro Origlia ^228^; Anna Sangil^35^; Olga Sánchez-Pernaute^316^; Pedro-Luis Sánchez^60,32^; Antonio J. Sánchez López^317^; Clara Sánchez-Pablo^60^; María Concepción Sánchez Prados^201^; Javier Sánchez Real^106^; Jorge Sánchez Redondo^87,318^; Cristina Sancho- Sainz^272^; Esther Sande^319^; Arnoldo Santos^300^; Agatha Schlüter^99,2^; Sonia Segovia^306,320,321^; Alex Serra-Llovich^24^; Fernando Sevil Puras^15^; Marta Sevilla Porras^2,9^; Miguel A. Sicolo^322,323^; Cristina Silván Fuentes^2^; Vitor M.S. Moraes^324^; Vanessa S. Souza^230^; Jordi Solé-Violán^325,192^; José Manuel Soria^82^; Jose V. Sorlí^231,232^; Nayara S. Silva^326^; Juan Carlos Souto^115^; John J. Sprockel^229,212^; José Javier Suárez-Rama^1^; David A. Suarez-Zamora^79^; Xiana Taboada-Fraga^291^; Eduardo Tamayo^116,117^; Alvaro Tamayo-Velasco^118^; Juan Carlos Taracido-Fernandez^72^; Romero H.T. Vasconcelos^235^; Carlos Tellería^55,56^; Thássia M.T.C. Carratto^324^; Jair Antonio Tenorio Castaño^2,9,10^; Alejandro Teper^119^; Izabel M.T. Araujo^233^; Juan Torres-Macho^327^; Lilian Torres-Tobar^120^; Ronald P. Torres Gutiérrez^298^; Jesús Troya^112^; Miguel Urioste^121^; Juan Valencia-Ramos^122^; Agustín Valido^18,328^; Juan Pablo Vargas Gallo^329,330^; Belén Varón^331^; Tomas Vega^332^; Santiago Velasco-Quirce^333^; Valentina Vélez-Santamaría^227,99^; Virginia Víctor^210,110^; Julia Vidán Estévez^106^; Gabriela V. Silva^233^; Miriam Vieitez-Santiago^62,64^; Carlos Vilches^334^; Lavinia Villalobos^106^; Felipe Villar^264^; Judit Villar-Garcia^335,336,337^; Cristina Villaverde^19,2^; Pablo Villoslada-Blanco^94^; Ana Virseda-Berdices^5^; Tatiana X. Costa^338^; Zuleima Yáñez^123^; Antonio Zapatero-Gaviria^339^; Ruth Zarate^124^; Sandra Zazo^312^; Carlos Flores^7,191,192^; José A. Riancho^62,63,64^; Augusto Rojas-Martinez^190^; Pablo Lapunzina^2,9,10^; Ángel Carracedo^1,2,8,3,4^

**SCOURGE Cohort Group affiliations:**

^1^, Centro Nacional de Genotipado (CEGEN), Universidade de Santiago de Compostela, Santiago de Compostela, Spain

^2^, Centre for Biomedical Network Research on Rare Diseases (CIBERER), Instituto de Salud Carlos III, Madrid, Spain

^3^, Instituto de Investigación Sanitaria de Santiago (IDIS), Santiago de Compostela, Spain

^4^, Centro Singular de Investigación en Medicina Molecular y Enfermedades Crónicas (CIMUS), Universidade de Santiago de Compostela, Santiago de Compostela, Spain

^5^, Unidad de Infección Viral e Inmunidad, Centro Nacional de Microbiología (CNM), Instituto de Salud Carlos III (ISCIII), Madrid, Spain

^6^, Spanish National Cancer Research Centre, Human Genotyping-CEGEN Unit, Madrid, Spain

^7^, Genomics Division, Instituto Tecnológico y de Energías Renovables, Santa Cruz de Tenerife, Spain

^8^, Fundación Pública Galega de Medicina Xenómica, Sistema Galego de Saúde (SERGAS) Santiago de Compostela, Spain

^9^, Instituto de Genética Médica y Molecular (INGEMM), Hospital Universitario La Paz-IDIPAZ, Madrid, Spain

^10^, ERN-ITHACA-European Reference Network

^11^, Unit of Infectious Diseases, Hospital Universitario 12 de Octubre, Instituto de Investigación Sanitaria Hospital 12 de Octubre (imas12), Madrid, Spain

^12^, Spanish Network for Research in Infectious Diseases (REIPI RD16/0016/0002), Instituto de Salud Carlos III, Madrid, Spain

^13^, School of Medicine, Universidad Complutense, Madrid, Spain

^14^, Centre for Biomedical Network Research on Infectious Diseases, Instituto de Salud Carlos III, Madrid, Spain

^15^, Hospital General Santa Bárbara de Soria, Soria, Spain

^16^, Pediatric Neurology Unit, Department of Pediatrics, Navarra Health Service Hospital, Pamplona, Spain

^17^, Navarra Health Service, NavarraBioMed Research Group, Pamplona, Spain

^18^, Hospital Universitario Virgen Macarena, Neumología, Seville, Spain

^19^, Department of Genetics & Genomics, Instituto de Investigación Sanitaria-Fundación Jiménez Díaz University Hospital - Universidad Autónoma de Madrid (IIS-FJD, UAM), Madrid, Spain

^20^, Department of Child and Adolescent Psychiatry, Institute of Psychiatry and Mental Health, Hospital General Universitario Gregorio Marañón (IiSGM), Madrid, Spain

^21^, Biocruces Bizkai HRI, Bizkaia, Spain

^22^, Cruces University Hospital, Osakidetza, Bizkaia, Spain

^23^, Centre for Biomedical Network Research on Mental Health (CIBERSAM), Instituto de Salud Carlos III, Madrid, Spain

^24^, Fundació Docència I Recerca Mutua Terrassa, Barcelona, Spain

^25^, Spanish National Cancer Research Center, CNIO Biobank, Madrid, Spain

^26^, Hospital General de Occidente, Zapopan Jalisco, Mexico

^27^, Centro Universitario de Tonalá, Universidad de Guadalajara, Tonalá Jalisco, Mexico

^28^, Centro de Investigación Multidisciplinario en Salud, Universidad de Guadalajara, Tonalá Jalisco, Mexico

^29^, Instituto Murciano de Investigación Biosanitaria (IMIB-Arrixaca), Murcia, Spain

^30^, Universidad Católica San Antonio de Murcia (UCAM), Murcia, Spain

^31^, Hospital Universitario de Salamanca-IBSAL, Servicio de Medicina Interna-Unidad de Enfermedades Infecciosas, Salamanca, Spain

^32^, Universidad de Salamanca, Salamanca, Spain

^33^, Escola Tecnica de Saúde, Laboratorio de Vigilancia Molecular Aplicada, Brazil

^34^, Federal University of Pernambuco, Genetics Postgraduate Program, Recife, PE, Brazil

^35^, Hospital Universitario Mutua Terrassa, Barcelona, Spain

^36^, Instituto de Investigación Sanitaria de Santiago (IDIS), Xenética Cardiovascular, Santiago de Compostela, Spain

^37^, Centre for Biomedical Network Research on Cardiovascular Diseases (CIBERCV), Instituto de Salud Carlos III, Madrid, Spain

^38^, Cardiovascular Genetics Center, Institut d’Investigació Biomèdica Girona (IDIBGI), Girona, Spain

^39^, Medical Science Department, School of Medicine, University of Girona, Girona, Spain

^40^, Hospital Josep Trueta, Cardiology Service, Girona, Spain

^41^, Institute of Biomedicine of Seville (IBiS), Consejo Superior de Investigaciones Científicas (CSIC)- University of Seville- Virgen del Rocio University Hospital, Seville, Spain

^42^, Departemento de Medicina, Hospital Universitario Virgen del Rocío,Universidad de Sevilla, Seville, Spain

^43^, Centre for Biomedical Network Research on Epidemiology and Public Health (CIBERESP), Instituto de Salud Carlos III, Madrid, Spain

^44^, Instituto de Biomedicina de Sevilla, Seville, Spain

^45^, Hospital Universitario de Salamanca-IBSAL, Servicio de Medicina Interna, Salamanca, Spain

^46^, Osakidetza, Cruces University Hospital, Bizkaia, Spain

^47^, Centre for Biomedical Network Research on Diabetes and Metabolic Associated Diseases (CIBERDEM), Instituto de Salud Carlos III, Madrid, Spain

^48^, University of Pais Vasco, UPV/EHU, Bizkaia, Spain

^49^, Oncology and Genetics Unit, Instituto de Investigacion Sanitaria Galicia Sur, Xerencia de Xestion Integrada de Vigo-Servizo Galego de Saúde, Vigo, Spain

^50^, Hospital Universitario Río Hortega, Valladolid, Spain

^51^, Servicio de Medicina intensiva, Complejo Hospitalario Universitario de A Coruña (CHUAC), Sistema Galego de Saúde (SERGAS), A Coruña, Spain

^52^, Tecnológico de Monterrey, Monterrey, Mexico

^53^, Otto von Guericke University, Departament of Microgravity and Translational Regenerative Medicine, Magdeburg, Germany

^54^, Hospital Universitario Mostoles, Unidad de Genética, Madrid, Spain

^55^, Instituto Aragonés de Ciencias de la Salud (IACS), Zaragoza, Spain

^56^, Instituto Investigación Sanitaria Aragón (IIS-Aragon), Zaragoza, Spain

^57^, Preventive Medicine Department, Instituto de Investigacion Sanitaria Galicia Sur, Xerencia de Xestion Integrada de Vigo-Servizo Galego de Saúde, Vigo, Spain

^58^, Unidad Diagnóstico Molecular. Fundación Rioja Salud, La Rioja, Spain

^59^, Universidade Federal do Rio Grande do Norte, Departamento de Analises Clinicas e Toxicologicas, Natal, Brazil

^60^, Hospital Universitario de Salamanca-IBSAL, Servicio de Cardiología, Salamanca, Spain

^61^, Instituto Regional de Investigación en Salud-Universidad Nacional de Caaguazú, Caaguazú, Paraguay

^62^, IDIVAL, Cantabria, Spain

^63^, Universidad de Cantabria, Cantabria, Spain

^64^, Hospital U M Valdecilla, Cantabria, Spain

^65^, Urgencias Hospitalarias, Complejo Hospitalario Universitario de A Coruña (CHUAC), Sistema Galego de Saúde (SERGAS), A Coruña, Spain

^66^, Grupo de Investigación en Interacciones Gen-Ambiente y Salud (GIIGAS) - Instituto de Biomedicina (IBIOMED), Universidad de León, León, Spain

^67^, Hospital Universitario de Getafe, Unidad de Genética, Madrid, Spain

^68^, Ministerio de Salud Ciudad de Buenos Aires, Buenos Aires, Argentina

^69^, Hospital Universitario Virgen de las Nieves, Servicio de Análisis Clínicos e Inmunología, Granada, Spain

^70^, IIS La Fe, Plataforma de Farmacogenética, Valencia, Spain

^71^, Universidad de Valencia, Departamento de Farmacología, Valencia, Spain

^72^, Data Analysis Department, Instituto de Investigación Sanitaria-Fundación Jiménez Díaz University Hospital - Universidad Autónoma de Madrid (IIS-FJD, UAM), Madrid, Spain

^73^, Universidad de los Andes, Facultad de Ciencias, Bogotá, Colombia

^74^, SIGEN Alianza Universidad de los Andes - Fundación Santa Fe de Bogotá, Bogotá, Colombia

^75^, Hospital General de Segovia, Medicina Intensiva, Segovia, Spain

^76^, Hospital Universitario 12 de Octubre, Department of Immunology, Madrid, Spain

^77^, Instituto de Investigación Sanitaria Hospital 12 de Octubre (imas12), Transplant Immunology and Immunodeficiencies Group, Madrid, Spain

^78^, Instituto de Biomedicina (IBIOMED), Universidad de León, León, Spain

^79^, Fundación Santa Fe de Bogota, Departamento Patologia y Laboratorios, Bogotá, Colombia

^80^, Unidad de Genética y Genómica Islas Baleares, Islas Baleares, Spain

^81^, Hospital Universitario Son Espases, Unidad de Diagnóstico Molecular y Genética Clínica, Islas Baleares, Spain

^82^, Genomics of Complex Diseases Unit, Research Institute of Hospital de la Santa Creu i Sant Pau, IIB Sant Pau, Barcelona, Spain

^83^, Universidade de Brasília, Faculdade de Medicina, Brazil

^84^, Programa de Pós-Graduação em Ciências Médicas (UnB), Brazil

^85^, Programa de Pós-Graduação em Ciencias da Saude (UnB), Brazil

^86^, Hospital El Bierzo, Unidad Cuidados Intensivos, León, Spain

^87^, Hospital Universitario Mostoles, Medicina Interna, Madrid, Spain

^88^, Universidad Francisco de Vitoria, Madrid, Spain

^89^, Departamento de Genética e Morfologia, Instituto de Ciências Biológicas, Universidade de Brasília, Brazil

^90^, Programa de Pós-Graduação em Biologia Animal (UnB), Brazil

^91^, Programa de Pós-Graduação Profissional em Ensino de Biologia (UnB), Brazil

^92^, Universidad Complutense de Madrid, Department of Immunology, Ophthalmology and ENT, Madrid, Spain

^93^, Universidade Federal do Pará, Núcleo de Pesquisas em Oncologia, Belém, Pará, Brazil

^94^, Infectious Diseases, Microbiota and Metabolism Unit, Center for Biomedical Research of La Rioja (CIBIR), Logroño, Spain

^95^, Inditex, A Coruña, Spain

^96^, GENYCA, Madrid, Spain

^97^, Instituto Mexicano del Seguro Social (IMSS), Centro Médico Nacional Siglo XXI, Unidad de Investigación Médica en Enfermedades Infecciosas y Parasitarias, Mexico City, Mexico

^98^, Clinica Comfamiliar Risaralda, Pereira, Colombia

^99^, Bellvitge Biomedical Research Institute (IDIBELL), Neurometabolic Diseases Laboratory, L’Hospitalet de Llobregat, Spain

^100^, Catalan Institution of Research and Advanced Studies (ICREA), Barcelona, Spain

^101^, Hospital Ophir Loyola, Departamento de Ensino e Pesquisa, Belém, Pará, Brazil

^102^, Unidad de Cuidados Intensivos, Hospital Clínico Universitario de Santiago (CHUS), Sistema Galego de Saúde (SERGAS), Santiago de Compostela, Spain

^103^, Department of Preventive Medicine and Public Health, School of Medicine, Universidad Autónoma de Madrid, Madrid, Spain

^104^, IdiPaz (Instituto de Investigación Sanitaria Hospital Universitario La Paz), Madrid, Spain

^105^, IMDEA-Food Institute, CEI UAM+CSIC, Madrid, Spain

^106^, Complejo Asistencial Universitario de León, León, Spain

^107^, Instituto de Investigación Biosanitaria de Granada (ibs GRANADA), Granada, Spain

^108^, Universidad de Granada, Departamento Bioquímica, Biología Molecular e Inmunología III, Granada, Spain

^109^, Hospital Infanta Elena, Allergy Unit, Valdemoro, Madrid, Spain

^110^, Instituto de Investigación Sanitaria-Fundación Jiménez Díaz University Hospital - Universidad Autónoma de Madrid (IIS-FJD, UAM), Madrid, Spain

^111^, Faculty of Medicine, Universidad Francisco de Vitoria, Madrid, Spain

^112^, Hospital Universitario Infanta Leonor, Madrid, Spain

^113^, Complutense University of Madrid, Madrid, Spain

^114^, Gregorio Marañón Health Research Institute (IiSGM), Madrid, Spain

^115^, Haemostasis and Thrombosis Unit, Hospital de la Santa Creu i Sant Pau, IIB Sant Pau, Barcelona, Spain

^116^, Hospital Clinico Universitario de Valladolid, Servicio de Anestesiologia y Reanimación, Valladolid, Spain

^117^, Universidad de Valladolid, Departamento de Cirugía, Valladolid, Spain

^118^, Hospital Clinico Universitario de Valladolid, Servicio de Hematologia y Hemoterapia, Valladolid, Spain

^119^, Hospital de Niños Ricardo Gutierrez, Buenos Aires, Argentina

^120^, Sociedad de Cirugía de Bogotá, Hospital de San José, Bogotá, Colombia

^121^, Spanish National Cancer Research Centre, Familial Cancer Clinical Unit, Madrid, Spain

^122^, University Hospital of Burgos, Burgos, Spain

^123^, Universidad Simón Bolívar, Facultad de Ciencias de la Salud, Barranquilla, Colombia

^124^, Centro para el Desarrollo de la Investigación Científica, Paraguay

^187^, Sección Genética Médica - Servicio de Pediatría, Hospital Clínico Universitario Virgen de la Arrixaca, Servicio Murciano de Salud, Murcia, Spain

^188^, Departamento Cirugía, Pediatría, Obstetricia y Ginecología, Facultad de Medicina, Universidad de Murcia (UMU), Murcia, Spain

^189^, Grupo Clínico Vinculado, Centre for Biomedical Network Research on Rare Diseases (CIBERER), Instituto de Salud Carlos III, Madrid, Spain

^190^, Tecnologico de Monterrey, Escuela de Medicina y Ciencias de la Salud, Monterrey, Mexico

^191^, Research Unit, Hospital Universitario N.S. de Candelaria, Santa Cruz de Tenerife, Spain

^192^, Centre for Biomedical Network Research on Respiratory Diseases (CIBERES), Instituto de Salud Carlos III, Madrid, Spain

^195^, Hospital Universitario San Pedro, Infectious Diseases Department, Logroño, Spain

^196^, Fundación Institut Guttmann, Institut Universitari de Neurorehabilitació adscrit a la UAB, Hospital de Neurorehabilitació, Barcelona, Spain

^197^, Universitat Autònoma de Barcelona (UAB), Barcelona, Spain

^198^, Fundació Institut d’Investigació en Ciències de la Salut Germans Trias i Pujol, Barcelona, Spain

^199^, Hospital General de Occidente, Guadalajara, Mexico

^200^, Microbiology Unit, Hospital Universitario N. S. de Candelaria, Santa Cruz de Tenerife, Spain

^201^, Hospital Universitario La Paz-IDIPAZ, Servicio de Neumología, Madrid, Spain

^202^, Camino Universitario Adelita de Char, Mired IPS, Barranquilla, Colombia

^203^, Hospital das Forças Armadas, Brazil

^204^, Exército Brasileiro, Brazil

^205^, Departamento de Medicina, Universidad de Salamanca, Salamanca, Spain

^206^, Centro de Investigación del Cáncer (IBMCC) Universidad de Salamanca - CSIC, Salamanca, Spain

^207^, Universidade Federal do Rio Grande do Norte, Programa de Pós-Graduação em Ciências Farmacêuticas, Natal, Brazil

^208^, Clinical Pharmacology Service, Hospital de la Santa Creu i Sant Pau, IIB Sant Pau, Barcelona, Spain

^209^, Servicio de Medicina Interna, Sanatorio Franchin, Buenos Aires, Argentina

^210^, Hospital Infanta Elena, Valdemoro, Madrid, Spain

^211^, Fundación Hospital Infantil Universitario de San José, Bogotá, Colombia

^212^, Fundación Universitaria de Ciencias de la Salud, Bogotá, Colombia

^213^, Universidade Federal do Rio Grande do Norte, Departamento de Analises Clínicas e Toxicológicas, Natal, Brazil

^214^, Colégio Marista de Brasilia, Brazil

^215^, Associação Brasileira de Educação e Cultura, Brazil

^216^, Hospital Universitario La Paz-IDIPAZ, Servicio de Medicina Interna, Madrid, Spain

^217^, Hospital Universitario de Fuenlabrada, Department of Internal Medicine, Madrid, Spain

^218^, Hospital Universitario Infanta Leonor, Servicio de Alergia, Madrid, Spain

^219^, Hospital Universitario del Tajo, Servicio de Medicina Intensiva, Toledo, Spain

^220^, Hospital Universitario La Paz-IDIPAZ, Servicio de Farmacología, Madrid, Spain

^221^, Alcaldía de Barranquilla, Secretaría de Salud, Barranquilla, Colombia

^222^, Division of Infectious Diseases, Instituto de Investigación Sanitaria-Fundación Jiménez Díaz University Hospital - Universidad Autónoma de Madrid (IIS-FJD, UAM), Madrid, Spain

^223^, ISGlobal, Barcelona, Spain

^224^, Universitat Pompeu Fabra (UPF), Barcelona, Spain

^225^, Intensive Care Unit, Hospital Universitario Insular de Gran Canaria, Las Palmas de Gran Canaria, Spain

^226^, Andalusian Public Health System Biobank, Granada, Spain

^227^, Neuromuscular Unit, Neurology Department, Hospital Universitari de Bellvitge, L’Hospitalet de Llobregat (Barcelona), Spain

^228^, Hospital Universitario La Paz, Hospital Carlos III, Madrid, Spain

^229^, Hospital de San José, Sociedad de Cirugía de Bogota, Bogotá, Colombia

^230^, Programa de Pós Graduação em Ciências da Saúde, Faculdade de Medicina, Universidade de Brasília, Brazil

^231^, Valencia University, Preventive Medicine Department, Valencia, Spain

^232^, Centre for Biomedical Network Research on Physiopatology of Obesity and Nutrition (CIBEROBN), Instituto de Salud Carlos III, Madrid, Spain

^233^, Universidade Federal do Rio Grande do Norte, Departamento de Analises Clinicais e Toxicologias, Natal, Brazil

^234^, Institute of Psychiatry and Mental Health, Hospital General Universitario Gregorio Marañón (IiSGM), Madrid, Spain

^235^, Hospital Universitario Lauro Wanderley, Brazil

^236^, Programa de Pós Graduação em Ciências Farmacêuticas (PPgCF), Natal, Brazil

^237^, Universidade Federal do Rio Grande do Norte, Programa de Pós-graduação em Ciências da Saúde, Natal, Brazil

^238^, Internal Medicine Department, Instituto de Investigación Sanitaria-Fundación Jiménez Díaz University Hospital - Universidad Autónoma de Madrid (IIS-FJD, UAM), Madrid, Spain

^239^, Hospital Universitario Severo Ochoa, Servicio de Medicina Interna, Madrid, Spain

^240^, Universidade Federal do Rio Grande do Norte, Programa de Pós-Graduação em Ciências da Saúde, Natal, Brazil

^241^, Universidade Federal do Rio Grande do Norte, Programa de Pós Graduação em Nutrição, Natal, Brazil

^242^, Hospital Universitario Virgen del Rocío, Servicio de Medicina Interna, Seville, Spain

^243^, Universidade Federal do Rio Grande do Norte, Departamento de Infectologia, Brazil

^244^, Hospital de Doenças Infecciosas Giselda Trigueiro, Rio Grande do Norte, Brazil

^245^, Faculdade de Ciências da Saúde, Universidade de Brasília, Brazil

^246^, Hospital Universitario Quironsalud Madrid, Madrid, Spain

^247^, Hospital Universitario Puerta de Hierro, Servicio de Medicina Interna, Majadahonda, Spain

^248^, Biocruces Bizkaia Health Research Institute, Galdakao University Hospital, Osakidetza, Bizkaia, Spain

^249^, Fundación Asilo San Jose, Cantabria, Spain

^250^, Unidad de Enfermedades Infecciosas, Servicio de Medicina Interna, Hospital Universitario Puerta de Hierro, Instituto de Investigación Sanitaria Puerta de Hierro - Segovia de Arana, Madrid, Spain

^251^, Universidad Nacional de Asunción, Facultad de Politécnica, Paraguay

^252^, Universidade de Fortaleza, Natal, Brazil

^253^, Hospital Universitario Niño Jesús, Pediatrics Department, Madrid, Spain

^254^, Unitat de Malalties Infeccioses i Importades, Servei de Pediatría, Infectious and Imported Diseases, Pediatric Unit, Hospital Universitari Sant Joan de Deú, Barcelona, Spain

^255^, Microbiology Department, Instituto de Investigación Sanitaria-Fundación Jiménez Díaz University Hospital - Universidad Autónoma de Madrid (IIS-FJD, UAM), Madrid, Spain

^256^, Department of Immunology, IRYCIS, Hospital Universitario Ramón y Cajal, Madrid, Spain

^257^, University of Salamanca, Biomedical Research Institute of Salamanca (IBSAL), Salamanca, Spain

^258^, Hospital Infanta Elena, Servicio de Medicina Intensiva, Valdemoro, Madrid, Spain

^259^, Programa de pós-graduação em biotecnologia - Rede Nordeste de Biotecnologia (RENORBIO), Universidade Federal do Rio Grande do Norte, Natal, Brazil

^260^, X1

^261^, Pneumology Department, Hospital General Universitario Gregorio Marañón (iiSGM), Madrid, Spain

^262^, Hospital Clinico Universitario de Valladolid, Unidad de Apoyo a la Investigación, Valladolid, Spain

^263^, Hospital Universitario Centro Dermatológico Federico Lleras Acosta, Bogotá, Colombia

^264^, Department of Neumology, Instituto de Investigación Sanitaria-Fundación Jiménez Díaz University Hospital - Universidad Autónoma de Madrid (IIS-FJD, UAM), Madrid, Spain

^265^, Sabin Medicina Diagnóstica, Brazil

^266^, Centro Universitario de Tonalá, Universidad de Guadalajara, Guadalajara, Mexico

^267^, Centro de Investigación Multidisciplinario en Salud, Universidad de Guadalajara, Guadalajara, Mexico

^268^, Unidad de Cuidados, Intensivos Hospital Clínico Universitario de Santiago (CHUS), Sistema Galego de Saúde (SERGAS), Santiago de Compostela, Spain

^269^, Hospital del Mar, Infectious Diseases Service, Barcelona, Spain

^270^, Institut Hospital del Mar d’Investigacions Mèdiques (IMIM), Barcelona, Spain

^271^, CEXS-Universitat Pompeu Fabra, Spanish Network for Research in Infectious Diseases (REIPI), Barcelona, Spain

^272^, Biocruces Bizkaia Health Research Institute, Basurto University Hospital, Osakidetza, Bizkaia, Spain

^273^, Opthalmology Department, Instituto de Investigación Sanitaria-Fundación Jiménez Díaz University Hospital - Universidad Autónoma de Madrid (IIS-FJD, UAM), Madrid, Spain

^274^, Hospital Sant Joan de Deu,Pediatric Critical Care Unit, Barcelona, Spain

^275^, Paediatric Intensive Care Unit, Agrupación Hospitalaria Clínic-Sant Joan de Déu, Esplugues de Llobregat, Barcelona, Spain

^276^, Clinical Trials Unit, Instituto de Investigación Sanitaria-Fundación Jiménez Díaz University Hospital - Universidad Autónoma de Madrid (IIS-FJD, UAM), Madrid, Spain

^277^, Hospital Universitario La Paz-IDIPAZ, Servicio de Inmunología, Madrid, Spain

^278^, La Paz Institute for Health Research (IdiPAZ), Lymphocyte Pathophysiology in Immunodeficiencies Group, Madrid, Spain

^279^, Hospital Universitario Virgen de las Nieves, Servicio de Enfermedades Infecciosas, Granada, Spain

^280^, Universidad de Granada, Departamento de Medicina, Granada, Spain

^281^, Intensive Care Unit, Hospital Universitario de Canarias, La Laguna, Spain

^282^, Dirección General de Salud Pública, Consejería de Sanidad, Junta de Castilla y León, Valladolid, Spain

^283^, Fundación Jiménez Díaz, Epidemiology, Madrid, Spain

^284^, Universidad Autónoma de Madrid, Department of Medicine, Madrid, Spain

^285^, Universidad de Valladolid, Departamento de Medicina, Valladolid, Spain

^286^, Preventive Medicine Department, Instituto de Investigación Sanitaria-Fundación Jiménez Díaz University Hospital - Universidad Autónoma de Madrid (IIS-FJD, UAM), Madrid, Spain

^287^, Intensive Care Unit, Hospital Universitario N. S. de Candelaria, Santa Cruz de Tenerife, Spain

^288^, Hospital Universitario Infanta Leonor, Servicio de Medicina Intensiva, Madrid, Spain

^289^, Hospital El Bierzo, Gerencia de Asistencia Sanitaria del Bierzo (GASBI), Gerencia Regional de Salud (SACYL), Ponferrada, Spain

^290^, Grupo INVESTEN, Instituto de Salud Carlos III, Madrid, Spain

^291^, Unidad de Cuidados Intensivos, Complejo Universitario de A Coruña (CHUAC), Sistema Galego de Saúde (SERGAS), A Coruña, Spain

^292^, Hospital Universitario La Paz-IDIPAZ, Servicio de Pediatría, Madrid, Spain

^293^, Instituto de Investigación Sanitaria San Carlos (IdISSC), Hospital Clínico San Carlos (HCSC), Madrid, Spain

^294^, Marinha do Brasil, Brazil

^295^, Universidade de Brasília, Brazil

^296^, Hospital General Universitario Gregorio Marañón (IiSGM), Madrid, Spain

^297^, Anatomía Patológica, Instituto de Investigación Sanitaria San Carlos (IdISSC), Hospital Clínico San Carlos (HCSC), Madrid, Spain

^298^, Hospital Nuestra Señora de Sonsoles, Ávila, Spain

^299^, Secretaria Municipal de Saude de Apodi, Natal, Brazil

^300^, Intensive Care Department, Instituto de Investigación Sanitaria-Fundación Jiménez Díaz University Hospital - Universidad Autónoma de Madrid (IIS-FJD, UAM), Madrid, Spain

^301^, Hospital Universitario Príncipe de Asturias, Servicio de Microbiología Clínica, Madrid, Spain

^302^, Universidad de Alcalá de Henares, Departamento de Biomedicina y Biotecnología, Facultad de Medicina y Ciencias de la Salud, Madrid, Spain

^303^, Genetics Unit, Department of Experimental and Health Sciences, Universitat Pompeu Fabra, Barcelona, Spain

^304^, Hospital del Mar and Hospital del Mar Research Institute (IMIM), Service of Genetics, Barcelona, Spain

^305^, Universidade Federal do Rio Grande do Norte, Departamento de Análises Clínicas e Toxicológicas, Natal, Brazil

^306^, Neuromuscular Diseases Unit, Department of Neurology, Hospital de la Santa Creu i Sant Pau, Universitat Autònoma de Barcelona, Barcelona, Spain

^307^, Drug Research Centre, Institut d’Investigació Biomèdica Sant Pau, IIB-Sant Pau, Barcelona, Spain

^308^, Faculdade de Medicina, Brazil

^309^, University Hospital Germans Trias i Pujol, Pediatrics Department, Badalona, Spain

^310^, Department of Immunology, Hospital Universitario de Gran Canaria Dr. Negrín, Las Palmas de Gran Canaria, Spain

^311^, Department of Clinical Sciences, University Fernando Pessoa Canarias, Las Palmas de Gran Canaria, Spain

^312^, Department of Pathology, Biobank, Instituto de Investigación Sanitaria-Fundación Jiménez Díaz University Hospital - Universidad Autónoma de Madrid (IIS-FJD, UAM), Madrid, Spain

^313^, Universidad de Sevilla, Departamento de Enfermería, Seville, Spain

^314^, Hospital Universitario Virgen de las Nieves, Servicio de Medicina Interna, Granada, Spain

^315^, Fundación Universitaria de Ciencias de la Salud, Grupo de Ciencias Básicas en Salud (CBS), Bogotá, Colombia

^316^, Reumathology Service, Instituto de Investigación Sanitaria-Fundación Jiménez Díaz University Hospital - Universidad Autónoma de Madrid (IIS-FJD, UAM), Madrid, Spain

^317^, Biobank, Puerta de Hierro-Segovia de Arana Health Research Institute, Madrid, Spain

^318^, Universidad Rey Juan Carlos, Madrid, Spain

^319^, X2

^320^, The John Walton Muscular Dystrophy Research Centre, Newcastle University and Newcastle Hospitals NHS Foundation Trust, Newcastle upon Tyne, UK.

^321^, Neuromuscular Unit, Neuropediatrics Department, Institut de Recerca Sant Joan de Déu, Hospital Sant Joan de Déu, Spain

^322^, Casa de Saúde São Lucas, Natal, Brazil

^323^, Hospital Rio Grande, Natal, Brazil

^324^, Departamento de Química, Faculdade de Filosofia, Ciências e Letras de Ribeirão Preto, Universidade de São Paulo, Brazil

^325^, Intensive Care Unit, Hospital Universitario de Gran Canaria Dr. Negrín, Las Palmas de Gran Canaria, Spain

^326^, Universidade Federal do Rio Grande do Norte, Pós-graduação em Biotecnologia - Rede de Biotecnologia do Nordeste (Renorbio), Natal, Brazil

^327^, Hospital Universitario Infanta Leonor, Servicio de Medicina Interna, Madrid, Spain

^328^, Universidad de Sevilla, Seville, Spain

^329^, Fundación Santa Fe de Bogota, Instituto de servicios medicos de Emergencia y trauma, Bogotá, Colombia

^330^, Universidad de los Andes, Bogotá, Colombia

^331^, Quironprevención, A Coruña, Spain

^332^, Junta de Castilla y León, Consejería de Sanidad, Valladolid, Spain

^333^, Gerencia Atención Primaria de Burgos, Burgos, Spain

^334^, Immunogenetics-Histocompatibility group, Servicio de Inmunología, Instituto de Investigación Sanitaria Puerta de Hierro - Segovia de Arana, Madrid, Spain

^335^, Hospital del Mar, Department of Infectious Diseases, Barcelona, Spain

^336^, IMIM (Hospital del Mar Medical Research Institute, Institut Hospital del Mar d’Investigacions Mediques), Barcelona, Spain

^337^, Universitat Autònoma de Barcelona, Department of Medicine, Spain

^338^, Maternidade Escola Janário Cicco, Natal, Brazil

^339^, Consejería de Sanidad, Comunidad de Madrid, Madrid, Spain

**HOSTAGE Cohort Group**

Aaron Blandino Ortiz^1^, Adolfo de Salazar^2,3^, Adolfo Garrido Chercoles^4^, Adriana Palom^5,6^, Agustín Albillos^7,8^, Agustín Ruiz^9,10^, Alba-Estela Garcia-Fernandez^11^, Albert Blanco-Grau^11^, Alberto Mantovani^12,13^, Alberto Zanella^14,15^, Aleksander Rygh Holten^16,17^, Alena Mayer^18^, Alessandra Bandera^14,15^, Alessandro Cherubini^15^, Alessandro Protti^12,13^, Alessio Aghemo^12,13^, Alessio Gerussi^19,20^, Alfredo Ramirez^21,22,23,24,25^, Alice Braun^18^, Almut Nebel^26^, Ana Barreira^6^, Ana Lleo^12,13^, Ana Teles^27,28^, Anders Benjamin Kildal^29^, Andre Franke^26,30^, Andrea Biondi^31^, Andrea Caballero-Garralda^11^, Andrea Gori^15,32^, Andreas Glück^33^, Andreas Lind^34^, Anja Tanck^26^, Anna Carreras Nolla^35^, Anna Latiano^36^, Anna Ludovica Fracanzani^14,15^, Anna Peschuck^26^, Annalisa Cavallero^37^, Anne Ma Dyrhol-Riise^17,38^, Antonella Ruello^39^, Antonio Julià^5^, Antonio Muscatello^15^, Antonio Pesenti^14,15^, Antonio Voza^12,13^, Ariadna Rando-Segura^40,41^, Aurora Solier^42^, Beatriz Cortes^35^, Beatriz Mateos^7,8^, Beatriz Nafria-Jimenez^4^, Benedikt Schaefer^43,44^, Bettina Heidecker^18^, Carla Bellinghausen^45^, Carlos Ferrando^46,47^, Carmen de la Horra^48,49,50,51,52^, Carmen Quereda^53^, Carsten Skurk^18^, Charlotte Thibeault^18^, Chiara Scollo^54^, Christoph Gassner^26,55^, Christoph Lange^56,57,58^, Cinzia Hu^15^, Cinzia Paccapelo^59^, Claudio Angelini^60^, Claudio Cappadona^13^, Clinton Azuure^27,28^, Cristiana Bianco^15^, Cristina Cea^11^, Cristina Sancho^61^, Dag Arne Lihaug Hoff^62,63^, Daniela Galimberti^14,15^, Daniele Prati^15^, David Ellinghaus^26,64^, David Haschka^65^, David Jiménez^42^, David Pestaña^66^, David Toapanta^47^, Douglas Maya-Miles^8,49,50^, Eduardo Muñiz-Diaz^67^, Eike M Wacker^26^, Elena Azzolini^12,13^, Elena Sandoval^47^, Eleonora Binatti^19,20^, Elio Scarpini^14,15^, Elisa T Helbig^18^, Eloisa Urrechaga^68,69^, Elvezia Maria Paraboschi^12,13^, Emanuele Pontali^70^, Enric Reverter^47^, Enrique J Calderón^48,49,50,51,52^, Enrique Navas^53^, Erik Solligård^71,72^, Ernesto Contro^73^, Eunate Arana^74^, Fátima Aziz^47^, Federico Garcia^2,3^, Félix García Sánchez^75^, Ferruccio Ceriotti^15^, Filippo Martinelli-Boneschi^76,77^, Flora Peyvandi^78,79^, Florian Kurth^18,80^, Florian Tran^26,33^, Florian Uellendahl-Werth^26^, Francesco Blasi^81,82^, Francesco Malvestiti^14^, Francisco J Medrano^48,49,51,52,83^, Francisco Mesonero^7,8^, Francisco Rodriguez-Frias^5,8^,^41,84^, Frank Hanses^85,86^, Frauke Degenhardt^26^, Fredrik Müller^17,34^, Georg Hemmrich-Stanisak^26^, Giacomo Bellani^87,88^, Giacomo Grasselli^14,15^, Gianni Pezzoli^89^, Giorgio Costantino^14,15^, Giovanni Albano^39^, Giulia Cardamone^90^, Giuseppe Bellelli^88,91^, Giuseppe Citerio^88,92^, Giuseppe Foti^87,88^, Giuseppe Lamorte^15^, Giuseppe Matullo^93^, Guido Baselli^59^, Hayato Kurihara^60^, Heinz Zoller^43,44^, Hesham ElAbd^26^, Holger Neb^94^, Ilaria My^12^, Isabel Hernández^9,10^, Itziar de Rojas^9,10^, Iván Galván-Femenia^35^, Jan C Holter^17,34^, Jan Egil Afset^62,95^, Jan Heyckendorf^56,57,58^, Jan Kässens^26^, Jan Kristian Damås^96,97^, Jatin Arora^98,99,100,101,102^, Javier Ampuero^48,49,50,103^, Javier Fernández^47,104^, Javier Martín^105^, Jeanette Erdmann^106,107,108^, Jesus M Banales^8,109,110,111^, Joan Ramon Badia^112^, Joaquin Dopazo^113^, Johannes R Hov^17,114,115,116^, Jon Lerga-Jaso^117^, Jonas Bergan^118^, Jordi Barretina^119^, Jose Hernández Quero^2,120^, Josune Goikoetxea^121^, Juan Delgado^48,49,50,51,52^, Juan M Guerrero^48,49,50^, Julia Kraft^18^, Kari Risnes^97,122^, Karina Banasik^64^, Karl Erik Müller^123^, Karoline I Gaede^124,125,126^, Koldo Garcia-Etxebarria^8,111^, Kristian Tonby^17,38^, Lars Heggelund^123,127^, Lars Wienbrandt^26^, Laura Izquierdo-Sanchez^8,111,128^, Laura Rachele Bettini^31^, Lauro Sumoy^119^, Leif Erik Sander^18^, Lena J Lippert^18^, Leonardo Terranova^15^, Lise Tuset Gustad^71,129^, Luca Valenti^14,15^, Lucia Garbarino^130^, Luigi Santoro^15^, Luigia Scudeller^59^, Luis Bujanda^8,109,111^, Luis Téllez^7,8^, Luisa Roade^6,8^,^41^, Mahnoosh Ostadreza^15^, Maider Intxausti^61^, Malte C Rühlemann^26,131^, Manolis Kogevinas on behalf of the COVICAT study group^51,132,133,134^, Manuel Romero-Gómez^8,135^, Mar Riveiro-Barciela^6,8^,^41^, Marco Schaefer^136^, Mareike Wendorff^26^, María A Gutiérrez-Stampa^137^, Maria Buti^6,8^,^41^, Maria Carrabba^59^, Maria E. Figuera Basso^26^, Maria Grazia Valsecchi^138^, María Hernandez-Tejero^47^, Maria JGT Vehreschild^139^, Maria Manunta^15^, Marialbert Acosta-Herrera^105^, Mariella D'Angiò^31^, Marina Baldini^59^, Marina Cazzaniga^140^, Mario Cáceres^117,141^, Marit M Grimsrud^17,115,116^, Marta Marquié^9,10^, Massimo Castoldi^39^, Maurizio Cecconi^12,13^, Mauro D'Amato^110,142^, May Sissel Vadla^143,144^, Melissa Tomasi^15^, Mercè Boada^9,10^, Michael Joannidis^145^, Michael Wittig^26^, Michela Mazzocco^130^, Michele Ciccarelli^60^, Miguel Rodríguez-Gandía^7,8^, Monica Bocciolone^60^, Monica Miozzo^14,59^, Natale Imaz Ayo^74^, Natalia Blay^35^, Natalia Chueca^3^, Nicola Montano^14,15^, Nicole Braun^26,146^, Nilda Martínez^147^, Ole Bernt Lenning^143,148^, Oliver A Cornely^149,150,151,152^, Onur Özer^27,28^, Orazio Palmieri^36^, Paola Faverio^88,153^, Paoletta Preatoni^60^, Paolo Bonfanti^88,154^, Paolo Omodei^60^, Paolo Tentorio^12^, Pedro Castro^47^, Pedro M Rodrigues^8,111,128^, Pedro Pablo España^68^, Per Hoffmann^155^, Petra Bacher^26,156^, Philip Rosenstiel^26^, Philipp Koehler^149,150,157^, Phillip Suwalski^18^, Pietro Invernizzi^19,20^, Rafael de Cid on behalf of the COVICAT study group^35^, Raúl de Pablo^1^, Ricard Ferrer^158^, Roberta Gualtierotti^14,15^, Rocío Gallego-Durán^49,50,103^, Ronny Myhre^159^, Rosa Nieto^42^, Rosanna Asselta^12,13^, Rossana Carpani^15^, Rubén Morilla^48,49,50,51,52^, Salvatore Badalamenti^12^, Sammra Haider^160^, Sandra Ciesek^161,162^, Sandra May^26^, Sara Bombace^12,13^, Sara Marsal^5^, Sebastian Klein^145^, Serena Aneli^93^, Serena Pelusi^14,15^, Sibylle Wilfling^86,163,164^, Siegfried Goerg^165^, Silvano Bosari^14,15^, Simonas Juzenas^26,166^, Søren Brunak^64^, Soumya Raychaudhuri^98,99,100,101,102,167^, Stefan Schreiber^26,33^, Stefanie Heilmann-Heimbach^155^, Stefano Aliberti^12,13^, Stefano Duga^12,13^, Stephan Ripke^18^, Susanne Dudman^17,34^, Tanja Wesse^26^, Tenghao Zheng^168^, Thomas Bahmer^33^, Tobias L Lenz^27,28^, Tom H Karlsen^17,114,115,116^, Tomas Pumarola^169,170^, Trine Folseraas^17,114,115,116^, Trinidad Gonzalez Cejudo^171^, Ulf Landmesser^172^, Ute Hehr^163^, Valeria Rimoldi^13^, Valter Monzani^59^, Vegard Skogen^173,174^, Vicente Friaza^48,49,50,51,52^, Victor Andrade^21,23^, Victor Moreno^51,175,176,177^, Wolfgang Albrecht^26^, Wolfgang Peter^136^, Wolfgang Poller^18^, Xavier Farre^35^, Xiaoli Yi^26^, Xiaomin Wang^18^, Ximo Dopazo^178^, Yascha Khodamoradi^139^, Zehra Karadeniz^18^, COVICAT study group, Covid-19 Aachen Study (COVAS), Pa COVID-19 Study Group, The Humanitas COVID-19 Task Force, The Humanitas Gavazzeni COVID-19 Task Force

**HOSTAGE Cohort Group affiliations:**

^1^ Department of Intensive Care, Hospital Universitario Ramón y Cajal, Instituto Ramón y Cajal de Investigación Sanitaria (IRYCIS), University of Alcalá, Madrid, Spain.

^2^ Ibs.ranada Instituto de Investigación Biosanitaria, Granada, Spain.

^3^ Microbiology Unit.Hospital Univeristario Clinico San Cecilio, Granada, Spain.

^4^ Osakidetza Basque Health Service, Donostialdea Integrated Health Organisation, Clinical Biochemistry Department, San Sebastian, Spain.

^5^ Vall d’Hebron Institut de Recerca (VHIR), Vall d’Hebron Hospital Universitari, Barcelona, Spain.

^6^ Liver Unit, Department of Internal Medicine, Hospital Universitari Vall d’Hebron, Vall d’Hebron Barcelona Hospital Campus, Barcelona, Spain.

^7^ Department of Gastroenterology, Hospital Universitario Ramón y Cajal, University of Alcalá, Instituto Ramón y Cajal de Investigación Sanitaria (IRYCIS), Madrid, Spain.

^8^ Centro de Investigación Biomédica en Red en Enfermedades Hepáticas y Digestivas (CIBEREHD), Instituto de Salud Carlos III (ISCIII), Madrid, Spain.

^9^ Research Center and Memory Clinic.Ace Alzheimer Center Barcelona – Universitat Internacional de Catalunya, Spain.

^10^ CIBERNED, Network Center for Biomedical Research in Neurodegenerative Diseases, National Institute of Health Carlos III, Madrid, Spain.

^11^ Department of Biochemistry, University Hospital Vall d’Hebron, Barcelona, Spain.

^12^ IRCCS Humanitas Research Hospital, Rozzano, Milan, Italy.

^13^ Department of Biomedical Sciences, Humanitas University, Pieve Emanuele, Milan, Italy.

^14^ University of Milan, Milan, Italy.

^15^ Fondazione IRCCS Ca' Granda Ospedale Maggiore Policlinico, Milan, Italy.

^16^ Department of Acute Medicine, Oslo University Hospital, Oslo, Norway.

^17^ Institute of Clinical Medicine, University of Oslo, Oslo, Norway.

^18^ Charite Universitätsmedizin Berlin, Berlin, Germany.

^19^ European Reference Network on Hepatological Diseases (ERN RARE-LIVER), San Gerardo Hospital, Monza, Italy.

^20^ Division of Gastroenterology, Center for Autoimmune Liver Diseases, School of Medicine and Surgery, University of Milano-Bicocca, Milan, Italy.

^21^ Department of Neurodegenerative Diseases and Geriatric Psychiatry, University Hospital Bonn, Medical Faculty, Bonn, Germany.

^22^ Excellence Cluster on Cellular Stress Responses in Aging-Associated Diseases (CECAD), University of Cologne, Cologne, Germany.

^23^ Division of Neurogenetics and Molecular Psychiatry, Department of Psychiatry and Psychotherapy, Faculty of Medicine and University Hospital Cologne, University of Cologne, Cologne, Germany.

^24^ German Center for Neurodegenerative Diseases (DZNE), Bonn, Germany.

^25^ Department of Psychiatry and Glenn Biggs Institute for Alzheimer’s and Neurodegenerative Diseases, San Antonio, TX, USA.

^26^ Institute of Clinical Molecular Biology, Christian-Albrechts-University, Kiel, Germany.

^27^ Research Group for Evolutionary Immunogenomics, Max Planck Institute for Evolutionary Biology, Plön, Germany.

^28^ Research Unit for Evolutionary Immunogenomics, Department of Biology, University of Hamburg, Hamburg, Germany.

^29^ Department of Anesthesiology and Intensive Care, University Hospital of North Norway, Tromsø, Norway.

^30^ University Hospital Schleswig-Holstein (UKSH), Campus Kiel, Germany.

^31^ Pediatric Departement and Centro Tettamanti- European Reference Network (ERN) PaedCan, EuroBloodNet, MetabERN-University of Milano-Bicocca-Fondazione MBBM/Ospedale San Gerardo, Italy.

^32^ Centre for Multidisciplinary Research in Health Science (MACH), University of Milan, Milan, Italy.

^33^ Klinik für Innere Medizin I, Universitätsklinikum Schleswig-Holstein, Campus Kiel, Germany.

^34^ Department of Microbiology, Oslo University Hospital, Oslo, Norway.

^35^ Genomes for Life-GCAT lab.Germans Trias i Pujol Research Institute (IGTP), Badalona, Spain.

^36^ Gastroenterology Unit, Fondazione IRCCS Casa Sollievo della Sofferenza, San Giovanni Rotondo, Italy.

^37^ Laboratory of Microbiology, San Gerardo Hospital, Monza, Italy.

^38^ Department of Infectious diseases, Oslo University Hospital, Oslo, Norway.

^39^ Humanitas Gavazzeni-Castelli, Bergamo, Italy.

^40^ Microbiology Department, Hospital Universitari Vall d'Hebron, Barcelona, Spain.

^41^ Universitat Autònoma de Barcelona, Bellatera, Spain.

^42^ Department of Respiratory Diseases, Hospital Universitario Ramón y Cajal, Instituto Ramón y Cajal de Investigación Sanitaria (IRYCIS), University of Alcalá, Centro de Investigación Biomédica en Red en Enfermedades Respiratorias (CIBERES), Madrid, Spain.

^43^ Medical University of Innsbruck, Department of Medicine I, Gastroenterology, Hepatology and Endocrinology, Innsbruck, Austria.

^44^ Christian Doppler Laboratory of Iron and Phosphate Biology at the Department of Medicine I, Medical University of Innsbruck, Innsbruck, Austria.

^45^ Department of Respiratory Medicine and Allergology, University Hospital, Goethe University, Frankfurt am Main, Germany.

^46^ Centro de Investigación Biomédica en Red de Enfermedades Respiratorias (CIBERES), Madrid, Spain.

^47^ Hospital Clinic, University of Barcelona, and IDIBAPS, Barcelona, Spain.

^48^ University of Sevilla, Sevilla, Spain.

^49^ Instituto de Biomedicina de Sevilla (IBIS), Sevilla, Spain.

^50^ Hospital Universitario Virgen del Rocío de Sevilla, Sevilla, Spain.

^51^ Centro de Investigación Biomédica en Red de Epidemiología y Salud Pública (CIBERESP), Madrid, Spain.

^52^ Consejo Superior de Investigaciones científicas, Sevilla, Spain.

^53^ Department of Infectious Diseases, Hospital Universitario Ramón y Cajal, Instituto Ramón y Cajal de Investigación Sanitaria (IRYCIS), University of Alcalá, Madrid, Spain.

^54^ Department of Transfusion Medicine and Haematology Laboratory, San Gerardo Hospital, Monza, Italy.

^55^ Private University in the Principality of Liechtenstein.

^56^ Respiratory Medicine & International Health, University of Lübeck, Lübeck, Germany.

^57^ Division of Clinical Infectious Diseases, Research Center Borstel, Borstel, Germany.

^58^ German Center for Infection Research (DZIF) Clinical Tuberculosis Unit, Borstel, Germany.

^59^ Fondazione IRCCS Ca’ Granda Ospedale Maggiore Policlinico, Milan, Italy.

^60^ Humanitas Clinical and Research Center, IRCCS, Milan, Italy.

^61^ Osakidetza Basque Health Service, Basurto University Hospital, Respiratory Service, Bilbao, Spain.

^62^ Department of Clinical and Molecular Medicine, Faculty of Medicine and Health Sciences, Norwegian University of Science and Technology, Trondheim, Norway.

^63^ Department of Medicine, Møre & Romsdal Hospital Trust, Ålesund, Norway.

^64^ Novo Nordisk Foundation Center for Protein Research, Disease Systems Biology, Faculty of Health and Medical Sciences, University of Copenhagen, Copenhagen, Denmark.

^65^ Department of Internal Medicine II, Medical University of Innsbruck, Innsbruck, Austria.

^66^ Department of Anesthesiology and Critical Care, Hospital Universitario Ramón y Cajal, Instituto Ramón y Cajal de Investigación Sanitaria (IRYCIS), University of Alcalá, Madrid, Spain.

^67^ Immunohematology Department, Banc de Sang i Teixits, Autonomous University of Barcelona, Barcelona, Spain.

^68^ Osakidetza Basque Health Service, Galdakao Hospital, Respiratory Service, Galdakao, Spain.

^69^ Biocruces Bizkaia Health Research Institute.

^70^ Department of Infectious Diseases - E..Ospedali Galliera, Genova, Italy.

^71^ Geminicenter for Sepsis Research, Institute of Circulation and Medical Imaging (ISB), NTNU, Trondheim, Norway.

^72^ Clinic of Anesthesia and Intensive Care, St Olavs Hospital, Trondheim University Hospital, Trondheim, Norway.

^73^ Accident & Emergency and Emergency Medicine Unit, San Gerardo Hospital, Monza, Italy.

^74^ Biocruces Bizkaia Health Research Institute, Barakaldo, Spain.

^75^ Histocompatibilidad y Biologia Molecular, Centro de Transfusion de Madrid, Madrid, Spain.

^76^ Dino Ferrari Center, Department of Pathophysiology and Transplantation, University of Milan, Milan, Italy.

^77^ IRCCS Fondazione Ca' Granda Ospedale Maggiore Policlinico, Neurology Unit, Milan, Italy.

^78^ University of Milan, Department of Pathophysiology and Transplantation, Milan, Italy.

^79^ Fondazione IRCCS Ca' Granda Ospedale Maggiore Policlinico, Angelo Bianchi Bonomi Hemophilia and Thrombosis Center, Milan, Italy.

^80^ Department of Tropical Medicine, Bernhard Nocht Institute for Tropical Medicine, and Department of Medicine I, University Medical Centre Hamburg-Eppendorf, ^20359^ Hamburg, Germany.

^81^ Fondazione IRCCS Ca' Granda Ospedale Maggiore Policlinico, Respiratory Unit, Milan, Italy.

^82^ Department of Pathophysiology and Transplantation, Università degli Studi di Milano, Italy.

^83^ Internal Medicine Department, Virgen del Rocio University Hospital, Sevilla, Spain.

^84^ Biochemistry Department, University Hospital Vall d'Hebron, Barcelona, Spain.

^85^ Emergency Department, University Hospital Regensburg, Regensburg, Germany.

^86^ Department for Infectious Diseases and Infection Control, University Hospital Regensburg, Regensburg, Germany.

^87^ Department Emergency, Anesthesia and Intensive Care, San Gerardo Hospital, Monza, Italy.

^88^ School of Medicine and Surgery, University of Milano-Bicocca, Milan, Italy.

^89^ Fondazione Grigioni per il Morbo di Parkinson and Parkinson Institute, ASST Gaetano Pini-CTO, Milan, Italy.

^90^ Department of Biomedical Sciences, Humanitas University, Milan, Italy.

^91^ Acute Geriatric Unit, San Gerardo Hospital, Monza, Italy.

^92^ Neurointensive Care Unit, San Gerardo Hospital, Monza, Italy.

^93^ Department of Medical Sciences, Università degli Studi di Torino, Turin, Italy.

^94^ Department of Anesthesiology, Intensive Care Medicine and Pain Therapy, University Hospital Frankfurt, Frankfurt am Main, Germany.

^95^ Department of Medical Microbiology, Clinic of Laboratory Medicine, St.Olavs hospital, Trondheim, Norway.

^96^ Department of Infectious Diseases, St Olavs Hospital, Trondheim University Hospital, Trondheim, Norway.

^97^ Department of Clinical and Molecular Medicine, NTNU, Trondheim, Norway.

^98^ Program in Medical and Population Genetics, Broad Institute of MIT and Harvard, Cambridge, MA, USA.

^99^ Division of Rheumatology, Inflammation and Immunity, Brigham and Women’s Hospital and Harvard Medical School, Boston, MA, USA.

^100^ Division of Genetics, Department of Medicine, Brigham and Women’s Hospital, Boston, MA, USA.

^101^ Department of Biomedical Informatics, Harvard Medical School, Boston, MA, USA.

^102^ Center for Data Sciences, Brigham and Women’s Hospital, Boston, MA, USA.

^103^ Centro de Investigación Biomédica en Red Enfermedades Hepáticas y Digestivas (CIBEREHD), Sevilla, Spain.

^104^ European Foundation for the Study of Chronic Liver Failure (EF-CLIF), Barcelona, Spain.

^105^ Institute of Parasitology and Biomedicine Lopez-Neyra, Granada, Spain.

^106^ Institute for Cardiogenetics, University of Lübeck, Lübeck, Germany.

^107^ German Research Center for Cardiovascular Research, partner site Hamburg–Lübeck–Kiel, Lübeck, Germany.

^108^ University Heart Center Lübeck, Lübeck, Germany.

^109^ Department of Liver and Gastrointestinal Diseases, Biodonostia Health Research Institute – Donostia University Hospital, University of the Basque Country (UPV/EHU), CIBERehd, Ikerbasque, San Sebastian, Spain.

^110^ Ikerbasque, Basque Foundation for Science, Bilbao, Spain.

^111^ Department of Liver and Gastrointestinal Diseases, Biodonostia Health Research Institute – Donostia University Hospital, University of the Basque Country (UPV/EHU), San Sebastian, Spain.

^112^ Respiratory ICU, Institut Clínic Respiratory, Hospital Clinic, University of Barcelona, and IDIBAPS, Barcelona, Spain.

^113^ Bioinformatics Area, Fundación Progreso y Salud, and Instritute of Biomedicine of Sevilla (IBIS), Sevilla, Spain.

^114^ Section for Gastroenterology, Department of Transplantation Medicine, Division for Cancer Medicine, Surgery and Transplantation, Oslo University Hospital Rikshospitalet, Oslo, Norway.

^115^ Research Institute for Internal Medicine, Division of Surgery, Inflammatory Diseases and Transplantation, Oslo University Hospital Rikshospitalet and University of Oslo, Oslo, Norway.

^116^ Norwegian PSC Research Center, Department of Transplantation Medicine, Division of Surgery, Inflammatory Diseases and Transplantation, Oslo University Hospital Rikshospitalet, Oslo, Norway.

^117^ Institut de Biotecnologia i de Biomedicina, Universitat Autònoma de Barcelona, Bellaterra (Barcelona), Spain.

^118^ Department of Research, Ostfold Hospital Trust, Gralum, Norway.

^119^ Germans Trias i Pujol Research Institute (IGTP), Badalona, Spain.

^120^ Department of Infectious Diseases, Hospital Univeristario Clinico San Cecilio, Granada, Spain.

^121^ Infectious Diseases Service, Osakidetza, Biocruces Bizkaia Health Research Institute, Barakaldo, Spain.

^122^ Department of Research, St Olav Hospital, Trondheim University Hospital, Trondheim, Norway.

^123^ Medical Department, Drammen Hospital, Vestre Viken Hospital Trust, Norway.

^124^ Research Center Borstel, BioMaterialBank Nord, Germany.

^125^ German Center for Lung Research (DZL), Airway Research Center North (ARCN), Germany.

^126^ Popgen ^2.^ network (P^2N^), Kiel, Germany.

^127^ Department of Clinical Science, University of Bergen, Bergen, Norway.

^128^ Biodonostia Health Research Institute, Donostia University Hospital, San Sebastian, Spain.

^129^ Clinic of Medicine and Rehabilitation, Levanger Hospital, Nord-Trondelag Hospital Trust, Levanger, Norway.

^130^ HLA Laboratory – E..Ospedali Galliera, Genova, Italy.

^131^ Institute for Medical Microbiology and Hospital Epidemiology, Hannover Medical School, Hannover, Germany.

^132^ ISGlobal, Barcelona, Spain.

^133^ Universitat Pompeu Fabra (UPF), Barcelona, Spain.

^134^ IMIM (Hospital del Mar Medical Research Institute), Barcelona, Spain.

^135^ Digestive Diseases Unit, Virgen del Rocio University Hospital, Institute of Biomedicine of Seville, University of Seville, Seville, Spain.

^136^ Stefan-Morsch-Stiftung, Birkenfeld, Germany.

^137^ Osakidetza, OSI Donostialdea, Altza Primary Care, Biodonostia Health Research Institute, San Sebastián, Spain.

^138^ Center of Bioinformatics, Biostatistics and Bioimaging, School of Medicine and Surgery, University of Milano-Bicocca, Milan, Italy.

^139^ Department of Internal Medicine, Infectious Diseases, University Hospital Frankfurt & Goethe University Frankfurt, Frankfurt am Main, Germany.

^140^ Phase ^1^ Research Centre, ASST Monza, School of Medicine and Surgery, University of Milano-Bicocca, Italy.

^141^ ICREA, Barcelona, Spain.

^142^ Gastrointestinal Genetics Lab, CIC bioGUNE - BRTA, Derio, Spain.

^143^ Randaberg Municipality, Norway.

^144^ University of Stavanger, Faculty of Health Sciences, Department of Quality and Health Technology, Stavanger, Norway.

^145^ Division of Intensive Care and Emergency Medicine, Department of Internal Medicine, Medical University Innsbruck, Innsbruck, Austria.

^146^ University Hospital Schleswig-Holstein, Campus Kiel, Kiel, Germany.

^147^ Department of Anesthesiology, Hospital Universitario Ramón y Cajal, Instituto Ramón y Cajal de Investigación Sanitaria (IRYCIS), Madrid, Spain.

^148^ Research Department, Stavanger University Hospital.

^149^ University of Cologne, Medical Faculty and University Hospital Cologne, Department I of Internal Medicine, Cologne, Germany.

^150^ University of Cologne, Cologne Excellence Cluster on Cellular Stress Responses in Aging-Associated Diseases (CECAD), Cologne, Germany.

^151^ Clinical Trials Centre Cologne, ZKS Köln, Cologne, Germany.

^152^ University of Cologne, Medical Faculty and University Hospital Cologne, German Center for Infection Research (DZIF), Partner Site Bonn-Cologne, Cologne, Germany.

^153^ Pulmonary Unit, San Gerardo Hospital, Monza, Italy.

^154^ Infectious Diseases Unit, San Gerardo Hospital, Monza, Italy.

^155^ Institute of Human Genetics, University of Bonn School of Medicine & University Hospital Bonn, Bonn, Germany.

^156^ Institute of Immunology, Christian-Albrechts-University of Kiel & UKSH Schleswig-Holstein, Kiel, Germany.

^157^ Center for Molecular Medicine Cologne (CMMC), University of Cologne, Cologne, Germany.

^158^ Intensive Care Department, Vall d'Hebron University Hospital, SODIR-VHIR research group, Barcelona, Spain.

^159^ Norwegian Institute of Public Health, Division of Health Data and Digitalization, Department of Genetics and Bioinformatics (HDGB) Oslo, Norway.

^160^ Department of Medicine, Møre & Romsdal Hospital Trust, Molde, Norway.

^161^ Institute of Medical Virology, University Hospital Frankfurt, Goethe University, Frankfurt am Main, Germany.

^162^ German Centre for Infection Research (DZIF), External Partner Site Frankfurt, Frankfurt am Main, Germany.

^163^ Zentrum für Humangenetik Regensburg, Regensburg, Germany.

^164^ Department of Neurology, Bezirksklinikum Regensburg, University of Regensburg, Regensburg, Germany.

^165^ Institute of Transfusionsmedicine, University Hospital Schleswig-Holstein (UKSH), Germany.

^166^ Institute of Biotechnology, Life Science Centre, Vilnius University, Lithuania.

^167^ Centre for Genetics and Genomics Versus Arthritis, Centre for Musculoskeletal Research, Manchester Academic Health Science Centre, The University of Manchester, Manchester, UK.

^168^ School of Biological Sciences, Monash University, Clayton, VIC, Australia.

^169^ Department of Microbiology, University Hospital Vall d’Hebron, Barcelona, Spain.

^170^ Autonoma University of Barcelona, Barcelona, Spain.

^171^ Biochemistry Unit.Hospital Univeristario Clinico San Cecilio, Granada, Spain.

^172^ Charite Universitätsmedizin Berlin, Berlin Institute of Health, Berlin Germany.

^173^ Department of Infectious Diseases, University Hospital of North Norway, Tromsø, Norway.

^174^ Faculty of Health Sciences, UIT The Arctic University of Norway, Norway.

^175^ Catalan Institute of Oncology (ICO), Barcelona, Spain.

^176^ Bellvitge Biomedical Research Institute (IDIBELL), Barcelona, Spain.

^177^ Universitat de Barcelona (UB), Barcelona, Spain.

^178^ Bioinformatics area, Fiundación progreso y Salud, Andalucia, Spain.

**GR@ACE Cohort Group**

The GR@ACE study group
N. Aguilera, E. Alarcon, M. Alegret, M. Boada, M. Buendia, A. Cano, P. Cañabate, A. Carracedo, A. Corbatón-Anchuelo, I. de Rojas, S. Diego, A. Espinosa, A. Gailhajenet, P. García-González, M. Guitart, A. González-Pérez, M. Ibarria, A. Lafuente, J. Macias, O. Maroñas, E. Martín, M. T. Martínez, M. Marquié, L. Montrreal, S. Moreno-Grau, M. Moreno, R. Nuñez-Llaves, C. Olivé ,A. Orellana, G. Ortega, A. Pancho, E. Pelejá, A. Pérez-Cordon, J. A. Pineda, R. Puerta, S. Preckler, I. Quintela, L. M. Real, M. Rosende-Roca, A. Ruiz, M. E. Sáez, A. Sanabria, M. Serrano-Rios, O. Sotolongo-Grau, L. Tárraga, S. Valero & L. Vargas

DEGESCO consortium
A. D. Adarmes-Gómez, E. Alarcón-Martín, M. D. Alonso, I. Álvarez, V. Álvarez, G. Amer-Ferrer, M. Antequera, C. Antúnez, M. Baquero, M. Bernal, R. Blesa, M. Boada, D. Buiza-Rueda, M. J. Bullido, J. A. Burguera, M. Calero, F. Carrillo, M. Carrión-Claro, M. J. Casajeros, J. Clarimón, J. M. Cruz-Gamero, M. M. de Pancorbo, I. de Rojas, T. del Ser, M. Diez-Fairen, R. Escuela, L. Garrote-Espina, J. Fortea, E. Franco-Macías, A. Frank-García, J. M. García-Alberca, S. Garcia Madrona, G. Garcia-Ribas, P. Gómez-Garre, S. Hevilla, S. Jesús, M. A. Labrador Espinosa, C. Lage, A. Legaz, A. Lleó, A. Lopez de Munain, S. López-García, D. Macias-García, S. Manzanares, M. Marín, J. Marín-Muñoz, T. Marín, M. Marquié, A. Martín Montes, B. Martínez, C. Martínez, V. Martínez, P. Martínez-Lage Álvarez, M. Medina, M. Mendioroz Iriarte, M. Menéndez-González, P. Mir, L. Montrreal, A. Orellana, P. Pastor, J. Pérez Tur, T. Periñán-Tocino, R. Pineda-Sanchez, G. Piñol-Ripoll, A. Rábano, D. Real de Asúa, S. Rodrigo, E. Rodríguez-Rodríguez, J. L. Royo, A. Ruiz, R. Sanchez del Valle Díaz, P. Sánchez-Juan, I. Sastre, O. Sotolongo-Grau, S. Valero, M. P. Vicente, R. Vigo-Ortega & L. Vivancos.

## **Other Scourge Cohort Group members (not directly participating in this manuscript)**

Gonzalo Acebes^1,2^; Isabel Acosta^3^; Manuela A. Albuquerque^4^; Eduardo Alonso^5^; David Alonso Menchen D^6^; Julián Álvarez Escudero^7^; Ana C. Antolí Royo^8^; Gabriela S. Arcanjo^4^; Ivan A. Arce-Cardenas^9^; Víctor J. Asensio^10,11^; Georgios Athanasiadis^12^; Gabriela Avila^3^; Aurora Baluja^7,13^; M. Alejandro Barajas-Zambrano^9^; Rafael Benito^14,2^; Fernando Bergaz^15^; Marcelino Bermudez-Lopez^16^; Vanesa Bernal^1,2^; Pahola Bogado^17^; Lidia Bonilla-Melendo L^18^; Luis Borderías^19^; Gabriel Bretones^20^; José Miguel Bruñén^1,2^; Hider Cabrera Martínez^8^; Mario Cáceres^21,22^; Serafí Cambray^16^; Yenddy Carrero^23^; Diego Casas^1,2^; Esteban Castelao^24,25^; Rocío Cebollada^19^; Juan Churruca^26^; Oscar Oscar Coltell^27,28,29^; Ángel Concha^30^; Cristina Córdoba-Chicote^31^; Guillermo Cuevas^32^; Aluísio X. Magalhães-Brasil^33^; Pilar Delgado^1,2^; Mariel Denise^34^; José María Domingo^35^; Igor F. Domingos^4,36^; María Jesús Domínguez^37^; Joaquín Dopazo^38,39,40^; José Antonio Enriquez^41,42^; Inés E. Arijón^43^; Samyra E. Lima^44^; M. Esther Esteban^12,45^; Diana Ezquerro-Pérez^46^; Verónica E. Fermín Ramírez^8^; Ana Fernández-Santander^47^; María L. Ferreira-Laso^48^; Ana Ferreiro^43^; Pilar Figueras^1,2^; Raquel Flores Peirats^49^; Máximo Fraga^50^; David Freire^51^; María Gaibar^47^; Cristóbal Galbán Rodíguez^52^; José L. García-Allut^52^; Concepción García-Lacalle^31^; María A. Garcinuño Jiménez^8^; Natalia Gascon-Ramon^15^; Luis Giménez^37^; Josune Gokioetxea^53^; Sonia G. Gomez-Navarro^54,55^; Juan R. González^56,57,58^; Fabricio González-Andrade^59^; Arturo González-Quintela^60^; Charo González^61^; Graciela González^62^; Sabrina G. Paiva^63,64,65^; Alexander D. Heine^10,11^; Juan Gregorio Hernandez^66^; Miguel A. Huete Diego^67^; Fabio A. Jaimes Bautista^8^; María Concepción Jiménez Gómez^41^; Maria Ángeles Julián^14,2^; Pedro Latorre^1,2^; Maria José Lavilla^19^; Jorge Lázaro-Galán^68^; Beatriz Lema^69^; Jon Lerga-Jaso^21^; José López Castro^70^; Carlos López-Otín^20,71^; Alejandro López-Soto^20^; Cecilia Alexandra Lozano-Sandoval^72^; Antonio R. Lucena-Araujo^4^; Brunno G.S. Macedo^4^; Maria José Marín^61,73^; Amanda C.M. Saúde^74,75^; José U. Márquez^76,77^; Eduardo Martinez^1,2^; Luis Martínez^14,2^; Patricia Alejandra A. Martínez Pérez^8^; Maria Isabel Millán^5^; Jesús Millán Nuñez-Cortés^67^; Dolores Miramar^1,2^; Sara Miranda Ponte^24,25^; Maria Luisa Monforte^5^; Juliana Nardelli-Costa^78,65^; Daniel A. Navarro-Alcalá^79^; Pablo Noseda^66^; Apolonia Novillo^47^; Lorena Ocampos^17^; Javier Ortega Andreu^8^; José Ramón Paño^14,2^; Dolors Pelegri-Siso^56,57,58^; María Peña-Chilet^38,39,40^; Hugo Pérez-Freixo^80^; Javier Perez-Florido^38,39^; José P. Freije^20,71^; Sergio Pérez Pinto^8^; José G. G. Pérez-Silva^20^; Aline Pic-Taylor^81,82,83,84^; Alejandro P. Ugalde^20^; Antonio Pose-Reino^60^; Gloria Prado-Alonso^9^; Diana A. Puente^20^; Marta Puig^21^; José W. Quenata Romero^8^; Víctor Quesada^20,71^; Leticia Ramirez^3^; Marta Rava^85^; Carmen Redondo Marey^24,25^; Luis Rello^1,2^; Antonio Rezusta^1,2^; Pilar Robres^86^; Lourdes Roc^1,2^; David Rodríguez^20,71^; Eva M. Rodríguez Beltrán^8^; Eduardo Rodriguez-Urcelay^15^; David Roiz-Valle^20^; Alicia Romero-Lorca^47^; Luis A. Romero-Padilla^9^; Paulina Rubio-Lara^72^; Carlos Ruíz-Arenas^56,57,58^; Hector Alejandro Salazar^87^; Alicia F. Salvatierra Maldonado^8^; María Saura^88^; Laura Sayagués Moreira^52^; Guillermo Sequera^89^; Trinidad Serrano^14,2^; Zulema Silguero^90^; Beatriz Sobrino^91,13^; Celso T. Mendes-Junior^92,93,94^; Monica T. Andrade^95,74^; Luis Torres^19^; Laura Torres^10,11^; Antón Trigo González^8^; Alexandro Tristancho^1,2^; Elena Urcelay^96^; José M. Valdivielso^16^; Jorge Valencia^32^; Olaia Velasco^53^; Illya Yakymenko^21^; Ruth Zarate^97^; Nuria Zazo^53^; Teresa Zitto^66^; Marcos Zuil^5^

### **Scourge Cohort Group members, not participating in this manuscript, affiliations:**

^1^, Hospital Universitario Miguel Servet, Zaragoza, Spain

^2^, Instituto Investigación Sanitaria Aragón (IIS-Aragon), Zaragoza, Spain

^3^, Universidad Nacional de Asunción, Facultad de Ciencias Médicas, Paraguay

^4^, Federal University of Pernambuco, Genetics Postgraduate Program, Recife, PE, Brazil

^5^, Hospital Royo Villanova, Zaragoza, Spain

^6^, Hospital Universitario Príncipe de Asturias, Servicio de Medicina Interna, Madrid, Spain

^7^, Servicio de Anestesiología, Reanimación y Tratamiento del Dolor, Hospital Clínico Universitario de Santiago (CHUS), Sistema Galego de Saúde (SERGAS), Santiago de Compostela, Spain

^8^, Hospital Nuestra Señora de Sonsoles, Ávila, Spain

^9^, Hospital General de Occidente, Guadalajara, Mexico

^10^, Unidad de Genética y Genómica Islas Baleares, Islas Baleares, Spain

^11^, Hospital Universitario Son Espases, Unidad de Diagnóstico Molecular y Genética Clínica, Islas Baleares, Spain

^12^, Sección Zoología y Antropología Biológica, Departamento de Biología Evolutiva, Ecología y Ciencias Ambientales, Facultad de Biología, Universitat de Barcelona, Barcelona, Spain

^13^, Instituto de Investigación Sanitaria de Santiago (IDIS) , Santiago de Compostela, Spain

^14^, Hospital Clínico Universitario Lozano Blesa, Zaragoza, Spain

^15^, GENYCA, Madrid, Spain

^16^, Institut de Recerca Biomèdica de Lleida Fundació Dr. Pifarré, Lleida, Spain

^17^, Ministerio de Salud Pública y Bienestar Social, Dirección de la V Región Sanitaria, Paraguay

^18^, Hospital Universitario Severo Ochoa, Servicio de Medicina Interna, Madrid, Spain

^19^, Hospital San Jorge, Zaragoza, Spain

^20^, Departamento de Bioquímica y Biología Molecular - IUOPA, Universidad de Oviedo, Oviedo, Spain

^21^, Institut de Biotecnologia i de Biomedicina, Universitat Autònoma de Barcelona, Bellaterra (Barcelona), Spain

^22^, Catalan Institution of Research and Advanced Studies (ICREA), Barcelona, Spain

^23^, Facultad de Ciencias de la Salud, Carrera de Medicina, Universidad Técnica de Ambato, Ambato, Ecuador

^24^, Hospital Álvaro Cunqueiro, Sistema Galego de Saúde (SERGAS), Vigo, Spain

^25^, Instituto de Investigación Biomédica Galicia Sur, Vigo, Spain

^26^, Hospital Universitario Infanta Leonor, Servicio de Hematologia, Madrid, Spain

^27^, Valencia University, Preventive Medicine Department, Valencia, Spain

^28^, Centre for Biomedical Network Research on Physiopatology of Obesity and Nutrition (CIBEROBN), Instituto de Salud Carlos III, Madrid, Spain

^29^, Jaume I University, Department of computer sciences, Madrid, Spain

^30^, Servicio de Anatomía Patológica, Hospital Clínico Universitario de A Coruña (CHUAC), Sistema Galego de Saúde (SERGAS), A Coruña, Spain

^31^, Hospital Severo Ochoa, Servicio de Análisis Clínicos y Bioquímica Clínica, Madrid, Spain

^32^, Hospital Universitario Infanta Leonor, Servicio de Medicina Interna, Madrid, Spain

^33^, Universidade de Brasília, Faculdade de Medicina, Brazil

^34^, Hospital Regional de Coronel Oviedo, Ministerio de Salud Pública y Bienestar Social, Paraguay

^35^, Banco de Sangre y tejidos de Aragón, Zaragoza, Spain

^36^, Federal University of Rio Grande do Norte, Department of Clinical and Toxicological Analysis, Natal, RN, Brazil

^37^, Hospital Universitario Virgen del Rocío, Servicio de Medicina Interna, Seville, Spain

^38^, Clinical Bioinformatics Area, Fundación Progreso y Salud (FPS), Hospital Virgen del Rocio, Seville, Spain

^39^, Computational Systems Medicine, Institute of Biomedicine of Seville (IBIS), Hospital Virgen del Rocio, Seville, Spain

^40^, Bioinformatics in Rare Diseases (BiER), Centre for Biomedical Network Research on Rare Diseases (CIBERER), Instituto de Salud Carlos III, Madrid, Spain

^41^, Fundación Centro Nacional de Investigaciones Cardiovasculares Carlos III CNIC, Madrid, Spain

^42^, Centre for Biomedical Network Research for Frailty and Healthy Ageing (CIBERFES), Instituto de Salud Carlos III, Madrid, Spain

^43^, Unidad de Cuidados Intensivos, Hospital Universitario Lucus Augusti (HULA), Sistema Galego de Saúde (SERGAS), Lugo, Spain

^44^, Programa de Pós Graduação em Biologia Animal, Instituto de Ciências Biológicas, Universidade de Brasília, Brazil

^45^, Institut de Recerca de la Biodiversitat (IRBio), Universitat de Barcelona, Barcelona, Spain.

^46^, Hospital Universitario San Pedro, Infectious Diseases Department, Logroño, Spain

^47^, Universidad Europea de Madrid, Madrid, Spain

^48^, Hospital Universitario San Pedro, Department of Anesthesiology and Postoperative Care, Logroño, Spain

^49^, XX5

^50^, Hospital Clínico Universitario de Santiago (CHUS), Sistema Galego de Saúde (SERGAS), Santiago de Compostela, Spain

^51^, Unidad de Cuidados Intensivos, Hospital Clínico Universitario de A Coruña (CHUAC), Sistema Galego de Saúde (SERGAS), A Coruña, Spain

^52^, Unidad de Cuidados Intensivos Hospital Clínico Universitario de Santiago (CHUS), Sistema Galego de Saúde (SERGAS), Santiago de Compostela, Spain

^53^, Biocruces Bizkai HRI, Bizkaia, Spain

^54^, Centro Universitario de Tonalá, Universidad de Guadalajara, Guadalajara, Mexico

^55^, Centro de Investigación Multidisciplinario en Salud, Universidad de Guadalajara, Guadalajara, Mexico

^56^, ISGlobal, Barcelona, Spain

^57^, Universitat Pompeu Fabra (UPF), Barcelona, Spain

^58^, Centre for Biomedical Network Research on Epidemiology and Public Health (CIBERESP), Instituto de Salud Carlos III, Madrid, Spain

^59^, Facultad de Ciencias Médicas, Universidad Central del Ecuador, Unidad de Medicina Traslacional, Quito, Ecuador

^60^, Complejo Hospitalario Universitario de Santiago, Department of Internal Medicine, Santiago de Compostela, Spain

^61^, IDIVAL, Cantabria, Spain

^62^, CIMAT, Department of Probability and Statistics, Mexico

^63^, Instituto Federal de Educação, Ciência e Tecnologia do Tocantins (IFTO), Campus Araguaína, Tocantins, Brazil

^64^, Universidade Federal do Tocantins, Araguaína, Brazil

^65^, Departamento de Genética e Morfologia, Instituto de Biologia, Universidade de Brasília, Brazil

^66^, MyDNAmap, Gipuzkoa, Spain

^67^, Hospital Gregorio Marañón, Madrid, Spain

^68^, Center for Biomedical Research of La Rioja (CIBIR), Investigation Respiratory Diseases Unit, Logroño. Spain

^69^, Hospital Clínico Universitario de A Coruña (CHUAC), Sistema Galego de Saúde (SERGAS), A Coruña, Spain

^70^, Servicio de Medicina Interna, Hospital Público de Monforte, Sistema Galego de Saúde (SERGAS), Lugo, Spain

^71^, Centre for Biomedical Network Research on Cancer (CIBERONC), Instituto de Salud Carlos III, Madrid, Spain

^72^, Tecnológico de Monterrey, Monterrey, Mexico

^73^, Instituto de Investigación Marqués de Valdecilla, Cantabria, Spain

^74^, Exército Brasileiro, Brazil

^75^, Colégio Militar de Brasília, Brazil

^76^, Centro de Investigación en Matemáticas, Unidad Monterrey, Mexico

^77^, Consejo Nacional de Ciencia y Tecnología, Mexico

^78^, QIAGEN Aarhus, Denmark

^79^, Centro Universitario de Ciencias de la Salud, Universidad de Guadalajara, Guadalajara, Mexico

^80^, Servicio de Medicina Preventiva. Hospital Clínico Universitario de Santiago (CHUS), Sistema Galego de Saúde (SERGAS), Santiago de Compostela, Spain

^81^, Departamento de Genética e Morfologia, Instituto de Ciências Biológicas, Universidade de Brasília, Brazil

^82^, Programa de Pós-Graduação em Biologia Animal (UnB), Brazil

^83^, Programa de Pós-Graduação em Ciencias da Saude (UnB), Brazil

^84^, Programa de Pós-Graduação em Ciências Médicas (UnB), Brazil

^85^, Unidad de la Cohorte de la Red de Investigación en Sida (CoRIS). Centro Nacional de Epidemiología (CNE). Instituto de Salud Carlos III (ISCIII), Madrid, Spain

^86^, Hospital de Barbastro, Zaragoza, Spain

^87^, Clinica Comfamiliar Risaralda, Pereira, Colombia

^88^, Instituto Nacional de Investigación y Tecnología Agraria y Alimentaria, Departamento de Mejora Genética Animal, Madrid, Spain

^89^, Ministerio de Salud Pública y Bienestar Social, Dirección de Vigilancia de la Salud, Paraguay

^90^, Instituto Regional de Investigación en Salud, Paraguay

^91^, Fundación Pública Galega de Medicina Xenómica, Sistema Galego de Saúde (SERGAS) Santiago de Compostela, Spain

^92^, Departamento de Química, Faculdade de Filosofia, Ciências e Letras de Ribeirão Preto, Universidade de São Paulo, Brazil

^93^, Programa de Pós-Graduação em Genética da Facudade de Medicina de Ribeirão Preto, Brazil

^94^, Programa de Pós-Graduação em Química da Faculdade de Filosofia, Ciências e Letras de Ribeirão Preto, Brazil

^95^, Hospital das Forças Armadas, Brazil

^96^, Lab. Genetics and Molecular Basis of Complex Diseases, Instituto de Investigación Sanitaria del Hospital Clínico San Carlos, IdISSC, Madrid, Spain

^97^, Centro para el Desarrollo de la Investigación Científica, Paraguay
